# Upregulation of GADD45*α* in light-damaged retinal pigment epithelial cells

**DOI:** 10.1038/cddiscovery.2016.13

**Published:** 2016-02-29

**Authors:** M-L Gao, W-L Deng, N Huang, Y-Y Wang, X-L Lei, Z-Q Xu, D-N Hu, J-Q Cai, F Lu, Z-B Jin

**Affiliations:** 1 Lab for Stem Cells and Retinal Regeneration, Division of Ophthalmic Genetics, Institute for Stem Cell Research, The Eye Hospital of Wenzhou Medical University, State Key Laboratory Cultivation Base and Key Laboratory of Vision Science, Ministry of Public Health, Wenzhou 325027, China; 2 Tissue Culture Center, New York Eye and Ear Infirmary, New York Medical College, Valhalla, 10595 New York, USA; 3 China National Institute of Standardization, Beijing 100191, China

## Abstract

To better understand the molecular mechanisms responsible for light-induced damage in retinal pigmented epithelial (RPE) cells, we developed an automated device to recapitulate intense light exposure. When compared with human fibroblasts, ARPE-19 cells that had been exposed to blue-rich light-emitting diode-light of 10 000 Lux at 37 °C for 9 h displayed dramatic cellular apoptosis. Collectively, gene expression profiling and qPCR demonstrated that growth arrest and DNA damage-45*α* (GADD45*α*) expression was markedly upregulated. Transient knockdown of GADD45*α* partially attenuated light-damage-induced apoptosis in ARPE-19 cells, whereas GADD45*α* overexpression dramatically increased it. These results demonstrate the critical function of GADD45*α* in light-induced RPE cellular apoptosis. Quantitative reverse transcription-PCR and western blotting revealed that the upregulation of GADD45*α* was under direct control of p53. Moreover, treatment with Ly294002, an inhibitor of AKT phosphorylation, further promoted GADD45*α* gene transcription in both non-light and light-damaged ARPE-19 cells. Treatment also exacerbated RPE cellular apoptosis after light exposure, confirming that inhibition of Akt phosphorylation increases GADD45*α* expression. Collectively, our findings reveal that light irrigation induces human RPE cellular apoptosis through upregulation of GADD45*α* expression mediated through both the p53 and phosphatidylinositol 3-kinase-AKT signaling pathways. These results provide new insights into human retinal diseases elicited by light damage and open a new avenue for disease prevention and treatment.

It has long been recognized that excessive light exposure produces photochemical lesions in the retina, ultimately leading to the damage of retinal pigmented epithelial (RPE) cells and the neural retina.^1,2^ Results from previous studies suggest that light-induced RPE damage may be one of the most important factors associated with age-related macular degeneration, solar retinopathy, and other retinal degenerative lesions.^3–6^ To this end, RPE cells are particularly susceptible to wavelengths within the blue region of the spectrum.^7,8^ Despite this, many aspects of light-induced damage to RPE cells remain unclear, with the biological mechanisms behind such damage virtually unknown.

As shown in a variety of past studies, the apoptotic pathway is the main avenue for light-induced cell death,^9,10^ which then led to a pathway including execution and modulation by Caspase-3 and Bcl-2, respectively.^11–13^ Furthermore, previous work has demonstrated that not only is a caspase-dependent apoptotic pathway involved in RPE cellular apoptosis, but also variety of cellular compartments, including mitochondria, lysosomes, and proteasomes.^14–16^

Recently, light pollution has become increasingly more common with the advent of new technologies and devices in domestic lighting, which has drawn the attention of researchers in the retinal degeneration field.^17^ Among these new devices, the most widely used are light-emitting diodes (LEDs), which present the greatest concern. On the technical level, LEDs have many advantages, including long life and low energy consumption. However, the LED spectrum range includes and delivers intense blue light components to the retina—more intense than normal daylight or more conventional domestic lighting sources.^18^ Although previous studies have focused on how blue light damages RPE cells, the effect of LEDs on the retina still requires much work, with particular focus needed for the molecular mechanisms behind such damage.

In this study, we have developed an instrument with controllable light intensity and temperature to imitate light-induced apoptosis on RPE cells. Using this *in vitro* surrogate, we found serious RPE cellular apoptosis resulting from extensive exposure to blue-intensive LED light. We then found increased expression of growth arrest and DNA damage-45*α* (GADD45*α*), a marker of DNA damage, which plays a critical role in the progression of light-induced RPE cellular apoptosis. In addition, the upregulation of GADD45*α* may be directly regulated by the p53 signaling pathway and indirectly by the phosphatidylinositol 3-kinase (PI3K)-AKT signaling pathway. Taken together, our research offers a novel target for light-induced visual impairment.

## Results

### Lighting system

**Figure 1:**
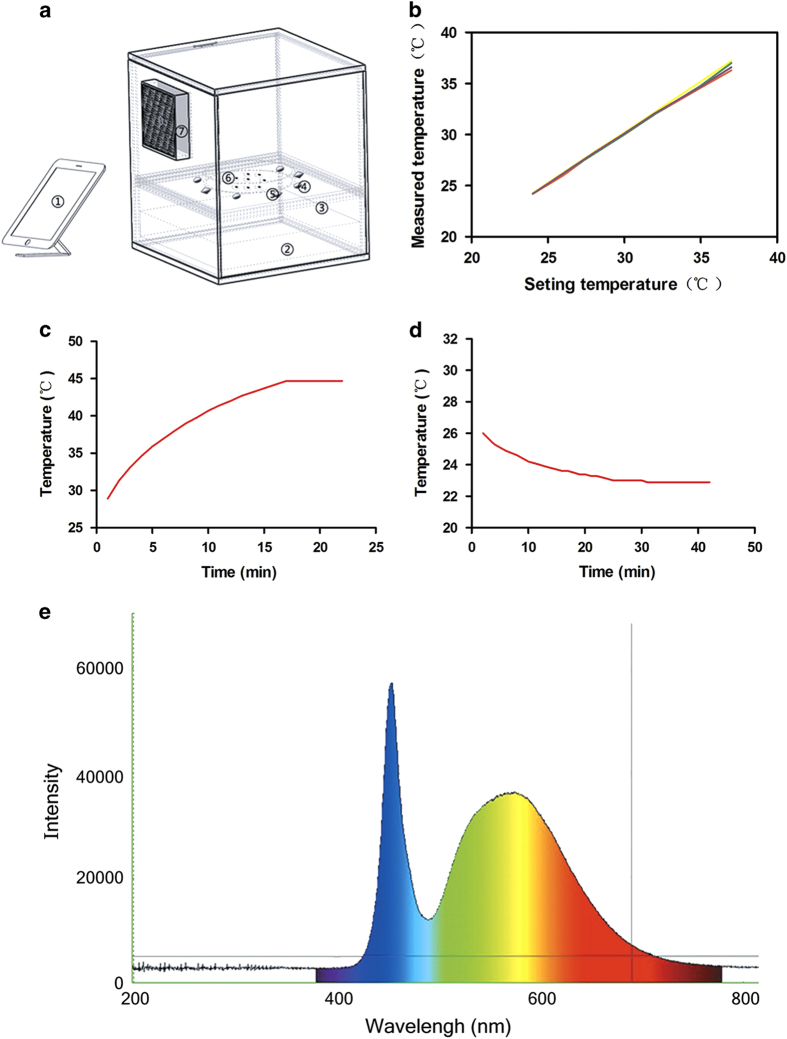
Instrumental architecture of the homothermal lighting system. (**a**) Components of the instrument include: (1) display control screen, (2) LED light source, (3) optical filter, (4) temperature sensors, (5) light-intensity sensors, (6) dish-drop zone, and (7) semiconductor refrigeration tablets. (**b**) The four lines represent the four points on which temperatures were monitored by four sensors, showing high consistency in all four. (**c**) 15 min is required to completely warm the dish space from 20 to 45 °C. (**d**) 30 min is required to completely and stably lower the temperature from 27.2 to 20 °C. (**e**) The action spectrum of the LED light, as determined spectrometrically.

Our *SmartLight2.0* instrument with controllable light intensity and temperature was developed to allow for direct and intense blue-rich LED light exposure to cultured cells (Figure 1a). As shown in Table 1, the instrumental parameters, including light intensity, temperature adjustment, temperature error, and light error were validated in triplicate. The temperature of four different areas showed no spatial variation (Figure 1b), with any variations returning quickly to the set value (Figure 1c and d). In addition, light intensity could be set to any value at or below 12 000 Lux. The LED light action spectrum is shown in Figure 1e, with peaks occurring predominantly at the wavelength of blue light (470 nm). Taken together, these results demonstrate that *SmartLight2.0* has stable, controllable parameters, making it a feasible instrument to model light-induced damage *in vitro*.

### Light-induced apoptosis in human RPE cells

**Figure 2:**
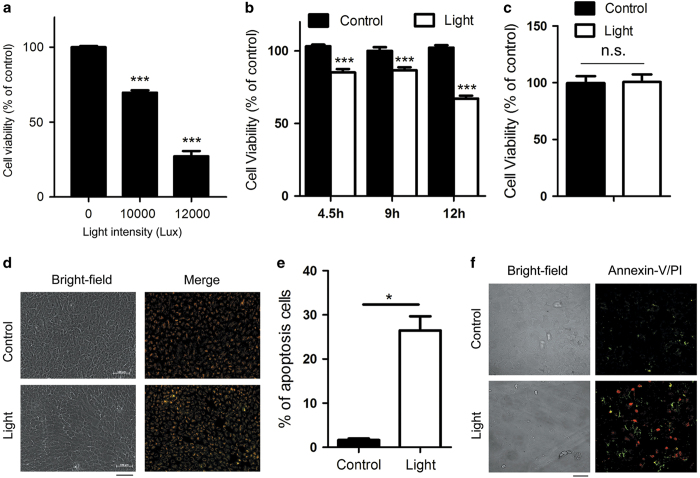
Exposure to LED light induced apoptosis in ARPE-19 cells. The water-soluble tetrazolium salt (WST-1) test was used to test cell viability after light exposure. (**a**) ARPE-19 cells were exposed to white light at different light intensities, with resulting cell viability decreasing in a dose-dependent manner. Cell viability value are given as mean value±S.E.M. (*n*=5, ***P<0.001). (**b**) Time-dependent cytotoxic effects of light exposure on ARPE-19 cells. Data are reported as mean value±S.E.M. (*n*=10, ***P<0.001). (**c**) Primary human fibroblasts exposed to 9 h of 10 000 Lux light did not show apoptosis. Each value represents mean value±S.E.M. (*n*=7). (**d**) Cells stained with JC-1 dye, an indicator of mitochondrial membrane potential. The light-damaged RPE cells (9 h at 10 000 Lux) displayed a significant decrease in mitochondrial membrane potential. The apoptotic cells showed low mitochondrial membrane potential and the monomer JC-1 was distributed in the cytoplasm (green). The aggregated JC-1 in mitochondrial exhibited red fluorescence. Bar=100 *μ*m. (**e**) The percentage of apoptosis was calculated based on the area of the JC-1 monomer distribution shown in **d**. Results are presented as mean value±S.E.M. (*n*=3, *P<0.05). (**f**) Apoptosis of light-damaged ARPE-19 cells was analyzed with Annexin-V/PI staining. Annexin-V single positive (green) cells are apoptotic cells and Annexin-V/PI double staining or PI single staining (red) cells are undergoing late-stage apoptosis/necrosis. Annexin-V/PI double negative cells are normal cells. Bar=50 *μ*m.

To determine the effect of intense LED light on RPE cells in our system, we used ARPE-19 cells along with differential light intensity exposure. We found that light intensity within a range of 10 000–12 000 Lux resulted in cell death (Figure 2a). Given this finding, we then determined cell viability in a time-dependent manners using a light intensity of 10 000 Lux. The viability of ARPE-19 cells treated with 4.5 h of 10 000 Lux light decreased to 88.0% when compared with non-light controls. Furthermore, the proportion of cells surviving declined as a function of increased exposure time (Figure 2b). Human fibroblasts were unaffected (Figure 2c).

To determine whether the decline in cellular viability was the result of cellular apoptosis, we performed both mitochondrial membrane potential and Annexin-V/propidium iodide (PI) double staining assays. The mitochondrial potential assay revealed that ARPE-19 cells underwent apoptosis after light exposure (Figure 2d). Moreover, that the percentage of apoptotic cells in the light-damaged condition was dramatically higher than in non-light controls (Figure 2e). In addition, Annexin-V/PI staining showed that late-stage apoptosis/necrosis was induced after 9 h of exposure to 10 000 Lux illumination (Figure 2f). These data collectively suggest that blue-rich LED light triggers apoptosis in ARPE-19 cells.

### Light activates GADD45*α* signaling

**Figure 3:**
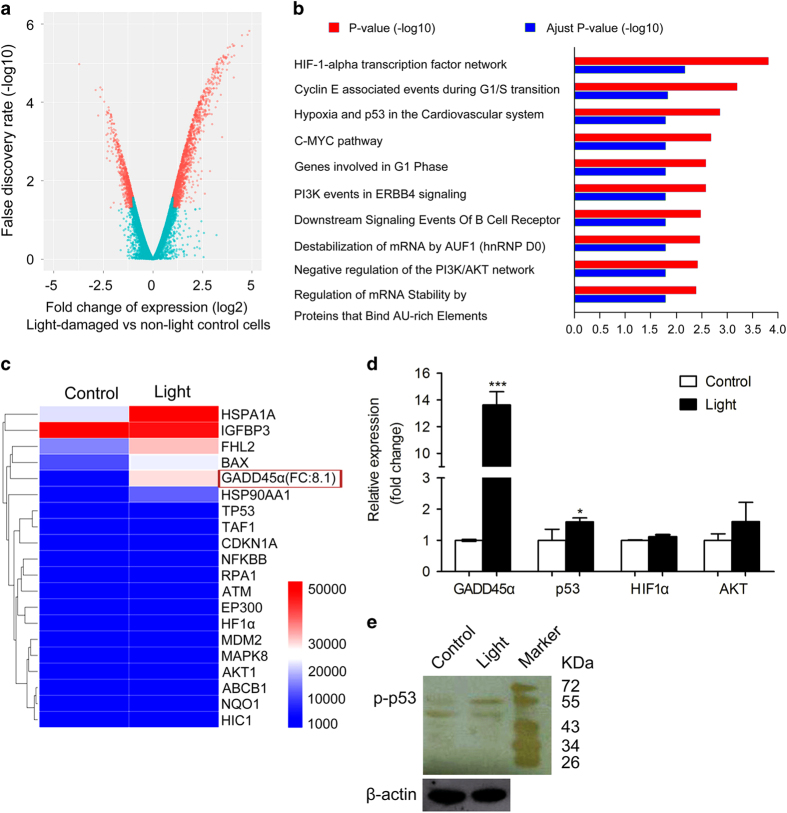
Gene expression profiling. (**a**) Schematic representations of the gene expression profiling in human OneArray experiments carried out in ARPE-19 cells. (**b**) Gene ontology (GO) analysis demonstrated differentially expressed pathways between non-light-damaged and light-damaged cells. (**c**) Gene expression signature of light-damaged ARPE-19 cells in the p53 hypoxia pathway. (**d**) Quantitative RT-PCR was performed to confirm the results from our GO analysis. mRNA levels were standardized to the control gene *β-actin*. Results are presented as mean value±S.E.M. (*n*=3, *P<0.05, ***P<0.001). (**e**) Western blotting showed upregulation of p-p53 in light-damaged cells. The *β*-actin loading control is from the same experiment as shown in Figure 5a and is reproduced in this figure for ease of reference.

To identify differentially regulated genes, we employed microarrays targeting human genes. Analyses of gene expression data showed that 2467 genes were differentially regulated after light illumination. When compared with non-light-exposed cells, 1656 of these initial candidate genes were upregulated and 811 genes were downregulated in light-exposed ARPE-19 cells (Figure 3a and Supplementary Table 1). We then used gene annotation analysis of gene ontology (GO) terms to identify pathways and processes of these regulated genes that had significant enrichment (Figure 3b). We found that, in light-damaged ARPE-19 cells, GO terms were highly enriched in pathways associated with apoptosis, cell cycle arrest, and DNA repair (Figure 3b). Among these, we identified the p53 hypoxia pathway, which controls the cell cycle when there is DNA damage, ultimately leading to cell apoptosis if the damage cannot be repaired. In the p53 signaling pathway, one of the gene with maximal change was GADD45*α*, with an eightfold high change than controls (Figure 3c). GADD45*α* has been identified as a regulator of apoptosis and cell cycle arrest.^19,20^ This protein participates in the p53 hypoxia pathway as well as G2/M checkpoint to regulate the cell cycle and cell growth in response to various environmental stresses.^19^ We used quantitative reverse transcription-PCR (qRT-PCR) to confirm that changes to GADD45*α* and found it to be markedly upregulated in light-damaged ARPE-19 cells (Figure 3d). Notably, we also found that the expression of transcription factor p53 was significantly higher in light-damaged ARPE-19 cells than in non-light controls (Figure 3d). In addition, the expression of activated p53 (phosphorylated p53, p-p53) was upregulated after light exposure (Figure 3e and Supplementary Figures 1 and 2).

### GADD45*α* upregulation is a key process in light-damaged ARPE-19 cells

**Figure 4:**
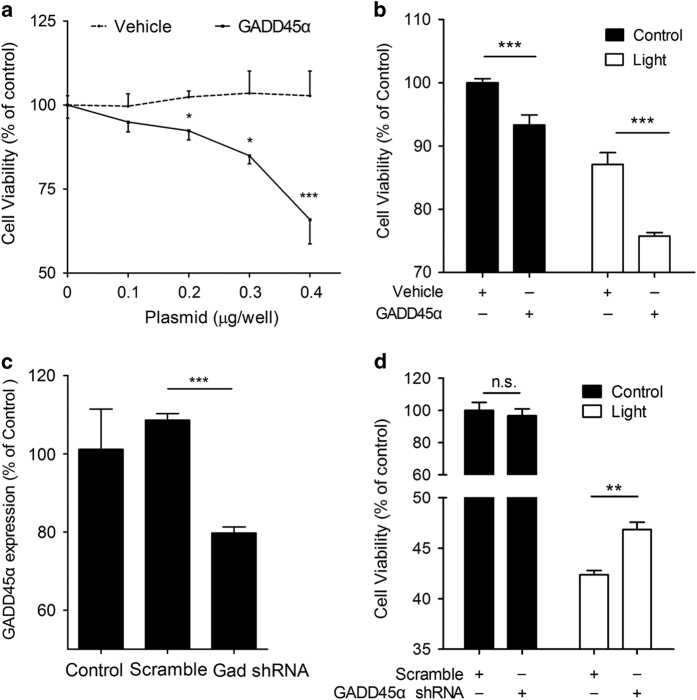
GADD45*α* stimulates apoptosis in ARPE-19 cells. (**a**) Overexpression of GADD45*α* in ARPE-19 cells resulted in a dose-dependent decrease in cell viability. Each point represents the mean value±S.E.M. (*n*=4, *P<0.05, ***P<0.001). (**b**) Overexpression of GADD45*α* significantly decreased cell viability, either with or without light damage. Each point represents the mean value±S.E.M. (*n*=4, ***P<0.001). (**c**) Knockdown of GADD45*α* in ARPE-19 cells using lentiviral delivery of shRNA. Results are given as mean value±S.E.M. (*n*=3, ***P<0.001). (**d**) Silencing GADD45*α* significantly reversed apoptosis in light-damaged RPE cells. Each value represents the mean value±S.E.M. (*n*=4, **P<0.01).

The next question we asked was whether GADD45*α* upregulation causally resulted in ARPE-19 apoptosis. To address this, we investigated the role of GADD45*α* in ARPE-19 cells. First, overexpression of GADD45*α* led to a significant dose-dependent decline of cell viability when compared with vehicle control (Figure 4a). Furthermore, forced expression of GADD45*α* markedly exacerbated cell death in light-exposed ARPE-19 cells (Figure 4b). To further elucidate the biological effect of GADD45*α* in light-induced apoptosis, we silenced GADD45*α* in ARPE-19 cells using lentiviral delivery of short hairpin RNAs (shRNAs; Figure 4c). Knockdown of GADD45*α* significantly reversed the previously observed cell viability reduction in light-damaged ARPE-19 cells (Figure 4d). Since the decrease in cell viability was primarily caused by apoptosis, these data strongly suggest that GADD45*α* is an important regulator of light-induced apoptosis of RPE cells *in vitro*.

### Dephosphorylation of AKT in light-damaged RPE cells

**Figure 5:**
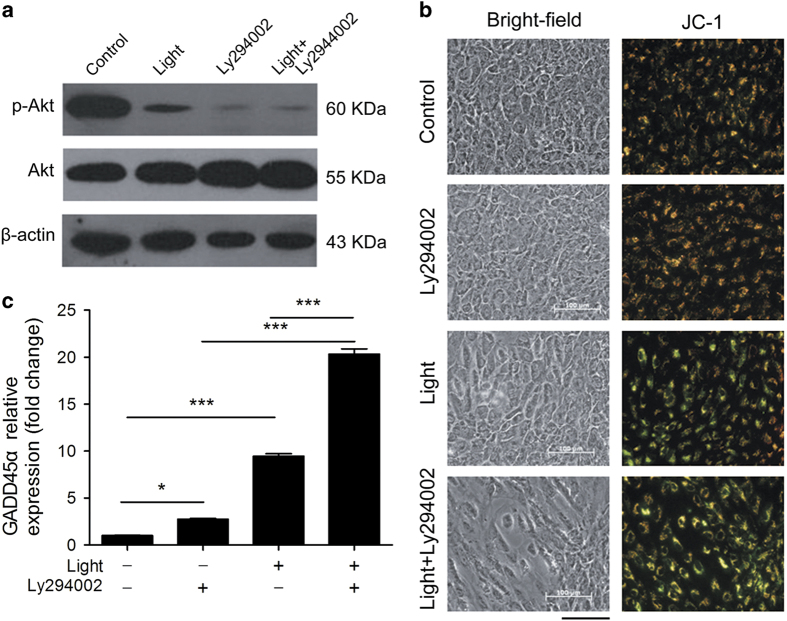
AKT dephosphorylation exacerbates apoptosis and enhances GADD45*α* expression. (**a**) Western blotting demonstrated increased dephosphorylation of AKT after light exposure and Ly294002 treatment of RPE cells after 4.5 h of exposure to 10 000 Lux white light. The *β*-actin loading control is from the same experiment as shown in Figure 3e and is reproduced in this figure for ease of reference. (**b**) ARPE-19 cells treated with Ly294002 or exposed to 10 000 Lux white light for 4.5 h showed significant changes in mitochondrial membrane potential, as measured by JC-1. Bar=100 *μ*m. (**c**) RT-PCR showed that GADD45*α* expression was significantly upregulated after 4.5 h of light exposure and/or Ly294002 treatment. Results are given as mean value±S.E.M. (*n*=3, ***P<0.001, *P<0.05).

As shown in the GO term analysis presented in Figure 3b, the PI3K-AKT network was downregulated in light-damaged ARPE-19 cells. Phosphorylated AKT (p-AKT) levels (Figure 5a and Supplementary Figures 3 and 4) and cell viability were also remarkably decreased in light-damaged ARPE-19 cells (Figure 2b). We next wanted to address whether inhibition of AKT phosphorylation aggravated RPE cellular apoptosis. To do so, we used the AKT phosphorylation inhibitor Ly294002. ARPE-19 cells pretreated with 20 *μ*M Ly294002 followed by exposure to light displayed higher levels of AKT dephosphorylation (Figure 5a) and exacerbated apoptosis (Figure 5b). Surprisingly, higher expression of GADD45*α* was also observed after Ly294002 treatment, either with or without light exposure (Figure 5c). Collectively, these findings support the idea that AKT activation plays a key role in light-induced apoptosis in ARPE-19 cells. Furthermore, that the PI3K-AKT pathway might play an important role in the upregulation of GADD45*α*.

## Discussion

In the present study, we have shown that *in vitro* exposure of human RPE cells to 10 000 Lux of blue-rich LED light led to apoptosis via upregulation of GADD45*α*. Both GADD45*α* overexpression and gene silencing results suggest that GADD45*α* serves as a crucial molecule in light-induced RPE apoptosis. Further investigation indicated that GADD45*α* expression is regulated directly by p53 signaling and is likely indirectly affected by the PI3K-AKT pathway.

### Blue light damages RPE

Thus far, great strides have been made to develop *in vitro* systems that mimic light damage in retinal cells.^21,22^ Early attempts by Noell^23^ using fluorescent lamps from General Electric Circline demonstrated uneven and unstable illumination of RPE cells. To overcome these challenges, we developed an experimental instrument for this study that we validated in terms of its homothermal and adjustable illumination. These properties enabled us to allow for a stable system for use in subsequent experiments (Figures 1a–d).

Optical radiation includes UV, visible light, and infrared radiation. Most ultraviolet radiation is absorbed by the cornea and blocked by the lens.^24–26^ However, the maximally transmitted radiation through transparent media is visible light. Mounting evidence suggests that excessive light exposure might be a significant risk factor in age-related diseases, such as age-related macular degeneration,^27,28^ itself the global leading cause of blindness. Previous studies have shown that the blue region of the spectrum is the most damaging to RPE cells.^12,14,29^ Thus, we investigated how blue-rich LED light, which has a shorter wavelength peak at 470 nm (Figure 1e), damages RPE cells.

### Light damage leads to GADD45*α* upregulation

In the present study, we found that exposure of RPE cells to intense blue-rich LED light led to an upregulation of GADD45*α*. GADD45*α* is a member of the growth arrest and DNA damage (GADD)-inducible proteins whose transcript levels are increased following cellular stress and DNA damage.^20^ Previous work has reported that mitochondrial DNA lesions occurred in light-exposed RPE cells through the actions of reactive oxygen species.^14^ GADD45*α* has been found to directly inhibit the activity of Cdc2/Cyclin B1 complex, resulting in G2 delay after UV radiation.^30^ We suspect that in light-damaged RPE cells, GADD45*α* was produced to arrest cells in G2/M in order for (i) DNA repair to occur or (ii) to overcome light-induced stresses. Notably, silencing GADD45*α* significantly reversed light-induced apoptosis, strongly suggesting its role as a therapeutic or preventative target and warranting further investigation *in vivo*. Collectively, our findings confirm the crucial role GADD45*α* plays in light-induced RPE apoptosis. To the best of our knowledge, this is the first report showing that GADD45*α* is upregulated in light-damaged RPE cells.

### Regulation of GADD45*α* in light-damaged RPE

It is generally accepted that the transcription factor p53 regulates GADD45*α* expression in many stressed cell types.^31–33^ In the present study, qRT-PCR and western blotting demonstrated that both the transcription of p53 and its activated form (p-p53) were increased in light-damaged ARPE-19 cells. These data indicate that p53 directly regulates GADD45*α* expression.

As previous studies have shown, the PI3K-AKT pathway contributes to improved cell survival in response to light exposure.^34,35^ Light-induced AKT inhibition can be stimulated by PI3K-dependent and -independent mechanisms.^36,37^ Ly294002, a PI3K inhibitor, has been commonly used to block AKT activation.^38,39^ Our study showed that Ly294002 markedly inhibited AKT activation and exacerbated light-induced apoptosis in RPE cells (Figure 5b). These findings strongly suggest that light exposure triggers the AKT survival pathway in RPE cells via a PI3K-dependent pathway. Notably, we discovered that the inhibition of AKT phosphorylation promotes GADD45*α* transcription (Figure 5c), supporting the idea that PI3K-AKT signaling might participate in the regulation of GADD45*α* expression.

Given this, it should be noted that we cannot conclusively elucidate the relationship between GADD45*α* and AKT phosphorylation. However, this is not the first report showing that GADD45*α* expression might be regulated through AKT levels. For instance, in Hepatitis C-infected cells, p53 expression was downregulated by increased AKT phosphorylation. This ultimately led to a decline in GADD45*α* expression.^40^ Thus, it is plausible that the expression of GADD45*α* might be mediated by p53 via PI3K-AKT signaling.

## Conclusion

In summary, we have shown that blue-rich light exposure of RPE cells induces significant cell apoptosis through GADD45*α* upregulation, itself mediated via p53 and AKT dephosphorylation. Regulation of GADD45*α* significantly alters light-induced apoptosis and may be a potential target for therapeutic purposes, opening up a novel avenue for light-damage-related retinal disease prevention and treatment.

## Materials and Methods

### Development of a quantitative instrument, SmartLight2.0

We built an experimental instrument, *SmartLight2.0*, to model light damage on retinal cells without the confounding effect of thermal damage. *SmartLight2.0* is a balanced, adjustable LED system that is homothermal, which is achieved through utilization of an automatic cooling fan for thermostatic protection. *SmartLight2.0* is composed of the following parts: a display control screen, a camera obscura, an LED light, a semiconductor chilling unit, a light-to-digital converter, a temperature sensor, light-intensity sensors, and a feedback cooling fan system. The cell culture dish used in each study is placed in the center of the top of the platform. Differences in temperature were measured between the measured data and a previously applied setting. Furthermore, temperature controlling system was tested. To assess the action spectrum of the LED light, a spectrometer (Maya2000 Pro, Ocean Optics, Dunedin, FL, USA) was used.

### Cell culture

Human RPE cells (ARPE-19, ATCC No: CRL-2302) obtained from ATCC in Chicago, IL, USA, are an immortalized cell line obtained from a 19-year-old donor, which were seeded in 12-well plates and T25 flasks. Cells were cultured in Dulbecco’s modified Eagle’s medium (DMEM, Gibco, Grand Island, NY, USA) supplemented with 10% fetal bovine serum (Gibco) and 100 U/ml Penicillin-Streptomycin (Gibco)^41^ before being incubated in a humidified 5% CO_2_ chamber at 37 °C. After reaching 90% confluence, medium was replaced with DMEM with 1 g/l glucose and pyruvate, 1% FBS and 100 U/ml Penicillin-Streptomycin, and then cultured for 2 months before light exposure.^42^

As a control, primary human fibroblasts were obtained from normal skin following nevus resection (with informed consent and approval from the Ethics Committee of our institute) and then purified according to the standard procedures.^43^ In brief, dermal tissue was cut into 3 mm^2^ pieces and dermal explants were plated in 25 cm^2^ flasks. They were subsequently incubated with DMEM medium containing 20% FBS and 100 U/ml Penicillin-Streptomycin at 37 °C and in a humidified 5% CO_2_ chamber to allow fibroblast proliferation. After one week, the dermal clumps were removed and the fibroblasts were incubated with 10% FBS/DMEM medium until 90% confluence. Cells were then split and seeded into 10 cm dishes (2.5×10^6^ cells/dish).^44,45^

### Light exposure

Before light exposure, cells were washed three times with phosphate-buffered saline and the medium was replaced with modified DMEM (without folate photosensitive material, phenol red, riboflavin, l-tryptophan, or l-tytosine) (Gibco). ARPE-19 and primary human fibroblasts were then subjected to blue-rich LED light using our *SmartLight2.0* to examine the resulting light damage.

### Cell viability assay

The viability of cells cultured in 96-well plates was then measured using the WST-1 cell proliferation and cytotoxicity assay kit (Roche, Mannheim, Germany) and according to the manufacturer’s instructions. Absorbance at the experimental wavelength of 440 nm and the reference wavelength of ~630 nm were monitored using Spectra Max M5 (Molecular Devices, Sunnyvale, CA, USA).

### Mitochondrial membrane potential assay (∆ψ*m*)

∆ψ*m* was analyzed using a JC-1 assay kit (Beyotime, Nantong, Jiangsu, China) and according to the manufacturer’s instructions. Briefly, cells were cultured and treated on coverslips in 24-well plates, followed by incubation with an equal volume of JC-1 staining solution at 37 °C for 20 min. After two rinses with phosphate-buffered saline, the ∆ψ*m* were monitored by determining the relative amount of dual emissions from mitochondrial JC-1 monomers (green fluorescence) or aggregates (red fluorescence) using a fluorescent microscope (Zeiss, Gottingen, Germany).

### Annexin-V/PI double staining

ARPE-19 cellular apoptosis and necrosis were analyzed using an Annexin-V-FLOUS staining kit (Roche). In brief, coverslips containing ARPE-19 cells were incubated in Annexin-V-FLOUS and PI labeling solution for 15 min at room temperature and observed under a confocal microscope (Zeiss). Three populations of cells were predominantly visible: viable cells were both Annexin-V and PI negative, cells undergoing apoptosis were Annexin-V single positive, and late-stage apoptosis or necrotic cells were either Annexin-V and PI positive or PI single positive.

### RNA isolation

Total RNA was extracted using TRIzol reagent (Invitrogen, San Diego, CA, USA), following the manufacturer’s instructions. Quantification of RNA yield and purity were assessed using a NanoDrop ND-1000 (Thermo Fisher Scientific Inc., USA). The RNA integrity number (RIN) value was ascertained using an Agilent RNA6000 Nano assay (Agilent technologies, Germany) and used as a measure of RNA integrity.

### Gene expression microarray and qRT-PCR

For hybridization, 10 *μ*g of Cy5-labeled aRNA was utilized in the Phalanx Hybridization Protocol Array Version HOA6.1. qRT-PCR was also employed to corroborate putative results. qRT-PCR was carried out in 20 *μ*l reaction volumes using SYBR Green PCR mix (Roche) and run on an Applied Biosystem's 7500 instrument, Singapore. The relative differences (*n*-fold) in gene expression were normalized to the level of the housekeeping gene *β*-actin as well as designated controls as a reference. Primer sequences were obtained from the Primer Bank database (http://pga.mgh.harvard.edu/primerbank/) and Primer Premier 5 (Table 2).

### Immunobloting analysis

Protein extraction was performed using cell lysis buffer and 1 mM phenylmethanesulfonyl-fluoride (Beyotime). The supernatant was collected and quantified using a BCA reagent (Beyotime) and according to the manufacturer’s instructions. Equal amounts of protein were separated using 10% SDS-PAGE. Proteins were subsequently transferred to PVDF membranes and blocked in 5% bovine serum albumin at room temperature for 1 h. Targeted proteins were probed with the following primary antibodies: rabbit monoclonal antibody to phospho-AKT (1 : 2000), AKT (1 : 1000), and phospho-p53 (1 : 1000). All antibodies were obtained from Cell Signaling Technology (Frankfurt, Germany). Membranes were incubated with a given primary antibody overnight at 4 °C, then with appropriate HRP-conjugated secondary antibody for 1 h at room temperature. Resulting immunocomplexes were visualized using a chemiluminescent autography detection system (Beyotime). Protein levels were normalized by probing the membranes for *β*-actin (1 : 1000; KangChen, Shanhai, China).

### Construction of pLL3.7/shRNA plasmid and lentivirus packaging

Three pairs of shRNA oligonucleotides against human GADD45*α* were designed. An *Hpa*I site was introduced into the 5′ end and an *Xho*I site was introduced into the 3′ end of the shRNA sequence. The shRNA sequences used to target GADD45*α* were as follow: 5′-TAACGTCGACCCCGATAACGTG-TTCAAGAGACACGTTATCGGGGTCGACGTTTTTTTC-3′; 5′-TAACATCCTGCGCGTCAGCAACTTCAAGAGAGTTGCTGACGCGCAGGATGTTTTTTTTC-3′; 5′-TAAAGTCGCTACATGGATCAATTTCAAGAGAATTGATCCATGTAGCGACTTTTTTTTTC-3′. BLAST searches were performed using the National Center for Biotechnology Information (NCBI) database to ensure that the shRNA constructs were targeting only human GADD45*α*. Oligos were annealed and cloned into the *Xho*I site of the pLL3.7 vector. HEK293/17 cells were planted on 10 cm dishes in DMEM containing 10% fetal bovine serum and transfected with 4 μg of pLL3.7/shRNA or pLL3.7, 4 *μ*g of the packaging plasmid pSpAX2, and 2 *μ*g of the envelope plasmid pMD2.G using the calcium-phosphate method. Infectious lentiviruses were harvested at 48 h post transfection, filtered through 0.2 *μ*m syringe filters, and used to transfect ARPE-19 cells and primary human fibroblast controls.

### GADD45*α* overexpression

The gene encoding GADD45*α* was amplified by PCR from cDNA obtained from ARPE-19 cells using the following forward primer containing an *Hin*dIII site: 5′-GCAAGCTTATGACTTTGGAGGAATTCTCGGCTGG-3′ and the following reverse primer containing a *Xho*I site: 5′-CCTCGAGTCACCG TTCAGGGAGATTAATCACTG-3′. The final PCR product was cloned into the corresponding sites within the pCMV-tag2b vector. The recombinant plasmid was transfected into the ARPE-19 cells and control (primary human fibroblasts) using Lipofectamine 2000 (Invitrogen) and according to the manufacturer’s protocols.

### Ly294002 treatment

ARPE-19 cells pretreated with 20 *μ*M Ly294002 (Selleckchem, Burlington, NC, USA) for 6 h before light exposure.

### Statistical analysis

Unpaired *t*-tests and analysis of variance were used to determine differences using Graphpad Prism (California Corporation, San Diego, CA, USA). Error bars in all graphical figures represent the standard error of the mean (S.E.M.). Differences were considered to be statistically significant at *P*<0.05.

## Figures and Tables

**Table 1:** Parameters of *SmartLight2.0* lighting system

*Parameter*	*Range*
Light intensity range	0–12 000 Lux
Temperature adjustment range	20−40 °C
Temperature error range^a^	<37±0.4 °C
Light error range^a^	<10 000±420 Lux

a The data represent mean value±S.E. of three detection.

**Table 2:** Primer set for qRT-PCR

*Gene*	*Primer sequences (5′→3′)*	*Product size (bp)*	*Annealing temperatures (*°C*)*
*GADD45α*	GAGAGCAGAAGACCGAAAGGA	145	61.2
	CACAACACCACGTTATCGGG		60.5
*p53*	CAGCACATGACGGAGGTTGT	125	59.6
	TCATCCAAATACTCCACACGC		61.0
*AKT1*	AGCGACGTGGCTATTGTGAAG	96	62.7
	GCCATCATTCTTGAGGAGGAAGT		61.9
*HIF1α*	GGCGCGAACGACAAGAAAAAG	154	59.8
	CCTTATCAAGATGCGAACTCACA		60.4
*β-Actin*	TCCCTGGAGAAGAGCTACGA	194	60.2
	AGCACTGTGTTGGCGTACAG		60.5

## Abbreviations

GADD45*α*: growth arrest and DNA damage-45 alpha
RPE: retinal pigmented epithelium
LEDs: light-emitting diodes
PI3K: phosphatidylinositol 3-kinase
PI: propidium iodide
GO: Gene ontology
qRT-PCR: quantitative reverse transcription-PCR
shRNA: short hairpin RNA
DMEM: Dulbecco’s modified Eagle’s medium
FBS: fetal bovine serum.
