# Supplementary figures and images for: Upregulation of GADD45α in light-damaged retinal pigment epithelial cells
Source: Cell Death Discov. 2016 Feb 29;2:16013–. doi: 10.1038/cddiscovery.2016.13 (PMC4979445; doi:10.1038/cddiscovery.2016.13)

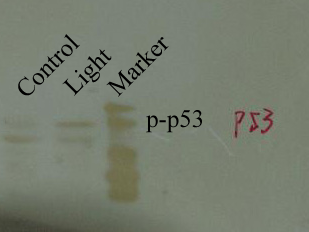

Supplement: Supplementary Figure S1 [file cddiscovery201613-s1.tiff]

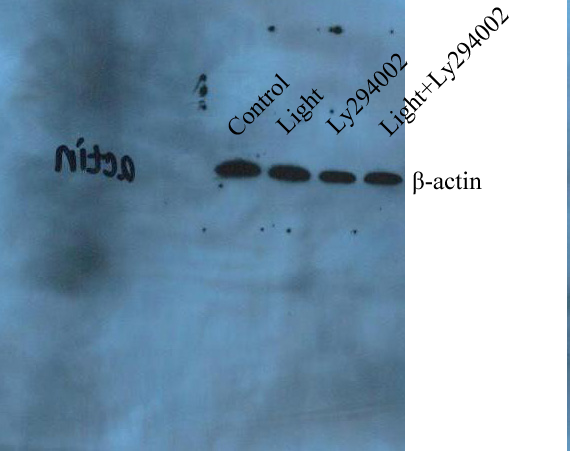

Supplement: Supplementary Figure S2 [file cddiscovery201613-s2.tiff]

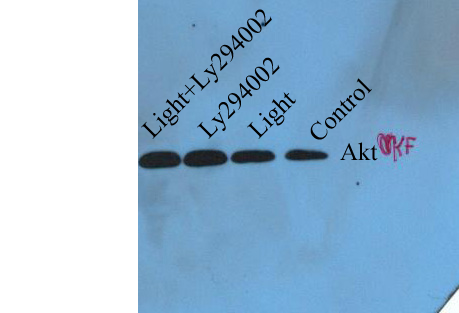

Supplement: Supplementary Figure S3 [file cddiscovery201613-s3.tiff]

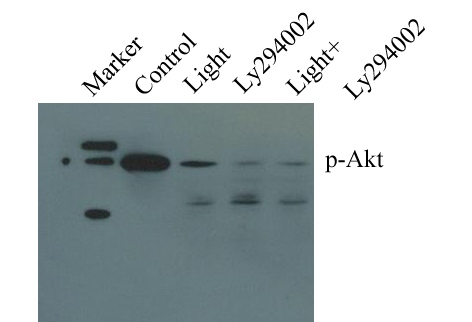

Supplement: Supplementary Figure S4 [file cddiscovery201613-s4.tiff]
