# Supplementary material for: Upregulation of GADD45α in light-damaged retinal pigment epithelial cells
Source: Cell Death Discov. 2016 Feb 29;2:16013–. doi: 10.1038/cddiscovery.2016.13 (PMC4979445; doi:10.1038/cddiscovery.2016.13)
Supplement: Supplementary Table 1 [file cddiscovery201613-s5.pdf]

P-value (Detected)		Normalized Intensity		log2 (Ratio) non-light-damaged/light-damaged	P-value (Differentially expressed) non-light-damaged/light-damaged	
light-damaged	non-light-damaged	light-damaged	non-light-damaged			Gene_symbol
0.000123	0.	819.620911	24164.8262	4.8628164	0.000001	ATF3
0.115426	0.000001	233.72821	6464.28711	4.7667615	0.000002	PSMC2
0.97525	0.000004	89.089226	2001.14673	4.4986683	0.000004	HSPA6|HSPA7
0.000044	0.	987.318298	22230.6074	4.4845416	0.000002	HMOX1
0.926103	0.000004	103.762749	1951.42883	4.2410273	0.000005	previous version conserved probe
0.979535	0.000009	86.993012	1487.7135	4.0960575	0.000006	TEX36
0.064798	0.000001	318.625	5642.02881	4.093883	0.000004	HBEGF
0.000003	0.000004	2169.58472	36690.875	4.0726096	0.000023	SLC3A2
0.050978	0.000001	273.556335	4755.71143	4.0644155	0.000004	CCT2
0.00073	0.000001	564.930542	9458.83594	4.0187479	0.000003	PSMD4
0.456661	0.000002	170.841705	2712.49561	3.9705541	0.000007	COPE
0.062996	0.000001	263.075256	4206.90186	3.9383866	0.000004	previous version conserved probe
0.001534	0.	713.761963	10836.5967	3.8852907	0.000004	HSPA8
0.967932	0.000014	90.137337	1301.27124	3.8520208	0.00001	IL11
0.284649	0.000002	189.707657	2764.12573	3.8368946	0.000006	NA
0.240422	0.000002	201.236847	2870.25439	3.8004704	0.000006	ARNTL
0.000004	0.	2071.0625	28243.6094	3.7673625	0.000004	SLC25A25
0.94912	0.000014	97.474098	1323.26184	3.7632579	0.00001	FDPS
0.000453	0.000001	613.143494	8401.37402	3.7273846	0.000007	PHB2
0.994745	0.000042	74.41571	984.797485	3.7238412	0.000015	HNRNPA0
0.328305	0.000003	182.370895	2401.75854	3.6892458	0.000008	UQCRC1
0.308997	0.000003	185.515213	2430.44189	3.6807919	0.000008	CDKN1A
0.143593	0.000002	223.247131	2930.4895	3.6710169	0.000007	PRDX3
0.122377	0.000002	250.497955	3272.77832	3.6529413	0.000007	FOSB
0.053892	0.000001	271.460114	3431.49341	3.5988399	0.000007	CYTH2
0.370047	0.000004	176.082245	2126.39771	3.5607395	0.00001	LOC344887
0.003159	0.000001	444.398041	5472.79688	3.5542185	0.000007	SUCLG1
0.009738	0.000001	363.693665	4491.82373	3.552669	0.000007	IFITM10
0.000685	0.000001	569.122986	6928.00244	3.5521918	0.000006	LUZP1
0.225481	0.000002	240.016861	2903.71826	3.5466885	0.00001	AHSA1
0.855046	0.000013	115.291946	1328.04248	3.5178684	0.000014	SRP68
0.113474	0.000002	234.776321	2728.74951	3.491304	0.000009	AIP
0.377663	0.000001	426.5802	5016.73047	3.4819881	0.000777	EIF4G2
0.060379	0.000002	265.171478	3082.51172	3.4798393	0.000009	TRA2B
0.013365	0.000001	351.116364	4074.00195	3.4607072	0.000008	XBP1
0.893581	0.000019	110.051399	1198.96704	3.4418506	0.000018	MPDU1
0.000122	0.000001	790.273865	8743.66309	3.4278352	0.000007	MCL1
0.887535	0.000063	111.09951	1196.09863	3.4238914	0.000064	UCP3
0.046316	0.000002	278.796875	3093.98511	3.4102716	0.00001	HIST1H4H

0.947832	0.000033	98.522209	1045.03271	3.4072366	0.000021	ZNF655
0.925549	0.000028	103.762749	1089.01392	3.3925132	0.000021	RHBDD2
0.990279	0.000086	79.65625	839.46814	3.3883484	0.000028	MAX
0.983225	0.000066	84.896797	889.186096	3.3819442	0.000027	ARPC1B
0.98842	0.000101	79.65625	816.521423	3.3471552	0.000033	NFIL3
0.000024	0.	1152.91943	11870.1562	3.3466328	0.000008	ATP6V1E1
0.232784	0.000004	201.236847	2111.09985	3.3450152	0.000014	NR1D2
0.000005	0.	1945.28955	19445.4473	3.3270964	0.000008	DKK1
0.559364	0.000008	151.975739	1553.6853	3.3209436	0.000017	CHMP4B
0.864725	0.000026	112.147621	1110.04846	3.3020063	0.000025	CRK
0.296534	0.000004	199.140625	2015.4884	3.2916777	0.000018	CAPNS1
0.824921	0.000021	120.532486	1178.88867	3.2782712	0.000023	CCDC147
0.362914	0.000006	177.130356	1761.16211	3.2694163	0.000017	C1orf63
0.000205	0.000001	711.66571	7068.55127	3.2630208	0.00001	PSAT1
0.044483	0.000001	613.143494	6093.31494	3.2531203	0.000016	CCT7
0.117953	0.000003	232.680099	2301.36646	3.2514517	0.000015	RAF1
0.000009	0.	1529.19043	14610.3789	3.2498828	0.000009	DUSP8|GLUD1P3
0.747525	0.000046	101.666534	965.675171	3.2445318	0.000093	ARL4D
0.04689	0.000002	277.748779	2724.92505	3.2334717	0.000014	FAM53C
0.000126	0.000001	785.033325	7603.9751	3.2326546	0.000011	LMAN2
0.012701	0.000001	704.328979	6836.21533	3.2285837	0.000013	SNRPB
0.002207	0.000001	465.360199	4583.61084	3.220838	0.000012	BCAP31
0.964829	0.000071	93.281662	873.888245	3.2202676	0.000035	PDE4DIP|LOC101060353|LOC101060456|LOC100996723|LOC100996761
0.00002	0.	1209.51733	11372.0205	3.2130692	0.00001	HNRNPA1P10|HNRNPA1L2
0.910737	0.000042	106.907074	987.665833	3.2037579	0.000032	ASNA1
0.980607	0.000125	83.848686	776.364624	3.1982	0.000044	SLC30A1
0.550335	0.000011	153.023849	1412.18042	3.1729119	0.000024	LAT2
0.669961	0.000035	114.243835	1028.77869	3.1627659	0.000133	ETNK1
0.063019	0.000003	263.075256	2449.56421	3.1565013	0.000017	CHMP5
0.62356	0.000014	144.638977	1311.78845	3.1529885	0.000026	previous version conserved probe
0.007673	0.000001	378.367188	3530.9292	3.1434927	0.000015	AKAP8L
0.000461	0.000001	611.047302	5610.47705	3.1336784	0.000014	RAB9A
0.049865	0.000002	276.700653	2535.6145	3.1326816	0.000018	EDN1
0.267468	0.000006	192.851974	1734.39087	3.1159833	0.000023	TRUB2
0.09908	0.000003	241.064972	2172.29102	3.1106875	0.00002	previous version conserved probe
0.10128	0.000003	240.016861	2137.87109	3.0934558	0.000021	PFKL
0.684808	0.000025	131.013565	1126.30237	3.0849203	0.000044	NASP
0.793122	0.003378	124.724922	1067.97937	3.0836552	0.005101	previous version conserved probe

0.245967	0.000006	197.044418	1709.53198	3.0613616	0.000026	YARS
0.128716	0.000004	228.487671	1981.06836	3.0549247	0.000024	MNAT1
0.868152	0.000049	114.243835	952.289612	3.0479374	0.000044	SGK1
0.000362	0.000001	646.682983	5590.39893	3.0467632	0.000016	MAFK
0.56844	0.000016	150.927643	1274.5	3.0463124	0.000033	SAMD4A
0.000045	0.000001	984.17395	8308.63086	3.0461154	0.000015	MAP1LC3B
0.000208	0.000001	711.66571	6040.72852	3.0286272	0.000017	ARF1
0.000001	0.	3795.20117	30743.8477	3.0242274	0.000014	GADD45A
0.000004	0.	2092.02466	16842.9043	3.0232325	0.000015	previous version conserved probe
0.000001	0.	5968.97852	47494.0078	3.018381	0.000014	GAPDH
0.977884	0.000713	84.896797	695.09491	3.0159331	0.000311	previous version conserved probe
0.51472	0.000015	157.216293	1296.4906	3.0084557	0.000035	LATS2
0.954925	0.000121	96.425987	781.145203	3.0056898	0.00006	CHIC2
0.877782	0.000418	76.511925	615.737427	2.9890234	0.000362	ENDOV
0.000606	0.000001	581.700256	4836.0249	2.9806893	0.000019	CLU
0.015245	0.000002	337.490967	2794.72144	2.9785617	0.000023	EIF3M
0.893415	0.000297	82.800575	655.894226	2.966505	0.000203	RPL6
0.16719	0.000006	215.91037	1761.16211	2.9656078	0.000031	TMEM55B
0.938733	0.000115	100.618423	789.750183	2.9605728	0.000065	NFX1
0.580402	0.000021	148.831421	1177.9325	2.9542621	0.000045	TACO1
0.096948	0.000004	242.113083	1963.85828	2.9529727	0.00003	METTL17
0.064355	0.000004	262.02713	2051.8208	2.8972925	0.000032	CHCHD7
0.651292	0.000031	141.494659	1070.84778	2.8944036	0.000054	ZHX2
0.99402	0.010791	75.463821	564.1073	2.882175	0.005661	AKT3
0.176574	0.000004	258.882812	1998.27832	2.8762217	0.000051	ZNF451
0.642019	0.000031	142.54277	1059.37439	2.867544	0.000057	ATF1
0.954432	0.000195	96.425987	710.392761	2.8645769	0.00009	ATXN7L3
0.704504	0.000078	116.34005	857.634277	2.8645019	0.000196	SMPDL3A
0.970362	0.000262	91.185448	672.148193	2.8629454	0.000097	ING3
0.001382	0.000001	504.140228	3882.77905	2.8616358	0.000027	TMCO1
0.1097	0.000004	269.363892	2043.2157	2.8495216	0.000043	NOP56
0.997358	0.001314	69.175163	504.828217	2.8470187	0.000155	PPP2R2A
0.532413	0.000023	155.120071	1138.73181	2.8421724	0.000056	TGFB2
0.321764	0.000013	183.419006	1355.76978	2.8364879	0.000049	CCDC101
0.01495	0.000002	343.779602	2583.42017	2.8361674	0.000033	RPS6KB2
0.000655	0.	1564.82605	11195.1396	2.8172888	0.000028	NBPF14|LOC101060684|LOC101060362
0.000064	0.000002	907.662048	6570.41602	2.8148972	0.000064	SFPQ
0.973151	0.000374	89.089226	631.991394	2.8071662	0.000125	FAM134B
0.035553	0.000002	350.068268	2574.81519	2.804073	0.000041	previous version conserved probe
0.000021	0.000001	1197.98816	8524.71289	2.8024697	0.000026	IMPDH2
0.001494	0.000003	513.573181	3790.99219	2.8006164	0.000055	IDH3B

0.020596	0.000003	320.721222	2352.99658	2.8000492	0.000038	previous version conserved probe
0.898248	0.000175	103.762749	725.690552	2.7904488	0.000117	ACVR1
0.348669	0.000015	179.226562	1275.45618	2.7840379	0.000058	ACBD7
0.000171	0.000001	737.868408	5290.1792	2.7781756	0.00003	FILIP1L
0.998059	0.002104	67.078949	465.627533	2.7744253	0.000202	ZNF462
0.012312	0.000003	383.607727	2765.08179	2.7717816	0.000047	MT1M|MT1JP
0.750771	0.000062	129.965469	902.571655	2.7708678	0.000083	ARMCX6
0.012696	0.000003	347.972046	2459.12524	2.7442072	0.000042	NUDT9
0.031304	0.000003	306.047699	2160.81787	2.7424631	0.000046	IDI1
0.994021	0.001315	75.463821	503.872101	2.7187442	0.0002	BCR
0.007934	0.000002	376.270966	2606.36694	2.7144096	0.000044	IKBIP
0.977831	0.000867	81.752472	541.160583	2.7065938	0.000214	ABR
0.614327	0.000045	145.687088	968.543518	2.7009118	0.000091	IGBP1
0.056655	0.000005	268.315796	1841.47571	2.6996707	0.000055	SMN2|SMN1
0.471677	0.000029	162.456833	1077.54053	2.6918933	0.000084	CNPY3
0.000848	0.000004	548.160767	3732.66919	2.6846185	0.000103	TAB2
0.75944	0.000084	128.917358	844.248718	2.6839774	0.000109	PRPF18
0.000005	0.	2079.44751	13289.9854	2.6764759	0.000034	MDH1
0.48893	0.000032	160.360611	1050.76929	2.6751184	0.000091	PLD3
0.16358	0.00001	216.958481	1448.51282	2.6740654	0.000069	TEX10
0.992323	0.001373	77.560036	501.003754	2.6709536	0.000228	FIP1L1
0.001541	0.000001	494.707245	3329.18921	2.6678727	0.000045	REV3L
0.539875	0.000039	154.07196	1001.05139	2.6643158	0.000099	RBM34
0.951601	0.000385	97.474098	625.298584	2.6619896	0.000166	RTCA
0.004516	0.	1739.86023	11052.6787	2.6474785	0.000056	GTF2H1
0.977488	0.000717	88.041122	559.326721	2.647465	0.000201	OR4S2
0.081524	0.000007	250.497955	1652.16504	2.6429086	0.000069	NFX1
0.026016	0.000004	308.143921	2031.74231	2.6400223	0.000061	IP6K2
0.937907	0.000354	100.618423	634.859741	2.6381484	0.000174	YKT6
0.893566	0.00022	110.051399	694.138794	2.6352096	0.000154	NOTCH1
0.623088	0.000059	144.638977	913.088928	2.6243324	0.000116	GFOD1
0.528911	0.000069	140.446548	881.53717	2.6170926	0.021603	ORC5
0.951342	0.00046	97.474098	605.220215	2.6147546	0.000194	NXN
0.961746	0.000571	94.329773	582.273438	2.6061195	0.000209	ZNF7
0.000494	0.000001	603.710571	3873.21802	2.5988911	0.000052	EXPH5
0.847234	0.000178	117.388161	723.77832	2.5985598	0.00016	NBPF1|LOC101059961|NBPF12|LOC101060238|NBPF14|LOC101060684|LOC101060298|LOC101060362|NBPF10|LOC101060692
0.000803	0.000001	553.401306	3532.84131	2.5919117	0.000054	TIMM23B|TIMM23
0.951608	0.000531	97.474098	589.922363	2.5777022	0.000219	LCOR
0.000057	0.000001	931.768555	5739.55273	2.5745585	0.00005	EMC3
0.128728	0.000011	228.487671	1426.52222	2.5725831	0.00009	THUMPD2

0.000051	0.000001	956.923157	5873.40869	2.5720178	0.00005	LRPAP1
0.994745	0.002906	74.41571	448.41748	2.5701712	0.000391	ILVBL
0.000164	0.000001	744.157104	4622.81152	2.5631595	0.000054	EIF1B
0.057856	0.000007	267.2677	1673.19958	2.5622763	0.000085	MOB2
0.073192	0.000008	255.738495	1592.88599	2.5585204	0.000087	previous version conserved probe
0.007935	0.000003	376.270966	2344.3916	2.5557749	0.00007	NGF
0.651291	0.000305	141.494659	851.897644	2.5548785	0.000551	LOC101060399|CDR2
0.80929	0.000165	122.6287	734.295593	2.5531434	0.000176	GET4
0.003557	0.000002	429.724518	2644.61157	2.5393123	0.000072	CALR
0.380998	0.000024	188.659546	1135.86353	2.5384162	0.000275	AGPAT2
0.004068	0.000002	420.291534	2581.50806	2.5350662	0.000071	SOAT1
0.013135	0.000004	346.92395	2123.5293	2.5289905	0.00008	C1QBP
0.019671	0.000004	325.961761	1987.76111	2.5232173	0.000084	CGRRF1
0.000151	0.000001	760.926819	4588.39111	2.5207221	0.000062	previous version conserved probe
0.939695	0.00056	100.618423	584.185669	2.5177552	0.000257	SUPT3H
0.004207	0.000002	447.542358	2711.53955	2.5172282	0.000076	SPTBN1
0.412128	0.000039	170.841705	1002.00751	2.5074704	0.000151	ASB6
0.000177	0.000001	732.627869	4378.04639	2.5030544	0.000065	CITED2
0.001481	0.000002	497.851562	2985.94409	2.5024794	0.000074	THAP7
0.660518	0.000099	140.446548	815.565308	2.5014804	0.000178	C7orf25
0.947838	0.00069	98.522209	563.151184	2.4950557	0.000286	TMEM217
0.085114	0.00001	248.401733	1475.28394	2.4927466	0.000109	CNST
0.775928	0.000181	126.821136	720.909973	2.4743391	0.000221	AKT3
0.44468	0.000028	189.707657	1092.83838	2.47431	0.005656	ABCF2
0.080467	0.00001	251.546051	1474.32788	2.4725099	0.000116	TPRN
0.208229	0.00002	208.573608	1193.23035	2.4553754	0.000149	ATG13
0.995388	0.004877	73.3676	405.392365	2.4469557	0.00053	INO80
0.083344	0.000012	249.449844	1428.43445	2.439815	0.000141	PVR
0.000969	0.000002	535.583496	3071.99438	2.4382242	0.000088	previous version conserved probe
0.000006	0.000001	1710.51318	9420.5918	2.4356773	0.000069	ID1
0.00722	0.000003	382.559631	2198.1062	2.4345127	0.000102	RPS20
0.000991	0.000002	533.487244	3039.48657	2.4285845	0.000091	CYP1A1
0.000014	0.000001	1651.81909	9054.40039	2.4264201	0.000073	ILF2
0.000046	0.000001	975.789062	5416.38623	2.4226711	0.000079	CITED2
0.061858	0.000009	265.171478	1503.96741	2.4205004	0.000133	NACA
0.0052	0.000003	403.52179	2294.67383	2.4191522	0.000113	ERH
0.742008	0.000186	131.013565	717.08551	2.4166463	0.000258	FNBP1L
0.365885	0.000008	276.700653	1567.07092	2.4160646	0.009043	PEX13
0.000332	0.000001	649.827332	3663.8291	2.4132076	0.00009	MBD2
0.001111	0.000002	523.006165	2944.8313	2.4116804	0.000097	PHF5A
0.000077	0.000001	870.97821	4826.46387	2.4079348	0.000087	previous version conserved probe

0.644528	0.000311	120.532486	650.157532	2.3997835	0.00266	SCAF8
0.001576	0.000002	492.611023	2736.39844	2.3923514	0.000105	EPHA4
0.584401	0.000113	146.735199	792.61853	2.3920089	0.000328	FIBP
0.309342	0.000025	205.429276	1117.69739	2.3845205	0.000364	LSM1
0.817174	0.000441	121.580597	648.2453	2.3822666	0.000455	ERCC5|BIVM-ERCC5
0.000444	0.000002	615.239746	3394.20483	2.3820785	0.00011	DTD1
0.002895	0.000002	450.686676	2490.677	2.3810128	0.000114	DENND5B
0.809289	0.000304	122.6287	653.025879	2.3797607	0.000319	ILF3
0.235788	0.000029	199.140625	1077.54053	2.3795427	0.000191	FERMT2
0.001019	0.000002	531.391052	2920.92847	2.3771023	0.000108	PICALM
0.150868	0.000019	221.150909	1202.7915	2.3770144	0.000178	KLF6
0.405999	0.000056	171.889801	923.606201	2.376989	0.000237	UBAC2
0.996006	0.008877	71.271385	373.840607	2.3761085	0.000895	WDR45B
0.000354	0.000001	641.442444	3523.28027	2.3757038	0.000102	MTCH1
0.000204	0.000001	712.713806	3899.98926	2.3729924	0.000099	NOP56
0.050403	0.000009	274.604462	1507.79187	2.3719037	0.000152	PSMB8
0.114477	0.000009	277.748779	1524.04578	2.3703243	0.000219	NKIRAS1
0.497287	0.000145	159.3125	849.029297	2.3683027	0.000416	LRRC58
0.652349	0.000155	140.446548	743.85675	2.3646925	0.000304	GALNT10
0.135515	0.000009	271.460114	1479.1084	2.3614623	0.000249	PLTP
0.240811	0.000032	198.092514	1056.50598	2.359077	0.000206	PLXNB1
0.586717	0.000119	148.831421	784.01355	2.3540707	0.000278	ARL5B
0.623541	0.000138	144.638977	761.066772	2.3536606	0.000288	RBAK
0.226476	0.000007	303.951477	1654.07727	2.3534572	0.000852	AKR1A1
0.071624	0.000012	256.786591	1385.4093	2.3512925	0.000171	PP12719
0.51471	0.000092	157.216293	827.994751	2.3512923	0.000265	previous version conserved probe
0.00044	0.000002	616.287842	3315.80347	2.3460664	0.000114	TIMM23B|TIMM23
0.076399	0.000012	253.642273	1360.55029	2.344126	0.000177	CSDE1
0.000064	0.000001	906.613892	4797.78027	2.3428518	0.000104	COPS6
0.000015	0.000001	1311.18384	6807.53223	2.3426085	0.000097	TIPARP
0.000664	0.000002	572.267273	3071.03833	2.342504	0.000118	CFDP1
0.000471	0.000001	616.287842	3301.46191	2.3398408	0.000116	PROCR
0.967689	0.001908	92.233551	473.276459	2.3385756	0.000548	ATRAID
0.000033	0.000001	1060.68591	5531.11963	2.3369858	0.000102	NDUFV1
0.000027	0.000001	1112.04321	5778.75342	2.3346205	0.000102	SAP30
0.00003	0.000001	1128.81299	5855.24268	2.3327951	0.000104	PPP1R10
0.947732	0.001331	98.522209	502.915985	2.3313313	0.000513	ZBTB38
0.010083	0.000004	361.597473	1938.04321	2.3301892	0.000149	CLCF1
0.974693	0.002477	89.089226	453.198059	2.3258755	0.000609	CCNYL1
0.000013	0.000001	1378.26282	7050.38525	2.3218163	0.000103	UQCRC2
0.321757	0.000049	183.419006	951.333496	2.321329	0.000252	CASP4
0.000003	0.	2233.51929	11203.7441	2.3211845	0.000097	GRN
0.967691	0.002054	92.233551	467.539764	2.3209253	0.000585	DNAJB4
0.514718	0.000101	157.216293	810.784729	2.3200739	0.000296	POLR3H
0.958585	0.001713	95.377884	481.88147	2.3162904	0.000566	BRD1
0.16022	0.000024	218.006592	1132.99512	2.3129066	0.000223	TFAM
0.000753	0.000002	559.690002	2935.27026	2.3095215	0.000133	MAGOH
0.000008	0.000001	1558.53748	7886.02881	2.3081478	0.000106	SEP15
0.004454	0.000003	414.002899	2184.7207	2.3072873	0.000151	YIPF3

0.070103	0.000013	257.834717	1349.0769	2.306774	0.000199	ZBTB2
0.000008	0.000001	1559.58557	7853.521	2.3011278	0.00018	EEF1D
0.136579	0.000021	226.391449	1166.45911	2.296907	0.00023	RYBP
0.817167	0.000441	121.580597	610.000793	2.2942585	0.000439	C11orf96
0.135609	0.000024	226.391449	1162.63464	2.2921779	0.000249	PPP1R15A
0.854414	0.000582	116.34005	580.361206	2.2896173	0.000474	ITFG2
0.215853	0.000026	218.006592	1113.87292	2.2883959	0.00032	SPTSSA
0.997365	0.036484	69.175163	339.420502	2.2848677	0.00717	FBXW7
0.149964	0.000032	221.150909	1128.2146	2.2848429	0.000301	ZNF131
0.000029	0.000001	1092.12915	5490.96289	2.2837836	0.000122	AP3D1
0.326472	0.000014	255.738495	1315.61292	2.283132	0.003317	ZCCHC8
0.85328	0.000598	116.34005	577.492859	2.2824469	0.000489	RAB30
0.302745	0.000051	186.563324	942.728455	2.2817402	0.000286	DIP2B
0.316879	0.000005	368.934204	1912.22815	2.2800806	0.013268	EVA1A
0.910672	0.001013	106.907074	526.818848	2.27897	0.000554	UQCRB
0.859947	0.000645	115.291946	569.843994	2.2770026	0.000506	WARS
0.000026	0.000001	1129.86108	5646.80957	2.2768503	0.000124	LOC100653071|CRTAP
0.995396	0.010845	73.3676	355.674438	2.2650217	0.001035	PRRG4
0.000012	0.000001	1383.5033	6806.57617	2.2648007	0.000125	COX7A2L
0.000094	0.000001	833.246338	4210.72607	2.2644885	0.000138	TRIB3
0.001448	0.000002	500.995911	2539.43896	2.2578597	0.000166	ZNF259
0.532411	0.000133	155.120071	766.803467	2.2577287	0.000376	CYB5D2
0.074782	0.000015	254.690384	1282.14893	2.2523329	0.000244	AGPAT2
0.153248	0.000027	220.102798	1096.66284	2.2512886	0.000276	GFM2
0.56739	0.000169	148.831421	731.427246	2.2508798	0.000582	C9orf85
0.070176	0.000015	257.834717	1297.44678	2.2505828	0.000244	RAB11FIP1
0.992169	0.011888	73.3676	350.89386	2.2462051	0.001297	previous version conserved probe
0.014959	0.000006	338.539062	1706.66357	2.2382971	0.000213	PARD3
0.000086	0.000001	851.064148	4214.55078	2.2361898	0.000151	EGR1
0.001002	0.000002	532.439148	2641.74316	2.2294674	0.000179	OR4D9
0.011548	0.000006	355.308807	1775.50378	2.2252408	0.000219	TMEM98
0.000031	0.000001	1077.45557	5201.26025	2.2216819	0.000151	CNIH
0.000088	0.000001	845.823608	4145.71045	2.2211784	0.000159	NUDT5
0.003775	0.000004	425.532074	2117.79272	2.2207371	0.000202	LPIN2
0.000082	0.000001	858.40094	4204.03369	2.2205919	0.000159	MSANTD3|MSANTD3-TMEFF1
0.881267	0.000961	112.147621	531.599426	2.2187856	0.000648	FAM210A
0.761728	0.00213	98.522209	464.671417	2.2168618	0.006246	previous version conserved probe
0.438661	0.003832	166.649261	803.135803	2.2167758	0.009936	CREB5
0.000006	0.000001	1758.7262	8323.92871	2.2158838	0.000143	CLTA
0.000062	0.000001	912.902588	4438.28125	2.2158496	0.00016	TAF9
0.042818	0.000002	592.181335	2899.89404	2.2109546	0.000466	CLASP1
0.117952	0.000024	232.680099	1131.08289	2.210514	0.000306	DNTTIP1
0.007672	0.000005	378.367188	1871.11523	2.2097919	0.000222	CHODL-AS1
0.000278	0.000002	672.885681	3287.12012	2.2079033	0.000178	TRPC4
0.00849	0.000005	372.078552	1837.65125	2.2078216	0.000226	LSM4

0.623488	0.000228	144.638977	689.358215	2.2065771	0.000492	RBM48
0.067174	0.000016	259.930939	1263.98279	2.2005297	0.000291	CRABP2
0.025145	0.000003	439.157501	2151.25659	2.1988893	0.000315	PCDHA3
0.000779	0.000002	634.105713	3070.08228	2.1944936	0.000201	CDIPT
0.001393	0.000003	503.092102	2437.13477	2.1902766	0.00021	FUS
0.954908	0.002709	96.425987	446.505249	2.1901716	0.000894	ZNF394
0.015957	0.000004	410.858551	2004.01501	2.1893953	0.00028	RPS19BP1
0.741986	0.000437	131.013565	610.956909	2.1821375	0.000607	LAMTOR1
0.01293	0.000006	346.92395	1682.76074	2.1806014	0.000259	RNF6
0.000004	0.000001	2032.28259	9285.7793	2.1753514	0.000162	DUSP14
0.001074	0.000002	527.198608	2508.84326	2.1663939	0.000225	HELZ
0.000003	0.	2305.83887	10401.5645	2.166232	0.000165	HSPA1B
0.997101	0.024398	69.175163	310.737061	2.1620957	0.001728	ENTPD7
0.883564	0.00136	110.051399	501.003754	2.1618793	0.00088	TOE1
0.001296	0.000003	509.380768	2418.96851	2.1612075	0.000232	GTF2IRD1
0.000001	0.	8346.08887	36398.3047	2.1596252	0.000157	EIF3H
0.993217	0.01333	76.511925	344.20105	2.1588855	0.001462	ST6GALNAC
0.488675	0.000157	160.360611	741.944519	2.157884	0.000527	KLHL15
0.000333	0.000002	648.779236	3060.521	2.1571148	0.000216	SAE1
0.893613	0.001391	110.051399	499.091522	2.1563447	0.000843	DCP1A
0.130111	0.000028	232.680099	1088.05774	2.1546698	0.000398	previous version conserved probe
0.000021	0.000001	1191.69946	5471.84082	2.152414	0.00019	MARCH7
0.365858	0.000034	222.19902	1033.55933	2.150212	0.005869	BCKDK
0.000001	0.	4282.57178	18697.7656	2.1485032	0.000168	TPM2
0.001037	0.000003	529.294861	2482.07202	2.1446218	0.000243	TUFT1
0.000105	0.000001	816.476562	3790.03613	2.1417553	0.000214	LURAP1L
0.898353	0.032166	67.078949	296.395355	2.1407843	0.019884	CASP7
0.000003	0.	2501.83521	11018.2588	2.1387001	0.000181	AJUBA
0.000007	0.000001	1662.30017	7483.50488	2.1383014	0.000189	EEF1B2
0.00002	0.000001	1211.61353	5506.26074	2.1379065	0.000199	TTC1
0.000001	0.	4007.96729	17305.6641	2.1334485	0.000178	PSMA1
0.000029	0.000001	1092.12915	4966.05615	2.1324195	0.000208	KLF10
0.724246	0.000489	133.109787	598.527405	2.1280906	0.000732	TMEM119
0.881373	0.002573	101.666534	450.329712	2.1261646	0.001665	POLB
0.003455	0.000004	431.82074	2013.57617	2.1240719	0.000286	BTF3
0.994775	0.023963	71.271385	311.693176	2.1232991	0.002061	RQCD1
0.000032	0.000001	1069.0708	4825.50781	2.1200137	0.000218	ADAMTS6
0.649723	0.000532	141.494659	633.903625	2.1176869	0.001045	DGKE
0.000247	0.000001	811.236023	3695.38086	2.1144387	0.00025	CUL1
0.960342	0.004819	93.281662	409.216827	2.1135618	0.001408	FLRT3
0.750762	0.000602	129.965469	577.492859	2.1129074	0.000804	CYP2B6|CYP2B7P1
0.050976	0.000016	273.556335	1253.46558	2.1116075	0.000391	MBIP
0.161872	0.000036	225.343338	1021.12982	2.1106666	0.000514	SSNA1
0.000538	0.000002	594.277588	2710.58325	2.1089115	0.000266	previous version conserved probe
0.930839	0.002607	102.714638	449.373596	2.1082915	0.001117	UBB

0.861391	0.001271	115.291946	506.740448	2.1071435	0.000955	previous version conserved probe
0.505967	0.000202	158.264389	705.612183	2.1034021	0.000658	ALPK3
0.585714	0.000295	147.78331	656.850342	2.1025839	0.000835	AP3S1
0.915873	0.023191	105.858963	460.846985	2.1004184	0.020696	previous version conserved probe
0.000037	0.000001	1032.38696	4606.55762	2.099821	0.000236	SUMO1|SUMO1P3
0.235773	0.000065	199.140625	891.098328	2.0979117	0.000534	HIF1A
0.000002	0.	3117.07495	13222.1016	2.0966609	0.000207	IGFBP3
0.929209	0.014898	78.608147	337.50827	2.0925874	0.005638	NAA60
0.972907	0.006359	90.137337	388.182312	2.089653	0.001452	RIPK1
0.000001	0.	3412.6416	14349.3594	2.0880787	0.000212	EIF4G1
0.961747	0.004809	94.329773	406.34848	2.0876615	0.001368	LOC100652740
0.46334	0.000182	163.504944	719.953857	2.0834403	0.000688	TOP1MT
0.015484	0.000008	338.539062	1532.65088	2.0825619	0.000382	DR1
0.000655	0.000002	576.459717	2580.55176	2.0801336	0.000301	SAT2
0.930843	0.002969	102.714638	439.812469	2.0771658	0.00126	previous version conserved probe
0.133701	0.000032	236.872543	1050.76929	2.0766236	0.000568	DHRS7
0.181404	0.000041	224.295242	992.446411	2.075473	0.00065	PPP2CB
0.000001	0.	8943.51074	36742.5078	2.0746717	0.000213	JUN
0.030502	0.000013	299.759064	1341.42798	2.0725319	0.000427	TCEAL3|TCEAL4
0.321761	0.000106	183.419006	803.135803	2.0696393	0.000645	IK
0.964793	0.005644	93.281662	395.831207	2.0673171	0.001506	FGD4
0.000001	0.	5649.30518	23114.0566	2.066041	0.000224	previous version conserved probe
0.212277	0.000064	204.38118	893.966614	2.0628272	0.000599	C6orf132
0.035137	0.000015	292.422302	1297.44678	2.0615715	0.000453	DENND1B
0.000606	0.000002	581.700256	2570.03467	2.0610032	0.000321	ZNF317
0.550334	0.000288	153.023849	661.63092	2.0597352	0.00082	PROSC
0.955184	0.004739	96.425987	407.304596	2.0592202	0.001479	ICAM4
0.000029	0.000001	1091.08105	4726.07178	2.0591909	0.000272	SEPW1
0.964943	0.015966	79.65625	333.683807	2.0576318	0.00343	NRIP3
0.858532	0.037577	79.65625	333.683807	2.0576318	0.032481	HDAC7
0.032353	0.000001	2306.88696	9630.93652	2.0528925	0.001242	MYLK
0.955186	0.004877	96.425987	405.392365	2.0526766	0.001517	MED10
0.006959	0.000006	384.655853	1712.40027	2.0524121	0.000397	EWSR1
0.997739	0.043561	68.12706	281.097534	2.0447298	0.002722	DNAL1
0.015812	0.00001	335.394745	1476.24011	2.042511	0.000446	CHAC1
0.493194	0.000228	161.408722	690.314331	2.040709	0.012414	AKT1
0.000032	0.000001	1070.1189	4575.96191	2.0386066	0.000296	HDLBP
0.01837	0.00001	327.009888	1431.30273	2.0358435	0.000465	HMGCL
0.000172	0.000002	736.820312	3174.29858	2.0307481	0.000334	ALKBH1
0.355525	0.000154	178.178452	759.154541	2.030417	0.000836	ABI2

0.002845	0.000007	446.494263	1952.38489	2.0298018	0.000552	STARD10
0.817176	0.001287	121.580597	506.740448	2.0258424	0.00124	NIPBL
0.000005	0.000001	1940.04895	8010.32373	2.0234711	0.000285	SRSF10|LOC100996657
0.994745	0.028243	74.41571	303.088165	2.0220865	0.002562	DGCR2
0.41514	0.000186	169.793594	717.08551	2.0196518	0.000852	TIMM44
0.438654	0.000207	166.649261	702.743835	2.018309	0.000869	ATXN1L
0.001358	0.000003	508.332672	2191.41333	2.0162226	0.000404	DPM2
0.044968	0.000019	279.845001	1199.9231	2.0147697	0.000561	CDKL3
0.000002	0.	2893.82788	11651.2061	2.0146723	0.000283	SBDS|SBDSP1
0.	0.	16456.3535	62637.8984	2.0136933	0.000265	RPSAP58|RPSA
0.005119	0.000006	404.569916	1754.46924	2.0133029	0.00045	HIST3H2A
0.002867	0.000004	445.446136	1921.78931	2.0095284	0.000437	previous version conserved probe
0.006517	0.000006	388.848297	1681.80457	2.0092092	0.000467	CDO1
0.000002	0.	3106.59375	12429.4824	2.0088328	0.000288	LATS2
0.958541	0.00636	95.377884	388.182312	2.0081231	0.00184	PDXDC1
0.000001	0.	8267.48047	32380.7148	2.0078018	0.000275	GPX4
0.997098	0.077493	68.12706	273.448608	2.0063684	0.010585	HAUS1
0.926112	0.003785	103.762749	422.602417	2.004748	0.001651	ETS1
0.74197	0.000877	131.013565	540.204468	2.0040069	0.001215	MSRB1
0.706101	0.000724	135.206009	558.370605	2.003658	0.00117	NA
0.660499	0.000581	140.446548	580.361206	2.0013509	0.001126	IKBIP
0.060571	0.000001	833.246338	3481.21118	1.9912359	0.001984	DUSP16
0.00047	0.000002	608.951111	2560.47339	1.9895816	0.000417	UBXN6
0.00005	0.000001	1159.20813	4779.61426	1.9886158	0.000368	SCNM1|TNFAIP8L2-SCNM1
0.000343	0.000002	645.634888	2706.75879	1.9876828	0.000412	ZFY
0.002833	0.000004	475.841309	2015.4884	1.9858914	0.000486	NEDD9
0.005719	0.000006	397.233154	1690.40967	1.9849596	0.000508	previous version conserved probe
0.000018	0.000001	1238.86438	5074.09717	1.982625	0.000397	POLR1C
0.817143	0.001525	121.580597	491.442627	1.9814778	0.00146	previous version conserved probe
0.153264	0.000061	220.102798	913.088928	1.980561	0.000803	ASGR1
0.655126	0.000664	139.398438	566.975647	1.9790459	0.001433	LOC732265|LOC646670|LOC440292|COMMD4
0.642048	0.000582	142.54277	580.361206	1.9786717	0.001212	FYN
0.23579	0.000096	199.140625	822.258057	1.9784133	0.000862	LOC442459|RCC2
0.001518	0.000004	495.755371	2084.32861	1.9772265	0.000474	PDHA1
0.00001	0.000001	1472.59253	5963.2832	1.9760648	0.000356	UBE2M|UBE2MP1

0.000014	0.000001	1329.00171	5403.00049	1.9757228	0.000362	MRPL19
0.000098	0.000001	885.651733	3639.92627	1.9699309	0.000411	SDF2
0.55033	0.000398	153.023849	621.474121	1.9691155	0.00116	WARS2
0.614725	0.001183	127.869247	513.433228	1.9676323	0.014626	NRG1
0.000129	0.000002	781.888977	3216.36768	1.9665953	0.000422	GZF1
0.000011	0.000001	1427.52393	5735.72803	1.9623822	0.000377	NUTF2|LOC128322
0.000234	0.000002	693.8479	2854.00049	1.9620557	0.000445	MAP2K3
0.000037	0.000001	1032.38696	4202.12109	1.9617241	0.000401	SSU72
0.027779	0.000028	311.288239	1290.75403	1.9606638	0.001002	IL12A
0.000151	0.000002	756.734375	3101.63403	1.9601767	0.000437	NA
0.893552	0.003173	110.051399	435.031891	1.9575304	0.001837	STRN3
0.995396	0.040342	73.3676	284.921997	1.9566053	0.003385	INMT
0.767919	0.001244	127.869247	508.652679	1.9540935	0.001528	ZBTB8OS
0.000003	0.000001	2322.60864	9056.3125	1.9533898	0.000369	ZNF207
0.955186	0.007641	96.425987	376.708923	1.9506373	0.002276	PDLIM7
0.728271	0.006264	99.570313	389.138428	1.949483	0.027951	HAUS2
0.000003	0.000001	2471.43994	9571.6582	1.9482253	0.000372	HSPB8
0.971498	0.012174	90.137337	348.981628	1.9416248	0.002659	GCDH
0.000498	0.000003	602.662415	2452.43262	1.9399492	0.00051	PEPD
0.005201	0.000007	403.52179	1664.5946	1.9389922	0.000606	ILKAP
0.666724	0.001034	133.109787	524.906616	1.9381311	0.002795	TIMMDC1
0.023586	0.000015	313.38446	1278.32446	1.9366974	0.00071	MBOAT7
0.143016	0.000046	238.96875	963.762939	1.9346391	0.001091	ARV1
0.035603	0.00002	293.470398	1189.40588	1.931025	0.000766	GCLM
0.004067	0.000006	420.291534	1723.87366	1.930367	0.000615	UGP2
0.006454	0.000005	466.408325	1904.57922	1.9299259	0.000686	ARPC5
0.831575	0.00406	107.955185	417.821838	1.929144	0.003908	CA2
0.000928	0.000003	539.77594	2188.54517	1.9279496	0.000557	CNBP
0.067173	0.000033	259.930939	1045.03271	1.9259359	0.000845	TRIM56
0.069732	0.000017	308.143921	1246.77271	1.9259189	0.001093	NRXN3
0.004006	0.000007	421.339661	1721.00525	1.9242913	0.000702	HSPA14
0.000778	0.000003	556.545654	2247.82422	1.9239729	0.00056	previous version conserved probe
0.926053	0.0054	103.762749	398.699554	1.9237337	0.002285	TBCE
0.106374	0.000001	1608.84668	6269.23975	1.923529	0.007999	RPS4X
0.056647	0.000029	268.315796	1076.58447	1.9214753	0.000841	E2F4
0.092824	0.000044	244.209305	976.192444	1.9203962	0.000907	SMAD3
0.006506	0.000008	388.848297	1578.54431	1.9179325	0.000673	CAPZB
0.660522	0.000807	140.446548	547.853333	1.9179246	0.001575	ZBTB1
0.997693	0.073162	68.12706	256.238556	1.9175208	0.004622	LRIG2
0.000001	0.	3585.57935	13425.7539	1.9166668	0.00041	HSP90AA1
0.000003	0.000001	2375.01392	9017.11133	1.9160715	0.000424	CLK1
0.601381	0.000627	146.735199	572.712341	1.9150959	0.001597	FAM221A
0.103487	0.000049	238.96875	950.37738	1.9138515	0.000948	TMEM217
0.000016	0.000001	1286.02917	5023.42334	1.913498	0.000466	HNRNPR
0.996645	0.073032	69.175163	259.106903	1.9105315	0.005807	NUP35
0.221224	0.000113	202.284958	798.355225	1.9105258	0.001134	CCNC
0.173542	0.000016	317.576904	1272.58777	1.9103547	0.003552	BLZF1
0.000309	0.000002	658.212158	2616.88428	1.9103466	0.000557	POLG2
0.000056	0.000001	935.960938	3681.99512	1.909135	0.000507	TNFRSF10B
0.	0.	10738.9209	39217.8867	1.9076587	0.000404	SERTAD2

0.153248	0.000073	220.102798	869.107666	1.9071885	0.001043	ACOT2
0.004129	0.000006	419.243439	1690.40967	1.9046018	0.000683	RTN3
0.642037	0.000783	142.54277	550.72168	1.9028031	0.001647	NA
0.000005	0.000001	1820.56458	6934.69531	1.9005621	0.000466	H2AFZ
0.000001	0.	4960.69775	18098.2832	1.899783	0.000429	YWHAG
0.011407	0.000011	354.260712	1415.04883	1.8997436	0.000765	STUB1
0.854418	0.002855	116.34005	442.680817	1.8976893	0.002166	BCAS2
0.0005	0.000003	602.662415	2383.59229	1.8973123	0.000614	RASSF8
0.917833	0.005729	104.81086	394.875092	1.8958317	0.00259	CDC42BPA
0.647473	0.00305	115.291946	437.900238	1.8958281	0.035299	CHRNA10
0.578546	0.000621	148.831421	573.668457	1.8957958	0.001877	CTAGE11P|LOC101060696|CTAGE4|CTAGE9
0.50597	0.000441	158.264389	610.000793	1.8905994	0.001539	MYLIP
0.992307	0.038109	77.560036	287.790344	1.8903638	0.004023	UNC13D
0.953331	0.010669	95.377884	356.630554	1.8902428	0.003122	HIST2H2BF|HIST1H2BN|HIST1H2BE|HIST1H2BK|HIST1H2BL|HIST2H2BC|HIST1H2BI|HIST2H2BA|HIST1H2BC|HIST1H2BJ|HIST1H2BD|HIST3H2BB|HIST1H2BO|HIST1H2BH|HIST1H2BM
0.035146	0.000023	292.422302	1151.16138	1.889311	0.000902	previous version conserved probe
0.303756	0.000128	199.140625	773.496277	1.887551	0.002154	CHORDC1|LOC727896
0.000257	0.000002	682.318665	2663.73364	1.8864036	0.000608	MAX
0.768007	0.001654	127.869247	484.749817	1.8844319	0.002024	DDX27
0.00001	0.000001	1534.43091	5831.33984	1.8829019	0.000514	RALY
0.015522	0.000014	336.442841	1323.26184	1.8803152	0.000854	FGF2
0.044968	0.000028	279.845001	1092.83838	1.8801618	0.000966	DRG2
0.000588	0.000003	584.844604	2285.11255	1.8772856	0.000667	CHKA
0.000003	0.000001	2264.96265	8390.85742	1.8765229	0.0005	previous version conserved probe
0.160082	0.00013	218.006592	840.424255	1.871993	0.001628	SPSB3
0.021839	0.000017	318.625	1242.94824	1.8714942	0.000921	MAPKAP1
0.001033	0.000004	530.342957	2069.98682	1.8698995	0.000711	LIMS1
0.353563	0.00023	180.274673	688.4021	1.8670613	0.001659	MTRNR2L3
0.000043	0.000001	992.558838	3780.47485	1.8649001	0.000599	ZRANB1
0.009821	0.000011	363.693665	1416.00488	1.8614325	0.000889	SERPINB8
0.78487	0.002028	125.773026	468.49588	1.8603746	0.00228	PKP2

0.053197	0.000006	452.782898	1766.8988	1.860197	0.001963	LRRC41
0.000024	0.000001	1144.53455	4333.10889	1.859285	0.000594	HEXIM1
0.568059	0.000664	150.927643	566.975647	1.8574012	0.001872	RAB8B
0.856585	0.004351	112.147621	413.04126	1.8548186	0.00324	AP1S3
0.000001	0.	4309.82227	15365.709	1.8547198	0.000519	RSRC2
0.000005	0.000001	1835.23816	6769.2876	1.854073	0.000562	KLHL5
0.005173	0.000008	407.714233	1586.19324	1.8540698	0.000861	TGS1
0.980351	0.023959	85.944901	311.693176	1.8532101	0.004067	CWF19L1
0.99587	0.066918	72.319489	260.063019	1.8513766	0.005271	C11orf91
0.042255	0.000029	282.989319	1083.27722	1.8508053	0.001088	EXOSC7
0.010545	0.000012	359.501251	1386.36536	1.8483034	0.000939	AKIRIN1
0.001137	0.000004	520.909973	2004.01501	1.8472045	0.000787	LOC441155
0.000159	0.000002	748.349548	2835.83423	1.8470884	0.000694	FBXW7
0.134456	0.000078	226.391449	857.634277	1.8442067	0.00132	CCDC93
0.000002	0.000001	2651.7146	9531.50098	1.8439275	0.000563	SLC7A11
0.000002	0.000001	2794.25757	10031.5488	1.8429197	0.000563	ID2
0.007302	0.00001	381.511536	1468.59119	1.8424411	0.000929	EIF2AK1
0.443817	0.000244	181.322784	680.753235	1.8415586	0.02613	SNX16
0.000019	0.000001	1216.854	4541.54199	1.8414982	0.000632	SERTAD1
0.757927	0.005245	110.051399	400.611786	1.8412161	0.010957	VPS53
0.001393	0.000004	503.092102	1925.61365	1.8374926	0.000831	RIT1
0.50597	0.00054	158.264389	588.010132	1.8374608	0.001913	TSC22D1
0.000003	0.000001	2479.82495	8891.86035	1.835656	0.000586	SRSF6
0.362601	0.000286	177.130356	660.674805	1.8334568	0.001728	PMP22
0.001073	0.000004	526.150513	1997.32227	1.8277992	0.000849	FUNDC2
0.935384	0.009375	101.666534	364.279449	1.8276327	0.003468	QKI
0.079249	0.000049	252.594162	949.421265	1.8272621	0.001307	OR10AG1
0.182317	0.000114	211.717926	790.706299	1.8262758	0.001507	DCTN6
0.000462	0.000003	611.047302	2303.27881	1.826114	0.000809	SNRPN|SNURF
0.000001	0.	9414.11133	32373.0664	1.8228856	0.000574	OR1I1
0.000022	0.000001	1168.64111	4308.25	1.8204902	0.000695	GGNBP2
0.038441	0.00001	378.367188	1433.21497	1.8197013	0.001618	MYOM2
0.930839	0.009229	102.714638	365.235565	1.8164806	0.00358	C8orf33
0.793132	0.002575	124.724922	450.329712	1.8159486	0.002766	NRCAM
0.00098	0.000004	534.5354	2010.70789	1.8150645	0.00089	SMNDC1
0.559364	0.000753	151.975739	554.546143	1.8147263	0.002199	SEC14L1|SEC14L1P1
0.975323	0.026246	86.993012	306.912598	1.8142305	0.004926	CNOT4
0.958584	0.018082	95.377884	337.50827	1.8136112	0.005444	EIF4E
0.256621	0.000183	194.948196	719.953857	1.8135852	0.001714	POLR3B
0.994482	0.127132	66.030838	229.467377	1.813547	0.009801	ADAT1
0.943834	0.011581	99.570313	351.849976	1.8094163	0.003846	PIK3CA
0.002149	0.000006	467.456421	1760.20593	1.8086477	0.00097	ARPC2
0.003779	0.000007	440.205597	1657.90173	1.8061479	0.001034	C11orf91
0.678893	0.001391	138.350327	499.091522	1.8060311	0.002539	GUSBP2
0.442046	0.000405	169.793594	619.56189	1.8053852	0.004134	LTV1
0.614328	0.001252	145.687088	526.818848	1.8051818	0.002901	LOC653375|WBSCR16
0.000055	0.000001	940.153381	3438.18604	1.8044839	0.00078	SDHAF2
0.145783	0.000002	693.8479	2560.47339	1.8037534	0.014737	previous version conserved probe

0.000006	0.000001	1748.24512	6246.29297	1.8008285	0.000706	SIRT1
0.523527	0.00068	156.168182	565.063416	1.8002605	0.002276	WDR18
0.137421	0.000102	225.343338	827.994751	1.7990533	0.001722	GNG12
0.014697	0.000012	376.270966	1400.70703	1.7949687	0.001345	MRPS9
0.714871	0.001798	134.157898	478.057037	1.7908174	0.002816	COMMD3
0.000002	0.	3338.22583	11520.2178	1.7906928	0.000689	OSTC
0.001164	0.000005	518.813721	1919.87708	1.7887722	0.001007	PLEKHB2
0.230819	0.000173	200.188736	727.602783	1.7885857	0.001855	POLG
0.874809	0.00548	113.195724	397.743439	1.7881002	0.003517	BMP1
0.000044	0.000001	989.41449	3572.04199	1.7878865	0.000827	SLC39A7
0.005049	0.000009	405.618011	1505.87964	1.7869547	0.001136	MB21D2
0.103503	0.000132	238.96875	867.195435	1.7777151	0.002427	SENP5
0.000003	0.000001	2223.03833	7699.58643	1.7765657	0.00076	previous version conserved probe
0.000001	0.	6762.39648	22454.3379	1.7762117	0.000705	GPNMB
0.88461	0.010328	103.762749	358.542755	1.7761167	0.005978	IPO9
0.000112	0.000002	803.899292	2893.20117	1.7756718	0.000918	SMARCA4
0.961792	0.018998	94.329773	324.12265	1.7732817	0.004801	RBCK1
0.823145	0.005267	120.532486	421.646301	1.7731134	0.004931	SLC4A1AP
0.000095	0.000002	832.198181	2969.69019	1.764745	0.000953	CGRRF1
0.019079	0.000019	332.250427	1204.70374	1.763895	0.001448	ECHDC2
0.000638	0.000006	576.459717	2086.24097	1.7617011	0.001418	USP36
0.047939	0.00004	276.700653	995.314758	1.7595124	0.001616	ENTPD4
0.996497	0.103925	71.271385	239.028519	1.7585075	0.007729	ELFN2
0.020983	0.000016	350.068268	1265.89502	1.7571486	0.001591	previous version conserved probe
0.00001	0.000001	1471.54443	5145.80566	1.7549851	0.000879	NINJ1
0.186294	0.000149	210.66983	749.593445	1.7545301	0.002047	STK11
0.043147	0.000038	281.941193	1010.61255	1.7541352	0.001632	FKBP10
0.861385	0.005644	115.291946	395.831207	1.7533096	0.003962	previous version conserved probe
0.801274	0.003534	123.676811	427.382996	1.7531741	0.003618	EXOSC10
0.997374	0.124579	69.175163	230.423492	1.7520495	0.008302	GALR2
0.839977	0.00474	118.436272	407.304596	1.7518511	0.003847	ANKRD1
0.070103	0.000056	257.834717	920.737854	1.7501552	0.001769	FAM24B
0.431256	0.00054	167.697372	588.010132	1.7488009	0.002642	LOC101060555|POLR3GL
0.595888	0.001164	147.78331	514.389343	1.7487571	0.002988	SRP19
0.000009	0.000001	1542.8158	5359.01904	1.7477819	0.0009	ZFAND2A
0.000798	0.000004	556.545654	1996.36609	1.7463157	0.001176	BACH1
0.225969	0.000195	201.236847	710.392761	1.7449945	0.00222	PSMG2
0.586709	0.001152	148.831421	515.345459	1.7406261	0.003067	KAT8
0.939729	0.014752	100.618423	338.464386	1.7403768	0.005075	ALG14
0.045923	0.000041	278.796875	989.578064	1.7396389	0.00175	PTPN21
0.000034	0.000001	1050.20483	3655.22412	1.7374411	0.001011	GPS1
0.164034	0.000136	216.958481	762.979004	1.7356992	0.002167	NEK7
0.003888	0.000009	423.435852	1515.4408	1.7326264	0.001401	DCAF6
0.000001	0.	5872.55225	18936.7949	1.731062	0.00086	MMADHC

0.000878	0.000004	545.016479	1935.1748	1.7298006	0.00127	SRF
0.418211	0.000452	175.034134	607.132446	1.7294083	0.005914	SLC30A5
0.000185	0.000002	743.109009	2595.84961	1.7283381	0.001161	PDHX
0.000428	0.000003	619.43219	2184.7207	1.7273768	0.001224	CRCP
0.328326	0.000288	189.707657	659.718689	1.7260924	0.003811	ZNF544
0.750769	0.002892	129.965469	441.724701	1.7250204	0.003829	LY6G5B
0.000104	0.000002	816.476562	2834.87817	1.7247032	0.001137	LOC101060301|LOC649330|LOC440563|HNRNPCL1
0.019822	0.000024	322.817444	1137.77576	1.7246434	0.001692	HMG20B
0.847265	0.005729	117.388161	394.875092	1.722364	0.004399	GBGT1
0.041401	0.00004	284.037415	996.270874	1.7218191	0.001864	CBY1
0.682666	0.004808	120.532486	406.34848	1.7217262	0.020301	SLC41A3
0.000002	0.	7130.28271	22772.7246	1.7208128	0.000938	TFB2M
0.182329	0.000163	211.717926	736.207825	1.7201337	0.002359	ELP5
0.001518	0.000006	495.755371	1754.46924	1.7192326	0.001381	UBR2
0.000011	0.000001	1477.83313	5043.50146	1.7185701	0.001032	MED30
0.000028	0.000001	1102.61023	3787.16772	1.7179873	0.001088	TFPI2
0.001034	0.000005	530.342957	1870.15906	1.7178727	0.001353	ANKRD13A
0.93527	0.015158	101.666534	336.552155	1.7175484	0.005443	CP
0.000034	0.000001	1066.97449	3660.00464	1.7164201	0.001108	ID2
0.000052	0.000002	952.730713	3275.64673	1.7163354	0.001134	LINC00674
0.012258	0.000018	350.068268	1229.56262	1.7152149	0.001677	GLIPR1
0.000087	0.000002	865.737671	2978.29517	1.7136206	0.001183	TAB2
0.000251	0.000003	684.414917	2377.85571	1.7120986	0.001265	JMJD1C
0.000012	0.000001	1392.93628	4741.36963	1.7110246	0.001073	ASCC3
0.000258	0.000003	681.270569	2365.42627	1.7106929	0.001275	TMEM208
0.023149	0.000027	314.432587	1095.70667	1.7097324	0.001836	TMEM99
0.000722	0.000013	563.882385	1968.63879	1.7065369	0.002425	TRIM58
0.000087	0.000002	848.967957	2907.54272	1.7064565	0.001218	PATL1
0.881219	0.03243	112.147621	370.97226	1.7054494	0.027803	RNF121
0.89431	0.010161	109.003296	359.498871	1.705298	0.005416	LECT1
0.662372	0.002211	137.302231	461.803101	1.705289	0.00511	MARCH5
0.00383	0.000009	424.483978	1490.58179	1.7052544	0.001576	PAWR
0.99598	0.117275	72.319489	233.291824	1.7046307	0.009324	IFT20
0.001463	0.000006	498.899689	1747.77649	1.7044167	0.001469	THYN1
0.053167	0.000052	271.460114	939.860107	1.7028901	0.002087	ZKSCAN1
0.48869	0.000849	160.360611	543.072754	1.7022574	0.003333	PPP4R2
0.930753	0.01542	102.714638	335.596039	1.6987956	0.005797	C10orf137
0.328306	0.000401	182.370895	620.518005	1.697959	0.002973	PHLDA3
0.002066	0.000007	470.600739	1643.56006	1.6976176	0.001556	TMEM141
0.98347	0.052864	83.848686	271.536377	1.69705	0.007962	RGMB
0.363447	0.000435	180.274673	611.912964	1.6954889	0.003892	NR2C2
0.559371	0.001216	151.975739	510.564911	1.6951337	0.00363	HIST1H2BH|HIST1H2BL|HIST1H2BE|HIST1H2BK|HIST2H2BF|HIST1H2BN|HIST1H2BM|HIST2H2BC|HIST2H2BA

0.586424	0.001391	148.831421	499.091522	1.6942435	0.003747	SNAPC5
0.000099	0.000002	825.909546	2799.50195	1.6905963	0.001315	BRF2
0.125249	0.000058	267.2677	916.913391	1.689419	0.003707	COQ3
0.001233	0.000001	982.077759	3311.02295	1.6893172	0.001782	CCRN4L
0.005651	0.000012	400.377472	1387.32153	1.6883123	0.001758	DLG1
0.447141	0.000738	165.601151	556.458374	1.6882709	0.003447	SMTN
0.000238	0.000003	691.751648	2361.60181	1.6869091	0.001407	PABPC4
0.002729	0.000008	449.63858	1558.46594	1.6865931	0.001664	JMJD6
0.976777	0.041914	88.041122	283.009766	1.6842071	0.007668	SCN8A
0.926055	0.015417	103.762749	335.596039	1.6841487	0.006071	CNRIP1
0.887522	0.009843	111.09951	361.411102	1.6835155	0.005558	MFSD5
0.021822	0.000028	317.576904	1087.10168	1.6835042	0.002044	CAPN7
0.007298	0.000014	381.511536	1314.65686	1.6829954	0.001837	LETMD1
0.012032	0.000019	351.116364	1205.65979	1.682477	0.00193	IFRD1
0.000028	0.000001	1126.71667	3769.95776	1.6801851	0.001282	SRM
0.001812	0.000007	481.081848	1657.90173	1.678451	0.001704	EZH2
0.037348	0.000042	289.277954	984.797485	1.6772735	0.002232	WDR83
0.875598	0.026834	95.377884	305.956482	1.6771365	0.017224	ARL17B|LOC100996709|ARL17A
0.099094	0.000097	241.064972	817.477539	1.6765605	0.002556	ADAMTS5
0.000215	0.000003	705.377075	2387.41675	1.6758996	0.001519	LXN
0.000008	0.000001	1619.32776	5346.58984	1.6743233	0.001229	ID2
0.000001	0.	3610.73413	11499.1836	1.6742354	0.001131	ATP6V0D1
0.005201	0.000012	403.52179	1384.45312	1.6736382	0.001856	VEGFA
0.019425	0.000026	325.961761	1109.09229	1.6733861	0.00212	HPX
0.000056	0.000002	935.960938	3123.62451	1.6729293	0.001375	DDX47
0.000722	0.000004	563.882385	1924.65759	1.6727014	0.001607	CLIP4
0.000005	0.000001	1838.38245	6006.30859	1.6720363	0.00122	PSMB2
0.674565	0.002541	137.302231	451.285828	1.6719468	0.005117	ARMC8
0.592787	0.001597	147.78331	487.618164	1.6714025	0.00436	TADA2A
0.029508	0.000008	452.782898	1552.72925	1.671261	0.003294	SENP2
0.111391	0.000024	332.250427	1129.17065	1.6706561	0.005819	previous version conserved probe
0.362684	0.000531	177.130356	589.922363	1.6695213	0.003458	MEX3B
0.000095	0.000002	832.198181	2777.51123	1.6686568	0.001445	previous version conserved probe
0.811591	0.009118	121.580597	394.875092	1.6686457	0.009071	AHR
0.086981	0.00514	247.353622	833.731445	1.6663038	0.034086	ADM
0.035143	0.000042	292.422302	986.709717	1.6639959	0.002348	RMDN1
0.000011	0.000001	1441.14929	4747.10645	1.6636632	0.001311	PRICKLE1
0.000003	0.000001	2450.47778	7809.53955	1.6621378	0.001233	FH
0.000007	0.000001	1642.38611	5373.36084	1.6614107	0.001298	RPL17
0.000003	0.000001	2168.53662	6938.51953	1.6586233	0.001269	ARIH1
0.377122	0.000598	175.034134	577.492859	1.6569703	0.003693	previous version conserved probe
0.001083	0.000003	640.394348	2149.34448	1.6550683	0.001872	CFLAR
0.085381	0.00009	248.401733	830.863098	1.654693	0.00275	MSN

0.000108	0.000002	820.669006	2712.49561	1.6541564	0.001553	MYLIP
0.007817	0.000015	379.415314	1280.23669	1.6530281	0.002114	FBXO34
0.189015	0.000059	272.50824	912.132812	1.6526216	0.008511	TMEM41A
0.002636	0.000009	451.734802	1525.95801	1.6495501	0.001952	CIRH1A
0.003128	0.000009	439.157501	1482.93286	1.6489738	0.001984	SARNP
0.00017	0.000003	738.916565	2446.6958	1.6476372	0.001639	U2AF1
0.505965	0.001157	158.264389	515.345459	1.6465537	0.004293	INO80B
0.000009	0.000001	1517.66125	4931.63623	1.6463419	0.001404	GOLGA4
0.438669	0.000841	166.649261	544.02887	1.6459072	0.004063	POLH
0.017042	0.000026	331.202301	1105.26782	1.6445699	0.002361	IRS1
0.000001	0.	4470.18311	13801.5068	1.6433352	0.001279	NFE2L2
0.003946	0.000011	422.387756	1420.78552	1.6433171	0.002073	MEF2A
0.008639	0.000017	371.030426	1241.99219	1.6427625	0.002226	UBE2V2
0.81692	0.013459	109.003296	343.244934	1.6409636	0.013876	KIAA1143
0.019184	0.000029	324.913666	1080.40894	1.6404696	0.002435	TMCO3
0.273095	0.000375	191.803864	628.166931	1.638301	0.003661	ZMYND11
0.963122	0.036013	93.281662	290.658661	1.6378738	0.00863	VPS37A
0.231206	0.000295	200.188736	656.850342	1.6372631	0.003545	SLC35F2
0.01203	0.000021	351.116364	1168.37134	1.6372383	0.002353	SYNE1
0.000001	0.	4697.62256	14376.1309	1.6360769	0.001318	CLK1
0.844737	0.008392	117.388161	370.97226	1.6355371	0.006425	ARMC6
0.207482	0.000285	205.429276	673.104309	1.6332155	0.003747	GGCT
0.839977	0.008067	118.436272	373.840607	1.63264	0.006374	SPTY2D1
0.966887	0.039586	92.233551	285.878113	1.6311154	0.008901	PAPD5
0.377023	0.000664	175.034134	566.975647	1.6303692	0.004134	SGSH
0.241085	0.000326	198.092514	644.420898	1.6257672	0.003759	SMG9
0.874717	0.012574	113.195724	353.762207	1.6251565	0.007974	AMDHD2
0.958578	0.033387	95.377884	294.483124	1.6239893	0.008664	GGT1|GGTLC2
0.446767	0.002499	166.649261	535.423889	1.6228324	0.011406	ZP3
0.079465	0.000055	281.941193	924.562317	1.6218681	0.00388	ZNF292
0.056705	0.000069	268.315796	879.624939	1.6218614	0.002994	MITF
0.000001	0.	3437.79614	10582.2705	1.6208187	0.001438	WAC
0.595887	0.001979	147.78331	470.408112	1.6193986	0.005158	ZNF674
0.00001	0.000001	1472.59253	4695.47607	1.6159921	0.001615	ACVR2A
0.000461	0.000004	611.047302	2001.14673	1.6155424	0.002009	DLC1
0.0003	0.000001	1593.125	5059.75537	1.6148364	0.002177	HSP90B1|HSP90B2P
0.165411	0.000182	222.19902	720.909973	1.6147764	0.003992	CHML
0.998268	0.245501	66.030838	197.915604	1.6137321	0.015358	KLF11
0.000354	0.000004	641.442444	2091.97754	1.6122038	0.002005	SEMA7A
0.6972	0.003439	136.25412	429.295197	1.6113766	0.005835	MAFG
0.267499	0.000404	192.851974	619.56189	1.6099752	0.004118	FAM83D
0.290649	0.000464	188.659546	604.264099	1.6075191	0.004248	TIAM2
0.355524	0.000657	178.178452	567.931763	1.6054858	0.004521	RAB4B|MIA-RAB4B
0.005282	0.000014	402.473694	1315.61292	1.6040834	0.002527	PXN
0.048483	0.000001	2806.83472	8550.52832	1.6025153	0.014705	ARPC3
0.000005	0.000001	1906.50952	5928.86328	1.6019842	0.001657	RBBP6
0.000112	0.000004	822.765259	2622.62085	1.601235	0.002443	GOLPH3
0.000018	0.000001	1303.84705	4127.54443	1.5992893	0.001796	SH3BP4
0.143285	0.000178	225.343338	723.77832	1.5991151	0.003904	EIF5
0.78487	0.006265	125.773026	389.138428	1.5963995	0.006829	NRG1
0.000001	0.	6970.97021	20365.2285	1.5951148	0.001555	BTF3

0.000007	0.000001	1641.33801	5138.15674	1.5949063	0.001745	AJUBA
0.000202	0.000003	713.761963	2284.15649	1.5931536	0.00211	AATF
0.007084	0.000001	1391.88818	4371.35352	1.5897089	0.004575	BDNF
0.106993	0.000133	240.016861	766.803467	1.5881354	0.003901	CHCHD5
0.207482	0.001243	205.429276	652.069763	1.5870361	0.010684	GNB1L
0.000543	0.000005	593.229431	1907.44751	1.5862876	0.002313	previous version conserved probe
0.997374	0.218276	69.175163	203.652298	1.5852158	0.015828	LOC100506124
0.471673	0.001257	162.456833	507.696564	1.5848881	0.005412	SMIM13
0.983144	0.076918	84.896797	253.370224	1.5848318	0.011886	RSBN1
0.000002	0.000001	3187.2981	9579.30664	1.5846578	0.001702	PCF11
0.981476	0.072458	85.944901	256.238556	1.5823366	0.011776	LIG4
0.001373	0.000007	505.188324	1627.30615	1.5813142	0.002519	NEDD9
0.000028	0.000001	1107.85071	3459.2207	1.5810456	0.001992	TLCD2
0.035876	0.000056	291.374176	928.386719	1.5788074	0.003476	STAG1
0.000002	0.000001	2925.271	8763.74121	1.5786861	0.001761	DNAJA1
0.000022	0.000001	1170.7373	3649.4873	1.5781434	0.001995	GNB2
0.000027	0.000001	1119.38	3487.90405	1.5779413	0.002016	IFI44L
0.182334	0.000269	211.717926	668.32373	1.5763984	0.004387	GSTK1
0.000039	0.000001	1127.76477	3509.89478	1.5761815	0.002088	SQLE
0.000003	0.000001	2382.35083	7167.03076	1.5755597	0.001821	ANXA6
0.921132	0.023109	104.81086	313.605408	1.5754103	0.009363	METTL25
0.97515	0.060636	89.089226	264.843597	1.5751161	0.01157	DUSP26
0.003521	0.000012	430.772614	1379.67261	1.5727659	0.00281	LZIC
0.000001	0.	6630.33496	19050.5723	1.5720639	0.001742	CLIC4
0.593077	0.003049	142.54277	437.900238	1.5710289	0.015701	SLC35E2|SLC35E2B
0.092825	0.000129	244.209305	771.584045	1.5707994	0.004012	ALKBH5
0.095145	0.000126	246.305511	778.276855	1.57052	0.004205	SRRD
0.881262	0.015158	112.147621	336.552155	1.5700494	0.008778	PLEK2
0.687118	0.003945	137.302231	419.73407	1.5673813	0.007028	BCL2
0.000035	0.000002	1047.06042	3235.48999	1.5665312	0.002154	MAF1
0.	0.	14886.2861	42397.9219	1.5656912	0.00174	DDIT3
0.684597	0.004007	137.302231	418.777954	1.5642102	0.007305	FAM115C
0.216533	0.000357	203.333069	633.903625	1.5618827	0.00484	RCVRN
0.005952	0.000017	396.185028	1252.5094	1.556845	0.003159	PTPN21
0.595896	0.002573	147.78331	450.329712	1.5562671	0.006745	KAZN
0.988646	0.182556	73.3676	212.257324	1.5562312	0.021604	IQCA1
0.951608	0.039557	97.474098	286.834229	1.5560308	0.01146	GFM2
0.006774	0.000016	400.377472	1264.93884	1.5553289	0.003275	SKIL
0.046131	0.000048	304.999603	956.114075	1.5542283	0.004456	ZNF134
0.00849	0.000021	372.078552	1171.23975	1.5540853	0.003295	IFT43
0.001082	0.000005	569.122986	1793.66992	1.553846	0.002864	SLC7A1
0.000006	0.000001	1708.41699	5189.78711	1.5536752	0.002091	MYL6B
0.660525	0.003632	140.446548	425.470764	1.5520336	0.00725	SPDYA
0.568446	0.002317	150.927643	458.934753	1.5514325	0.006786	BCL2L11
0.004737	0.000015	409.810455	1291.71008	1.5506754	0.003178	FBXL5

0.967225	0.054995	92.233551	269.624176	1.5497197	0.012304	HNRNPCL1|LOC101060301|LOC649330|LOC440563
0.776208	0.007293	126.821136	379.577271	1.549104	0.008264	C21orf7
0.000001	0.	10053.457	28482.6367	1.5485854	0.001977	SOX4
0.68808	0.004291	137.302231	413.997375	1.5482455	0.007556	FRMD3
0.000003	0.000001	2381.30273	7030.30664	1.5479679	0.002067	BMP4
0.001884	0.000009	477.9375	1502.05518	1.5457285	0.003024	ANO10
0.002238	0.00001	464.312103	1459.03003	1.5454734	0.003066	HNMT
0.68779	0.007384	137.302231	413.04126	1.5450304	0.012591	LY6E
0.021821	0.000041	317.576904	988.621948	1.5435146	0.003788	PDCD6
0.12174	0.000186	231.631989	717.08551	1.543069	0.004752	PYGO1
0.000002	0.000001	3061.52515	8943.49121	1.5424401	0.002068	LACTB
0.123256	0.000191	230.583893	713.261108	1.5420806	0.004758	PKN1
0.034423	0.000059	293.470398	912.132812	1.5418394	0.00401	ADPRM
0.001311	0.000008	508.332672	1592.88599	1.5416065	0.003001	LTF
0.000008	0.000001	1576.35535	4770.05322	1.541311	0.002238	PIP5K1A|PIP5K1P1
0.000126	0.000002	817.524719	2503.10669	1.5403283	0.002633	MRPL12
0.957659	0.047043	95.377884	277.273071	1.5402545	0.012285	B4GALT7
0.993911	0.205841	72.319489	206.52063	1.5399784	0.019881	STAG1
0.995409	0.217697	72.319489	206.52063	1.5399784	0.024799	RBM41
0.000041	0.000002	1006.1842	3053.82837	1.5392528	0.002459	FAM133B|FAM133DP|FAM133CP
0.230837	0.000427	200.188736	613.825195	1.5392153	0.005425	KIAA1671
0.000002	0.000001	2720.88989	7940.52734	1.5392103	0.00212	LTA4H
0.776491	0.007639	126.821136	376.708923	1.5385564	0.008621	C2orf40
0.000103	0.000002	819.620911	2503.10669	1.5367455	0.002618	TSSC4
0.910738	0.023969	106.907074	311.693176	1.5366088	0.010704	KIF13A
0.000003	0.000001	2218.84595	6522.60986	1.5352048	0.002204	TBPL1
0.000002	0.000001	2869.72119	8344.96387	1.5347745	0.002153	STC2
0.001086	0.000007	525.102417	1636.86731	1.5341501	0.003063	GPR176
0.044064	0.000074	280.893097	868.15155	1.5338674	0.004276	NUP37
0.000001	0.	8242.32617	23069.1191	1.5331033	0.002048	SESN2
0.017037	0.000036	331.202301	1023.99817	1.5329706	0.003871	MFSD8
0.000002	0.000001	2712.50488	7876.46777	1.5318225	0.002194	C20orf111
0.036163	0.000055	299.759064	925.518433	1.5317976	0.004375	PSMF1
0.978305	0.088477	85.944901	246.677429	1.5309679	0.015354	USP16
0.986767	0.127576	80.704361	230.423492	1.5296572	0.01819	ARMCX5
0.067422	0.000106	260.979034	803.135803	1.5284503	0.004662	TEX264
0.	0.	10780.8447	30064.0508	1.5266597	0.002093	PDRG1
0.002867	0.000012	445.446136	1381.58484	1.5265897	0.00342	TAF1A
0.026012	0.000083	308.143921	947.509033	1.5254587	0.005392	TGM3
0.033062	0.00006	295.56662	908.30835	1.5249547	0.004303	MAP4K5
0.941367	0.044416	97.474098	280.141418	1.5232009	0.01412	PLXNA1
0.801271	0.009683	123.676811	362.367218	1.5230164	0.009479	ARAF
0.000011	0.000001	1433.8125	4299.64502	1.5218986	0.002481	RPP14
0.000538	0.000005	594.277588	1830.00232	1.5216784	0.003093	N4BP2L1
0.998591	0.338937	64.982735	181.661667	1.5210569	0.022035	CPXM2
0.000006	0.000001	1773.39966	5254.80273	1.5206193	0.002419	LCMT1
0.768011	0.00788	127.869247	374.796722	1.5188806	0.009275	C20orf27

0.964825	0.067524	93.281662	266.755829	1.5185458	0.01844	GRINA
0.081479	0.000095	297.662842	910.220581	1.5175161	0.008465	FAM35A
0.000171	0.000003	737.868408	2237.30688	1.5156463	0.002966	CEBPG
0.518749	0.00224	155.120071	460.846985	1.5155078	0.023398	LRP6
0.002214	0.00001	466.408325	1435.1272	1.5152095	0.003517	KIAA1324
0.000003	0.000001	2468.29565	7109.66406	1.5146064	0.002422	RNF144B
0.01365	0.000032	343.779602	1048.85718	1.5129052	0.004145	GTF3C5
0.036068	0.000013	436.013184	1339.51575	1.5128419	0.007282	TAF12
0.000947	0.000007	537.679688	1651.20898	1.5126723	0.003344	SNAP47
0.000002	0.000001	2953.57007	8453.00391	1.5118749	0.002398	CDKN1B
0.00002	0.000001	1206.37292	3588.29614	1.5105175	0.002698	CLCN4
0.750627	0.007408	129.965469	378.621155	1.5081059	0.009538	PSMB10
0.01704	0.000038	331.202301	1006.78809	1.5077786	0.004334	DNASE2
0.000344	0.000004	645.634888	1960.98987	1.5060048	0.003231	C16orf87
0.000002	0.000001	2593.02051	7398.41064	1.5044813	0.002498	CCNL1
0.178409	0.000343	212.766037	638.684204	1.5031675	0.006021	FAM96A
0.000003	0.000001	2199.97998	6334.25537	1.5029132	0.002558	PRPF19
0.824911	0.012583	120.532486	347.069397	1.5024615	0.010643	ACOT8
0.002749	0.000018	448.590485	1367.24304	1.5014479	0.004514	COMMD2
0.000022	0.000001	1178.0741	3476.43066	1.4993401	0.002853	RPL9
0.809289	0.011394	122.6287	352.806091	1.4988633	0.01059	GEMIN8
0.000025	0.000001	1137.19775	3352.13599	1.4980914	0.002891	NT5C2
0.00016	0.000003	749.397644	2240.17529	1.4958732	0.003236	LOC100129034
0.010085	0.000028	361.597473	1091.8822	1.4957706	0.004354	LOC285629
0.000013	0.000001	1355.20435	3989.86401	1.4947236	0.002838	NAA50
0.899414	0.025301	109.003296	308.824829	1.4940279	0.012444	UBN1
0.000001	0.	5440.73145	14967.0098	1.4939754	0.002493	PSAT1
0.000007	0.000001	1664.39636	4863.75195	1.4921632	0.002783	DDB1
0.005627	0.000019	398.28125	1203.74756	1.4917761	0.004207	RCAN1
0.000004	0.000001	2124.51611	6087.57812	1.4915167	0.002706	CARS
0.002488	0.000011	465.360199	1408.35596	1.4913293	0.003982	BASP1
0.000005	0.000001	1852.00781	5359.97559	1.4902778	0.002766	CHD2
0.000025	0.000001	1145.58264	3357.87256	1.489929	0.002999	DIDO1
0.295183	0.000272	224.295242	669.279846	1.4899181	0.025562	WAC
0.000394	0.000005	628.865173	1891.1936	1.4895797	0.003512	CCNG1
0.000059	0.000002	927.576111	2723.96899	1.4888919	0.003161	NKIRAS2
0.013407	0.000034	344.827728	1034.51538	1.4878914	0.00463	EXOSC2
0.186297	0.000384	210.66983	625.298584	1.4877066	0.006498	GTF2H4
0.341708	0.000981	180.274673	529.687195	1.4866401	0.00748	KIF1C
0.001002	0.000007	532.439148	1604.35938	1.4853104	0.003803	KPNA2
0.000001	0.	8166.86182	22089.1035	1.4848629	0.002559	PPIB
0.278494	0.000683	191.803864	564.1073	1.4826284	0.007493	ZKSCAN2
0.99805	0.332745	67.078949	182.617783	1.4823442	0.024379	previous version conserved probe
0.887441	0.023535	111.09951	312.649292	1.4819829	0.012777	ERRFI1
0.399417	0.001345	171.889801	501.959869	1.4819128	0.00798	ZNF518B
0.061004	0.000012	464.312103	1394.97046	1.4808204	0.012861	CETN2
0.497287	0.002184	159.3125	462.759216	1.4806763	0.008631	GLTP
0.235792	0.00055	199.140625	586.0979	1.480372	0.007038	CD14
0.004406	0.00001	483.17804	1450.42505	1.4796786	0.004693	SPG20
0.000003	0.000001	2226.18262	6300.7915	1.4785011	0.002904	GNL2

0.001188	0.000008	517.765625	1552.72925	1.4783971	0.003973	ZSWIM6
0.000856	0.000006	585.8927	1753.51318	1.4781754	0.00397	NCOA5
0.149944	0.000302	221.150909	653.981995	1.4781105	0.006515	IKBIP
0.586393	0.004808	141.494659	406.34848	1.4762718	0.032823	CPO
0.000026	0.000002	1121.4762	3255.56836	1.4762311	0.003206	previous version conserved probe
0.002362	0.000012	460.119659	1377.76038	1.4759888	0.004219	FRMD6
0.532416	0.002641	155.120071	448.41748	1.4759369	0.009041	DESI1
0.055531	0.000084	284.037415	844.248718	1.4757309	0.006203	SEC16A
0.000007	0.000001	1675.92566	4837.93701	1.4745656	0.003013	CRKL
0.541349	0.003318	154.07196	444.593048	1.4739188	0.010558	ABHD3
0.019826	0.000047	322.817444	958.982422	1.4738063	0.005125	PPM1D
0.827877	0.017104	117.388161	329.859344	1.4722063	0.014321	CCDC92
0.143549	0.000292	223.247131	657.806458	1.4720723	0.006641	PCK2
0.000014	0.000001	1339.48279	3874.17407	1.4693286	0.003198	SNAPC1
0.00651	0.000022	388.848297	1155.94189	1.469253	0.004718	CASP2
0.07381	0.000103	273.556335	808.872498	1.4683137	0.006963	OR51Q1
0.000127	0.000003	783.985229	2293.71753	1.4681163	0.003688	ME3
0.491668	0.003471	160.360611	461.803101	1.467645	0.020461	YAF2
0.000001	0.	7745.52246	20687.4395	1.4675501	0.002778	BAMBI
0.006096	0.00002	402.473694	1196.09863	1.4669433	0.004805	RASA1
0.000108	0.000003	810.187927	2362.55786	1.4664026	0.003627	GAMT
0.003667	0.000005	649.827332	1913.1842	1.4597371	0.005627	ASB8
0.001265	0.000009	511.47699	1513.52856	1.4591501	0.004356	BCL2L1
0.226021	0.000566	201.236847	583.229553	1.4572027	0.007722	KLHL28
0.000014	0.000001	1344.72327	3855.05176	1.4565779	0.003391	BHLHB9
0.000004	0.000001	2049.05225	5751.98193	1.4562873	0.003201	SIRT7
0.002673	0.000013	450.686676	1330.91077	1.4559606	0.004666	GORAB
0.000071	0.000002	887.747986	2552.82446	1.4544859	0.003739	NAT14
0.000351	0.000005	642.49054	1884.50085	1.4534443	0.004115	RHOB
0.000334	0.000002	1143.48645	3267.04175	1.4531908	0.00451	TADA3
0.993217	0.209811	76.511925	205.564514	1.4524101	0.022854	ANP32E
0.77635	0.011208	126.821136	353.762207	1.4511656	0.01245	previous version conserved probe
0.328348	0.001057	182.370895	522.994385	1.4504958	0.008663	CEP63
0.88126	0.025301	112.147621	308.824829	1.4504947	0.014363	LINC00341
0.586718	0.003837	148.831421	421.646301	1.4502729	0.01051	TROVE2
0.006096	0.000022	393.04071	1152.11743	1.4484499	0.005156	SNX16
0.422675	0.001734	168.745483	480.925354	1.4482885	0.009386	previous version conserved probe
0.422673	0.001734	168.745483	480.925354	1.4482885	0.009388	EHBP1L1
0.974544	0.106028	88.041122	238.072403	1.4482388	0.020398	C10orf111
0.994738	0.240785	74.41571	198.871719	1.447775	0.024149	PIP5KL1
0.824877	0.01596	120.532486	333.683807	1.4477713	0.013365	LOC100507855
0.998055	0.364576	67.078949	177.837219	1.4465109	0.027828	TMPO
0.000002	0.000001	3060.47705	8360.26172	1.4441952	0.003256	EPC2
0.000027	0.000002	1119.38	3177.16699	1.4439239	0.003725	ZNF638

0.245947	0.000676	197.044418	565.063416	1.443714	0.008336	ASCC1
0.019448	0.000051	323.86554	942.728455	1.4435533	0.005861	BCO2
0.31532	0.001213	184.467102	526.818848	1.4435028	0.009929	EIF2AK1
0.000165	0.00001	743.109009	2145.52002	1.4430121	0.006181	ENTPD7
0.068913	0.00013	265.171478	770.62793	1.4428615	0.007095	TPRA1
0.000001	0.	4819.20312	12931.4424	1.4420131	0.003193	IL8
0.000886	0.000008	543.968323	1590.0177	1.4415724	0.004608	NMNAT1
0.000208	0.000004	711.66571	2058.51367	1.4415072	0.00422	CBWD1|CBWD3|CBWD2|CBWD5|CBWD7|CBWD6
0.12239	0.00006	313.38446	908.30835	1.4374497	0.016378	TBC1D12
0.123216	0.000288	230.583893	664.499268	1.4371026	0.007717	PPM1A
0.000803	0.000007	553.401306	1610.09607	1.434918	0.00472	NUP54
0.00372	0.000017	426.5802	1241.03601	1.4343371	0.005282	TDP2
0.967188	0.086721	92.233551	247.633545	1.434314	0.019495	MRPL2
0.056742	0.000124	269.363892	778.276855	1.4340914	0.006938	PRPF4B
0.969683	0.16518	81.752472	217.037888	1.4301986	0.033031	SAP30L
0.50597	0.00557	158.264389	443.636932	1.429705	0.018241	BCCIP
0.930838	0.047962	102.714638	276.316956	1.4285362	0.01742	IFI6
0.000021	0.000001	1189.60327	3340.6626	1.4280476	0.003961	UCK2
0.015136	0.000015	461.167786	1334.73523	1.4270313	0.00825	PHF20L1
0.000007	0.000001	1981.97327	5468.97217	1.4264758	0.003803	SLTM
0.951108	0.065614	97.474098	261.019135	1.4256993	0.018836	ZCCHC7
0.256513	0.000776	194.948196	551.677795	1.4254298	0.009112	MITD1
0.001884	0.000012	477.9375	1381.58484	1.4253418	0.005232	LAP3
0.000794	0.000007	575.411621	1662.68237	1.4251075	0.004985	ARHGAP12
0.613741	0.005909	145.687088	404.436249	1.4250143	0.014002	ATG12
0.001463	0.00001	498.899689	1440.86389	1.4241071	0.005168	FUBP1
0.099095	0.000228	241.064972	689.358215	1.423204	0.00778	NUP98
0.000098	0.000003	826.957642	2339.61108	1.4231231	0.004403	PPID
0.430618	0.002003	167.697372	469.451996	1.4229034	0.010531	PHACTR2
0.463293	0.002354	163.504944	457.022522	1.4228594	0.010785	LOC100133050
0.916006	0.041119	105.858963	283.965881	1.4221433	0.017264	KLHL24
0.000003	0.000001	2260.77026	6147.81348	1.4200777	0.003748	PPFIA1
0.000007	0.000001	1666.49268	4635.24072	1.4179233	0.003941	KLF5
0.000003	0.000001	2490.30591	6710.0083	1.4175735	0.003804	FAM214A
0.012472	0.00004	349.020142	997.226929	1.4152645	0.006391	LARP4
0.000002	0.000001	2787.96875	7472.03125	1.4148744	0.003765	SRSF9
0.817132	0.017405	121.580597	328.903229	1.4144518	0.015192	MOCS2
0.000049	0.000002	962.163696	2679.98779	1.4142924	0.004624	MAP3K9
0.0015	0.000011	496.803467	1423.65381	1.4128593	0.005452	previous version conserved probe
0.961792	0.083307	94.329773	249.545761	1.4122835	0.020554	OR6V1
0.007546	0.000029	379.415314	1082.32117	1.4112024	0.006223	PTGES2
0.000002	0.000001	3219.78955	8594.50879	1.4111198	0.003786	DDX1
0.043461	0.000107	281.941193	802.179688	1.410833	0.007409	FUNDC2|FUNDC2P2
0.566766	0.00552	146.735199	403.480133	1.4107496	0.032349	C15orf38
0.000314	0.000005	656.115967	1868.24683	1.4102928	0.00502	MED13
0.557139	0.004172	150.927643	415.909607	1.4098142	0.016814	PEX10

0.000001	0.000001	3531.07788	9406.25	1.4095288	0.003789	GLO1
0.112915	0.000277	234.776321	664.499268	1.4094521	0.00843	CCL18|LOC101060271
0.013897	0.000001	1931.66406	5280.61768	1.4093621	0.014873	KIF1B
0.000017	0.000001	1269.25952	3519.45581	1.4092469	0.004276	IP6K2
0.101852	0.000246	240.016861	679.797119	1.4091321	0.008337	CINP
0.897841	0.046143	104.81086	278.229187	1.4089794	0.022358	ARL16
0.438663	0.002211	166.649261	461.803101	1.4087492	0.01126	WDR81
0.993837	0.255216	75.463821	196.003387	1.4079724	0.02776	RNF141
0.091927	0.000095	289.277954	821.302002	1.4074448	0.011876	CCDC137
0.951975	0.080052	95.377884	251.457993	1.4066536	0.022358	TMEM170A
0.000063	0.000002	913.950684	2540.39502	1.4064789	0.004645	TGM2
0.070103	0.000169	257.834717	730.47113	1.4061003	0.008005	TCF4
0.000004	0.000001	2149.67065	5811.26123	1.4052025	0.00422	DAPK3
0.000106	0.000003	823.813354	2302.32275	1.4043932	0.004832	PXK
0.296645	0.001068	187.611435	522.038269	1.4043725	0.010325	RPS2P32|RPS2
0.006833	0.000083	385.703949	1093.79443	1.4018127	0.010246	CDC27
0.001402	0.000001	1483.07361	4095.03638	1.4017674	0.007436	PSMD11
0.00025	0.000004	692.799744	1954.29712	1.4012956	0.005142	TXNRD2
0.000109	0.000003	828.005798	2308.05933	1.4010179	0.004928	previous version conserved probe
0.733181	0.010669	132.061676	356.630554	1.4004099	0.014709	COG7
0.062592	0.000145	267.2677	753.417847	1.3975217	0.00841	WDR47
0.203071	0.000615	206.477386	574.624573	1.3962394	0.009877	MANBAL
0.430625	0.002239	167.697372	460.846985	1.3961284	0.01182	SYT1
0.000058	0.000002	929.672302	2563.3418	1.3960827	0.004852	CLN3
0.000026	0.000002	1128.81299	3097.80957	1.3954651	0.004661	DRAM1
0.810398	0.026727	115.291946	305.956482	1.3951903	0.025739	FAM151B
0.00001	0.000001	1454.77466	3997.51294	1.3949963	0.00446	MCFD2
0.391853	0.001863	172.937912	475.18869	1.3932609	0.011638	CSTF2
0.000032	0.000002	1085.84045	2970.64648	1.3913637	0.004842	EIF3K
0.137428	0.000375	225.343338	628.166931	1.3910037	0.009435	SHCBP1
0.000002	0.000001	2716.69751	7160.33838	1.3900121	0.004242	RGS3
0.000015	0.000001	1316.42432	3600.72559	1.389298	0.004659	SNX25
0.86746	0.030976	114.243835	301.175934	1.387272	0.01969	RASA4B|RASA4CP
0.000004	0.000041	2479.82495	6550.3374	1.3872631	0.017634	PLP2
0.000007	0.000001	1649.7229	4495.64844	1.3863035	0.004568	MRPS18C
0.221203	0.000767	202.284958	558.370605	1.3861901	0.010851	LRRC27
0.00052	0.000003	830.10199	2288.93701	1.3850216	0.006229	SARNP
0.000001	0.000001	3782.62378	9896.73633	1.3842331	0.004248	ZMAT3
0.000116	0.000003	797.610657	2203.84277	1.3841513	0.005325	RRP12
0.704616	0.010329	134.157898	358.542755	1.3837379	0.017019	MTR
0.001812	0.000013	481.081848	1350.98914	1.3836644	0.00632	PPIL4
0.463292	0.002781	163.504944	444.593048	1.3829528	0.012798	MINPP1
0.000736	0.000001	1579.49963	4298.68896	1.3816894	0.00736	UBE2NL
0.000001	0.	6371.45215	16056.9795	1.3816419	0.004195	TARS
0.058149	0.000131	275.652557	768.715698	1.3812204	0.00922	IQCK
0.000001	0.	8806.20801	22108.2246	1.3789678	0.004212	PLK2
0.256551	0.000941	194.948196	533.511658	1.3769699	0.011265	ZMIZ1
0.974236	0.146393	86.993012	222.774582	1.3758025	0.028601	SMIM11

0.975281	0.129726	89.089226	228.511261	1.3757956	0.025394	BICD1
0.935379	0.063065	101.666534	262.931366	1.3748055	0.021852	GTF3C3
0.000043	0.000002	995.703125	2693.37329	1.3735084	0.005307	NEXN
0.123407	0.000351	230.583893	635.815857	1.3732413	0.010043	TMEM237
0.847277	0.026247	117.388161	306.912598	1.371946	0.018804	THAP4
0.008784	0.000035	369.98233	1026.86646	1.3716066	0.007551	TMEM9B
0.12798	0.000363	229.535782	631.991394	1.3715007	0.010268	DTNA
0.000089	0.000003	844.775513	2305.19092	1.3711028	0.005578	USP53
0.000003	0.000001	2585.68384	6733.91113	1.3706181	0.004707	EFNB2
0.20475	0.000507	216.958481	594.702942	1.3699626	0.013975	LOC101060409|NUDT17
0.000004	0.000003	2016.56091	5351.37012	1.3697176	0.007648	NECAP2
0.009908	0.000039	362.645569	1004.87585	1.3693729	0.007708	CDK8
0.989103	0.229213	80.704361	204.608414	1.3691503	0.040867	RAI1
0.000001	0.	10095.3818	25166.834	1.3678174	0.004407	BAG3
0.014104	0.000006	611.047302	1693.27795	1.3653751	0.01231	VAMP3
0.981469	0.161894	85.944901	217.994003	1.3639865	0.027737	KIAA1967
0.982772	0.171925	84.896797	215.125656	1.3637964	0.028436	AMACR|C1QTNF3-AMACR
0.000115	0.000003	804.947388	2190.45728	1.3622174	0.005901	AUH
0.000004	0.000001	1996.64685	5275.8374	1.3620769	0.004992	SEC11C
0.391841	0.00213	172.937912	464.671417	1.3608686	0.013379	SECISBP2
0.281789	0.001152	190.755768	515.345459	1.3601921	0.012614	KDM4C
0.235772	0.000886	199.140625	539.248352	1.3597975	0.01194	NEXN
0.245967	0.000951	197.044418	532.555542	1.3579613	0.01213	TCEAL1
0.000002	0.000006	2615.03101	6740.604	1.3564091	0.009973	SRSF1
0.267473	0.001103	192.851974	519.169922	1.3541217	0.012554	CAPN1
0.879759	0.038272	112.147621	287.790344	1.3524053	0.021771	FAM49B
0.001467	0.000019	502.044006	1378.71643	1.3515836	0.008665	MCM4
0.793124	0.019513	124.724922	323.166565	1.350898	0.019392	MCCC1
0.000457	0.000006	612.095398	1678.93628	1.3502377	0.006739	RAB3GAP2
0.007566	0.000034	379.415314	1037.38379	1.3491167	0.008266	DYNC2LI1
0.056648	0.001559	268.315796	731.427246	1.3476439	0.029829	SLC39A6
0.031116	0.000099	298.710938	814.609192	1.3473154	0.009489	CRY1
0.235789	0.000931	199.140625	534.467773	1.3469099	0.01262	PSENEN
0.002673	0.000017	450.686676	1233.38708	1.3463791	0.007725	DUSP11
0.117967	0.000369	232.680099	630.079163	1.3462363	0.011234	BPNT1
0.000045	0.000002	981.029602	2605.41089	1.3452298	0.00608	BCAR3
0.29661	0.00136	187.611435	501.003754	1.3448497	0.013369	DGCR6|DGCR6L
0.027102	0.000089	306.047699	832.77533	1.3438233	0.009515	CNOT2
0.000649	0.000001	1609.89478	4269.04932	1.3437157	0.008655	TNKS2
0.96769	0.122285	92.233551	231.379608	1.3426058	0.027846	FKBP15
0.471673	0.003439	162.456833	429.295197	1.3421211	0.015325	AK2
0.997742	0.441994	68.12706	167.319962	1.3417788	0.039868	ACRC
0.022471	0.000077	316.528778	859.546509	1.3405141	0.009488	PLA2G16
0.113564	0.000204	259.930939	704.656067	1.340198	0.014944	CPEB3

0.000084	0.000003	860.497131	2296.58594	1.3397037	0.006445	GUSBP3|LOC101060519|LOC100653061|GUSBP4|GUSBP2|GUSBP1|SMA5|LOC100170939|GUSBP9|GUSB
0.893439	0.045262	110.051399	279.185303	1.3390784	0.02309	SP100
0.995396	0.345224	73.3676	180.705551	1.3388418	0.036833	AFP
0.149945	0.000555	221.150909	593.746826	1.3382635	0.012609	DLG1
0.25118	0.001068	195.996307	522.038269	1.3372772	0.013331	ZMYM4
0.000664	0.000009	572.267273	1555.59753	1.3371248	0.007574	MRPL23
0.027603	0.000094	304.999603	826.08252	1.3369586	0.009869	BAG5
0.084797	0.000232	254.690384	688.4021	1.3367678	0.01182	MTAP
0.000001	0.	9394.19727	22894.1504	1.3364467	0.005127	ITGB1
0.409072	0.002104	176.082245	465.627533	1.336193	0.028274	SIGMAR1
0.34858	0.001887	179.226562	474.232574	1.3355343	0.014474	RANBP9
0.935383	0.073903	101.666534	255.282455	1.3349256	0.025602	CYP2R1
0.993217	0.297455	76.511925	188.354462	1.3343011	0.035617	TADA2A
0.008994	0.000111	368.934204	998.183044	1.3337401	0.013608	C11orf49
0.010441	0.000045	359.501251	972.367981	1.3335142	0.009116	SHB
0.000054	0.000002	942.249634	2488.76489	1.3330666	0.006495	TNFRSF14
0.984446	0.201661	83.848686	207.476746	1.3328143	0.032461	COMMD8
0.894692	0.066919	103.762749	260.063019	1.3305443	0.033077	DLC1
0.000264	0.000005	679.174377	1828.09009	1.3291131	0.007204	TOPORS
0.998802	0.544082	63.934624	154.890472	1.3288498	0.048305	MYO19
0.994882	0.412734	70.223274	171.144409	1.3286029	0.045986	ABCB5
0.002427	0.000017	458.023438	1237.21155	1.3276148	0.008356	EMP3
0.000002	0.000001	2712.50488	6840.04004	1.3252428	0.005768	POLR2K
0.000001	0.	9858.50977	23834.9668	1.3247648	0.005414	EXT1
0.000004	0.000001	2054.29272	5282.53027	1.3244846	0.005947	SOCS5
0.043737	0.000138	284.037415	762.022888	1.3234297	0.011132	PTGER1
0.532415	0.005094	155.120071	402.524017	1.3223836	0.017395	SPG20OS
0.00004	0.000002	1011.4248	2639.83081	1.322194	0.006731	HNRNPU
0.996961	0.419905	70.223274	170.188309	1.3210353	0.041401	SARS2
0.000047	0.000002	973.692871	2545.17554	1.3207045	0.006834	ZNF10
0.598223	0.00953	141.494659	363.323334	1.3206485	0.044296	PAF1
0.00001	0.000001	2299.55029	5842.81299	1.3198186	0.006449	PSMB3
0.000002	0.000001	2652.7627	6666.02734	1.3196234	0.005935	DUSP22
0.000165	0.000004	743.109009	1975.33167	1.3192484	0.007349	TRNAU1AP
0.190398	0.000761	209.621719	553.590027	1.3190735	0.013693	SLC46A3
0.000001	0.	4091.81592	10226.5957	1.3183791	0.005783	PRDX2
0.160081	0.000604	218.006592	576.536804	1.3176681	0.013373	SP3
0.000001	0.	4225.97363	10545.9385	1.3175302	0.005793	ZNF655
0.	0.	13623.3154	32587.2344	1.3162711	0.005599	BRD2
0.742003	0.016235	131.013565	332.727692	1.3161395	0.02105	HEXDC
0.723708	0.014649	133.109787	338.464386	1.3156105	0.020857	previous version conserved probe
0.017748	0.00007	329.106079	878.668823	1.3149675	0.010402	RPS6KB1
0.007178	0.000036	382.559631	1022.08594	1.3146983	0.00964	ZMYM5

0.000001	0.	7372.39551	17661.3379	1.3143506	0.005742	GTF2H1
0.998746	0.561322	63.934624	152.978241	1.3120225	0.048246	MAST4
0.861376	0.038109	115.291946	287.790344	1.3100761	0.024641	RNF167
0.025585	0.000092	311.288239	827.994751	1.309886	0.011107	ANKRD13C
0.784837	0.021896	125.773026	316.473755	1.3089878	0.0226	CARKD
0.012407	0.000062	403.52179	1074.67224	1.3089066	0.015033	REPS1
0.76549	0.020753	126.821136	319.342102	1.30883	0.024283	LURAP1L
0.004952	0.000028	407.714233	1086.14551	1.3087476	0.009622	SERAC1
0.291279	0.001524	188.659546	491.442627	1.3084136	0.015668	HLX
0.588362	0.007948	146.735199	374.796722	1.3082082	0.026336	PLEC
0.07012	0.000239	257.834717	683.621521	1.3075809	0.012423	COMMD2
0.000001	0.	4408.34473	10892.0518	1.3075001	0.006065	EIF1
0.000002	0.000001	3031.13013	7542.78369	1.3074929	0.006217	MTCH1
0.000006	0.000033	1758.7262	4525.2876	1.3069957	0.019775	ELF1
0.000038	0.000002	1022.95398	2640.78711	1.306878	0.007218	TRRAP
0.559369	0.006265	151.975739	389.138428	1.3067058	0.018949	previous version conserved probe
0.369745	0.002385	176.082245	456.066406	1.306165	0.016666	GFRA1
0.182915	0.000507	226.391449	594.702942	1.3046524	0.018886	KCTD11
0.000051	0.000003	954.826904	2471.55493	1.3041234	0.007421	IKBKAP
0.01293	0.000057	346.92395	919.781738	1.3041151	0.010617	TYW5
0.023605	0.000091	313.38446	829.906982	1.3032818	0.011265	XIAP
0.00566	0.000032	398.28125	1054.59375	1.3010297	0.010066	LMCD1
0.947254	0.112639	96.425987	235.204056	1.3006206	0.033735	RNF41
0.000926	0.000011	539.77594	1430.34668	1.3003087	0.008887	ZRANB1
0.002181	0.000016	474.793182	1258.24609	1.3001785	0.009492	previous version conserved probe
0.391977	0.002745	172.937912	445.549164	1.300049	0.017399	SETD2
0.000003	0.000001	2242.95239	5635.33643	1.2999388	0.006622	STXBP5
0.541343	0.005901	154.07196	392.962891	1.2993345	0.019304	PBX4
0.207474	0.000931	205.429276	534.467773	1.2991993	0.015167	ANKHD1-EIF4EBP3
0.000048	0.000002	968.452332	2495.45776	1.2987126	0.007589	UHRF1BP1
0.000237	0.000005	694.895996	1828.09009	1.2970956	0.008323	BRK1
0.008079	0.000041	375.22287	990.53418	1.2970408	0.010541	FHIT
0.000002	0.000001	2675.82129	6616.30908	1.2958037	0.006645	HEXB
0.002007	0.000008	586.940796	1548.90479	1.2945065	0.010462	CREBBP
0.99233	0.314699	77.560036	185.48613	1.293943	0.040642	ZNF260
0.172974	0.000724	214.862259	558.370605	1.2936257	0.01521	TOMM22
0.000025	0.000002	1140.34216	2915.19165	1.2935114	0.007535	GRPEL2
0.	0.	11424.3838	26966.2402	1.2926543	0.006293	SLC25A3
0.009889	0.000012	516.717529	1361.50647	1.2920657	0.013964	USP43
0.000065	0.000003	903.469604	2327.18164	1.291341	0.007979	BZW1
0.000017	0.000001	1327.95361	3392.29272	1.2910519	0.007501	PRDX2
0.00136	0.000013	505.188324	1329.95459	1.2907537	0.009528	THAP1
0.000141	0.000004	767.215454	1996.36609	1.2904442	0.00834	SLC1A4
0.007676	0.00004	378.367188	994.358643	1.2902936	0.010825	LLPH
0.000002	0.000001	3292.10913	8083.94434	1.2892571	0.006743	TNFRSF19

0.00246	0.000019	456.975342	1201.83533	1.2891353	0.00999	previous version conserved probe
0.000002	0.000001	3059.42896	7511.23193	1.2878887	0.006822	SPAG7
0.939676	0.093971	100.618423	243.809082	1.287758	0.031185	YTHDC1
0.099877	0.000357	243.161194	633.903625	1.2873816	0.014586	PRKY
0.008946	0.000046	368.934204	966.631287	1.2860012	0.011178	PCCB
0.00027	0.000006	674.981934	1764.98657	1.2851705	0.008856	NF2
0.980906	0.21003	85.944901	205.564514	1.2846827	0.038022	ZNF322
0.004441	0.000028	415.050995	1088.05774	1.2846192	0.010657	MRPS15
0.052972	0.000021	447.542358	1173.15198	1.2843478	0.026399	RBM34
0.661047	0.012568	139.398438	348.025513	1.2836806	0.025066	ITGAV
0.784866	0.024721	125.773026	310.737061	1.2835509	0.025429	LARP1
0.043195	0.000163	281.941193	736.207825	1.2832793	0.013104	NLGN3
0.000658	0.000009	573.315369	1501.09912	1.2831403	0.009422	NDUFAF4|NDUFAF4P1
0.012696	0.00006	347.972046	909.264465	1.2825127	0.011694	ZC3H7A
0.96179	0.135067	94.329773	226.59903	1.2819821	0.034174	CCDC124
0.000046	0.000002	985.222046	2505.97485	1.280991	0.008246	GPR89A|GPR89C|GPR89B
0.913656	0.07691	104.81086	253.370224	1.2808257	0.032563	BST1
0.000024	0.000002	1144.53455	2897.02563	1.2792291	0.00804	TSC22D2
0.000037	0.000002	1031.33887	2611.14746	1.278554	0.008237	BEX4
0.335019	0.002239	181.322784	460.846985	1.2762519	0.018498	ANKRD32
0.377011	0.002822	175.034134	443.636932	1.2753353	0.019124	ZNF513
0.010074	0.000003	843.727417	2158.90552	1.2746587	0.019449	SACS
0.302742	0.002148	186.563324	474.232574	1.2739679	0.019696	NMT1
0.000035	0.000002	1047.06042	2639.83081	1.2737478	0.008401	DDA1
0.000002	0.000001	3156.90308	7670.90283	1.2732383	0.007301	TPR
0.000008	0.000001	1581.59583	3993.68848	1.2728093	0.007851	NBPF15|NBPF14|NBPF11|NBPF8|LOC100288142|LOC101060202|NBPF24|NBPF16|LOC101060684|NBPF12|LOC101060362|LOC101060226|LOC100996575|LOC101060238
0.	0.	11489.3662	26733.9043	1.2725671	0.00693	UBC|UBA52
0.085111	0.000334	248.401733	641.552551	1.2720196	0.014914	ZBTB26
0.000053	0.000003	948.538269	2401.75854	1.27047	0.008709	MBTD1
0.216553	0.001115	203.333069	518.213806	1.2702413	0.017342	QRICH1
0.809283	0.030419	122.6287	299.263702	1.2699939	0.027169	UFM1
0.000001	0.000001	3522.69287	8528.53711	1.2695448	0.007372	DDB2
0.977463	0.197696	88.041122	208.432861	1.2686355	0.038756	GMPPA
0.541249	0.006758	154.07196	384.357849	1.268547	0.022054	EDEM2
0.130064	0.000555	228.487671	585.141785	1.2670516	0.016273	TRAM2
0.000002	0.000001	2821.5083	6831.43506	1.2663045	0.007615	PPP1R15B

0.958402	0.136216	95.377884	226.59903	1.2660405	0.036782	VAC14
0.678916	0.014159	138.350327	340.376617	1.2643324	0.02471	SETD9
0.011007	0.000057	356.356903	919.781738	1.2640117	0.012569	GTF2IRD2B|GTF2IRD2|GTF2IRD2P1
0.000001	0.	7808.40869	18054.3008	1.2637728	0.007305	NFE2L2
0.000002	0.000001	2604.5498	6312.26514	1.2633861	0.007775	INPPL1
0.925995	0.092089	102.714638	244.765198	1.2632975	0.034929	C2orf49
0.930845	0.092099	102.714638	244.765198	1.2632975	0.033543	BRPF1
0.001489	0.000002	1082.69617	2703.89062	1.2604219	0.013885	CA5A
0.000001	0.	8648.99219	19935.9336	1.2584924	0.007469	DNAJB1
0.000005	0.000001	1920.13489	4750.93066	1.2584354	0.008208	UFD1L
0.000024	0.000002	1145.58264	2857.82495	1.2583391	0.008876	HPS5
0.01965	0.000057	357.405029	918.825623	1.2580756	0.014997	NUAK2
0.000092	0.000004	838.486877	2122.57324	1.2579594	0.009503	EIF5
0.059109	0.00024	266.219574	682.665405	1.2568247	0.01526	RCOR3
0.000001	0.	4885.23389	11572.8047	1.2567758	0.007693	EIF3I
0.027061	0.000119	306.047699	784.969666	1.2559555	0.014124	FAM89B
0.000016	0.000002	1287.07739	3207.7627	1.2559106	0.008797	NBPF8|LOC101060684|LOC101060362|LOC100996575|NBPF15|NBPF14|NBPF11|LOC100288142|LOC101060202|NBPF24|NBPF16|NBPF12|LOC101060238
0.000449	0.000008	614.19165	1577.58813	1.2556632	0.010466	QSOX1
0.488673	0.005482	160.360611	397.743439	1.2549081	0.022457	CITED1
0.541342	0.007182	154.07196	380.533386	1.2546416	0.023294	SCRN1
0.002239	0.000018	480.033722	1232.43103	1.2545416	0.01182	STK17B
0.000002	0.000001	3242.8479	7774.16357	1.2538923	0.007994	ETS2
0.981469	0.231531	85.944901	200.783951	1.252897	0.042185	FOSL1
0.801214	0.030983	123.676811	298.307587	1.252516	0.028909	GZMM
0.000002	0.000001	2978.72461	7137.3916	1.2518776	0.008138	C16orf80
0.000009	0.000001	1526.04614	3790.03613	1.2493162	0.008827	PQBP1
0.002428	0.000021	458.023438	1170.28357	1.2475319	0.012096	GNG11
0.550144	0.00776	153.023849	375.752838	1.2475115	0.024282	NRP2
0.000001	0.	5252.07227	12292.7588	1.2474427	0.008021	LAMTOR3
0.042255	0.000181	282.989319	720.909973	1.2465231	0.015405	MLL
0.000052	0.000003	951.682617	2370.20679	1.2460367	0.009768	TCEAL4
0.921004	0.097618	104.81086	246.677429	1.2446636	0.04266	B3GNT9
0.733097	0.022292	131.013565	315.517639	1.2422898	0.030479	CBL
0.000011	0.	6141.9165	14146.6631	1.2422779	0.009682	ARHGEF2
0.424297	0.004113	168.745483	416.865723	1.242124	0.023411	SEC61A2
0.194525	0.002598	208.573608	522.038269	1.2418335	0.030734	STK19
0.473215	0.008088	162.456833	399.65567	1.2416962	0.033696	ZNF623
0.163602	0.000841	216.958481	544.02887	1.2410675	0.018772	HIPK2
0.545273	0.008005	153.023849	373.840607	1.2404173	0.029403	HN1L

0.226508	0.001345	201.236847	501.959869	1.2400217	0.019989	previous version conserved probe
0.5959	0.010163	147.78331	359.498871	1.2393662	0.025769	TCF12
0.000013	0.000003	1374.07031	3385.59985	1.2388717	0.011738	CTSC
0.812458	0.036699	121.580597	289.702545	1.2379833	0.032156	MCTP1
0.00001	0.000001	1477.83313	3635.14575	1.2357895	0.00946	TMEM45A
0.	0.	13936.7002	31475.2754	1.2356966	0.008247	RPL4
0.847274	0.046142	117.388161	278.229187	1.2355117	0.032568	VPS16
0.000188	0.000005	724.243042	1822.35339	1.2345154	0.01101	CSDAP1|CSDA
0.921146	0.092089	104.81086	244.765198	1.2341513	0.036792	ZNF614
0.000006	0.000001	1807.9873	4421.07129	1.233558	0.009305	previous version conserved probe
0.019452	0.000097	323.86554	817.477539	1.2316753	0.015283	DNAJC4
0.001911	0.000019	476.889404	1204.70374	1.2312256	0.012836	ZC3HAV1
0.0293	0.000138	301.855286	761.066772	1.2306127	0.015949	ARID4A
0.568449	0.009229	150.927643	365.235565	1.2291513	0.026384	ZCCHC6
0.06863	0.000311	258.882812	650.157532	1.2278822	0.017662	TTC3
0.000005	0.000001	1944.24146	4707.90576	1.2274149	0.009503	HSPBAP1
0.973149	0.214018	89.089226	204.608414	1.2265459	0.0462	previous version conserved probe
0.642019	0.013686	142.54277	342.288818	1.2264231	0.028094	ATF4
0.084425	0.000388	249.449844	624.342468	1.2262036	0.018351	SVIL|SVILP1
0.002972	0.000026	443.349915	1114.82898	1.2244541	0.013662	UCHL3
0.001637	0.000018	489.466705	1230.5188	1.2243199	0.013118	NSMCE4A
0.632805	0.013613	143.590881	344.20105	1.2229549	0.028981	OAZ3
0.033064	0.000158	295.56662	740.988403	1.2223473	0.016747	CYCS
0.006504	0.000044	388.848297	976.192444	1.2220431	0.014594	PEX13
0.000001	0.000001	3546.79932	8310.54297	1.2217489	0.009271	CCDC59
0.001303	0.000015	509.380768	1277.36841	1.2207756	0.013145	CHCHD3
0.000969	0.000013	535.583496	1342.38416	1.2201272	0.012938	FLJ31306
0.000004	0.000001	2061.62964	4944.06592	1.2197843	0.00982	previous version conserved probe
0.000005	0.000001	1904.41333	4593.17188	1.2189738	0.009918	CD55
0.715254	0.021128	134.157898	318.385986	1.218568	0.030675	SH3TC1
0.006399	0.000044	389.896393	976.192444	1.2180192	0.014847	PHACTR4
0.000003	0.000001	2383.39893	5642.02881	1.2174352	0.009766	RTFDC1
0.000005	0.000001	2511.26807	5918.34619	1.2169332	0.010112	MRPS34
0.000001	0.	7738.18555	17305.6641	1.2167971	0.009164	EAPP
0.	0.	23231.3262	51651.1953	1.2166023	0.008986	HSPA1A
0.000025	0.000002	1134.05347	2746.91577	1.2161099	0.010867	OTUD7B
0.894081	0.078468	109.003296	252.414108	1.2156961	0.039447	FBXL20
0.000136	0.000005	772.455994	1911.27197	1.2156886	0.011829	PAXBP1
0.955184	0.152404	96.425987	220.86235	1.2156348	0.042965	DENND6A
0.000001	0.	9700.24512	21704.7461	1.2155342	0.009167	SYNPO

0.000336	0.000007	647.731079	1617.745	1.2153967	0.012427	ABRACL
0.861375	0.056085	115.291946	268.66806	1.2144703	0.036058	SLC30A6
0.000157	0.000005	750.44574	1857.72961	1.2135829	0.012035	C1GALT1
0.595891	0.011393	147.78331	352.806091	1.2132352	0.028712	TOMM34
0.190359	0.001164	209.621719	514.389343	1.212779	0.021753	ENOSF1
0.939721	0.124592	100.618423	230.423492	1.2114812	0.041823	PDE12
0.	0.	11018.7656	24565.4375	1.2110412	0.009333	SPHK1
0.000018	0.000002	1237.81628	2988.8125	1.2107791	0.010969	LAYN
0.000003	0.000001	5399.85547	12305.1875	1.2106935	0.01112	UGCG
0.085111	0.000422	248.401733	614.781311	1.2103299	0.019594	TRIP4
0.000238	0.000006	691.751648	1717.18079	1.2097141	0.012522	MAPKAPK5
0.000021	0.000002	1201.13245	2897.02563	1.2094638	0.011109	C2orf47
0.010087	0.000063	361.597473	899.703308	1.2093839	0.015997	FAM46C
0.112932	0.000593	234.776321	578.448975	1.2087373	0.020479	KLHL28
0.914903	0.095865	105.858963	242.852966	1.2083417	0.040572	DIMT1
0.000041	0.000003	1003.03992	2427.57349	1.2083148	0.011549	BIRC6
0.000006	0.000001	1795.41003	4318.76709	1.2079771	0.010524	AEN
0.000007	0.000001	1654.9635	3995.60059	1.2079552	0.010636	MVP
0.000001	0.000001	3907.34888	9056.3125	1.2072825	0.009879	FAM50A
0.000001	0.000001	3994.3418	9253.27148	1.2068755	0.009885	LAMTOR3
0.887525	0.072446	111.09951	256.238556	1.2068507	0.038517	KDM6A
0.00196	0.000005	756.734375	1863.46631	1.2065248	0.017109	RNF139
0.23671	0.001057	213.814148	522.994385	1.2064013	0.033693	RSF1
0.715266	0.022296	134.157898	315.517639	1.2059843	0.032277	FRMD3
0.215637	0.00136	205.429276	501.003754	1.2056208	0.023934	ANKMY2
0.	0.	12639.1416	27994.0625	1.20549	0.00956	KLHL21
0.899508	0.081667	109.003296	250.501877	1.2054237	0.039492	IPO11-LRRC70|IPO11
0.784811	0.03402	125.773026	293.527008	1.2043231	0.034501	ATP2B1
0.05926	0.000112	320.721222	794.530762	1.2039424	0.02621	STAT6
0.020996	0.000114	319.673126	791.662415	1.2034605	0.017475	HIST1H2BD
0.595855	0.011971	147.78331	349.937744	1.2018838	0.030099	PIBF1
0.341743	0.003172	180.274673	435.031891	1.2017165	0.02552	LOC101060769|MAFIP|TEKT4P2|LOC100233156|TEKT4
0.000005	0.000001	1897.07654	4523.37549	1.2012637	0.010799	NUP98
0.002583	0.000025	453.831024	1122.47791	1.2006982	0.015194	SURF2
0.000003	0.000001	2398.07251	5609.521	1.2002123	0.010602	previous version conserved probe
0.003213	0.000029	437.061279	1080.40894	1.1998389	0.015359	TEX30
0.000453	0.000008	621.528381	1535.51917	1.1996659	0.01365	SH2D4A
0.000113	0.000004	801.80304	1957.16553	1.1990528	0.012675	ARG2
0.003214	0.000029	437.061279	1079.45276	1.198564	0.01545	NDUFV3
0.930794	0.117276	102.714638	233.291824	1.1984453	0.043078	CHRNB1
0.000001	0.	6728.85693	14948.8428	1.1984147	0.010061	TCEB1
0.833468	0.050845	118.436272	273.448608	1.1978048	0.0391	ZNRF3
0.001538	0.000018	494.707245	1220.95764	1.1977721	0.014773	PPM1B
0.002748	0.000028	448.590485	1107.18005	1.1976411	0.01567	FAM118B
0.000001	0.000001	4275.23486	9819.29102	1.1970164	0.01037	CYR61

0.240835	0.001777	198.092514	479.013153	1.1964669	0.024325	SLC50A1
0.012469	0.000077	349.020142	860.502625	1.1960935	0.01728	previous version conserved probe
0.	0.	20834.3027	45794.0391	1.1960004	0.009932	C6orf48
0.505965	0.007759	158.264389	375.752838	1.1959644	0.029101	CNTRL
0.003407	0.00003	432.868835	1067.02332	1.1957484	0.015716	MAGI1
0.014419	0.000086	340.635284	839.46814	1.1956619	0.017523	TERF2
0.000132	0.000004	808.091736	1966.72656	1.1954201	0.013103	CWC15
0.000039	0.000002	1074.31128	2567.16626	1.1948667	0.01235	C3
0.138342	0.000717	229.535782	559.326721	1.1947259	0.023771	FBXO3
0.143572	0.00085	223.247131	543.072754	1.1946742	0.022527	EPS8L2
0.001086	0.000015	525.102417	1291.71008	1.1931384	0.014768	RCHY1
0.000007	0.000001	1680.11804	4012.81079	1.1929667	0.011405	INTS6
0.081509	0.000429	250.497955	612.86908	1.1929256	0.021043	DENND1A
0.000024	0.000002	1145.58264	2729.70557	1.1924629	0.012135	BSCL2|HNRNPUL2-BSCL2
0.000007	0.000001	1665.44458	3977.43457	1.1924232	0.01145	HARS
0.000002	0.000001	2725.08228	6284.5376	1.1918682	0.010915	WTAP
0.044983	0.000223	281.941193	692.226562	1.1917249	0.020018	WDR20
0.000003	0.000001	2504.97949	5804.56836	1.1911699	0.011029	MATR3
0.000077	0.000004	876.21875	2114.92432	1.1909692	0.012924	CDC42EP2
0.000001	0.	5262.55322	11858.6826	1.1907798	0.010537	ZZZ3
0.000126	0.000002	1224.1908	2913.27954	1.1900147	0.014166	BRIX1
0.000001	0.000001	3930.40723	9001.81348	1.1899178	0.010737	PRDX4
0.000008	0.000001	1601.50989	3813.93896	1.1885806	0.011715	PDCD6
0.00257	0.000026	453.831024	1112.91675	1.1883802	0.015952	MRPL32
0.010266	0.000068	360.549347	884.405518	1.1882341	0.017631	UBE2L6
0.000001	0.000001	3723.92969	8518.02051	1.1872325	0.010912	MT1X
0.	0.	12576.2549	27490.1914	1.1867359	0.010468	RPL5
0.887535	0.078456	111.09951	252.414108	1.1865369	0.041674	PPAN
0.001538	0.000019	494.707245	1211.39648	1.1864514	0.015565	SMURF2
0.000001	0.	7832.51562	17145.0371	1.1864069	0.010609	SNHG1
0.000002	0.000001	3341.37012	7646.04395	1.1861894	0.011045	RUNX1
0.000012	0.000001	1407.60986	3343.53076	1.1860562	0.012103	FAM168B
0.000089	0.000004	843.727417	2033.65454	1.1851564	0.01337	ALDH1A3
0.000003	0.000001	2424.27515	5609.521	1.18513	0.011386	GGH
0.000001	0.	10403.5254	22787.0664	1.1847903	0.010615	SRSF3
0.000091	0.000004	840.583069	2026.00574	1.184737	0.013408	KLHL20
0.471678	0.006861	162.456833	383.401733	1.1839622	0.029898	SSH1
0.	0.	14136.8887	30760.1016	1.1834558	0.010613	previous version conserved probe
0.688079	0.021127	137.302231	318.385986	1.1831036	0.034651	PRDM6
0.000216	0.000006	704.328979	1715.26855	1.1828358	0.014143	HERPUD2
0.005864	0.000044	400.377472	979.060791	1.1827256	0.017565	ACAT2
0.000344	0.000006	696.992188	1698.05859	1.1823853	0.014786	LSG1
0.078239	0.000418	253.642273	615.737427	1.1805412	0.022313	LINC00472
0.000916	0.000014	540.824036	1318.48132	1.1802523	0.015516	ME1

0.000002	0.000001	2814.17163	6431.7793	1.1801378	0.011518	previous version conserved probe
0.000018	0.000002	1235.71997	2919.97241	1.1797616	0.012716	LPIN1
0.002038	0.000023	471.648865	1147.33691	1.1768598	0.016564	HIST1H2BD
0.000001	0.	5246.83154	11703.792	1.1751883	0.011363	ATP6V1C1
0.874801	0.073906	113.195724	255.282455	1.1731866	0.043095	KLHDC4
0.000151	0.000005	756.734375	1822.35339	1.1731155	0.014522	CCDC85A
0.000017	0.000004	1270.30762	2987.85645	1.1728685	0.015825	NDUFB9
0.186294	0.00133	210.66983	502.915985	1.1724782	0.02579	BEND6
0.077549	0.000433	253.642273	611.912964	1.1715236	0.02311	CHCHD6
0.000769	0.000013	559.690002	1355.76978	1.1711004	0.01604	ZFX
0.000012	0.000002	1380.35901	3244.09497	1.1709515	0.013022	NOL10
0.235768	0.001908	199.140625	473.276459	1.1709315	0.027005	DUSP16
0.290577	0.002709	188.659546	446.505249	1.1696274	0.028238	CAMK1
0.	0.	11491.4629	24844.623	1.1687966	0.011439	JDP2
0.000306	0.000008	659.260315	1593.84216	1.1685895	0.015388	RAD9A
0.000306	0.000008	659.260315	1593.84216	1.1685895	0.015387	CHMP1B
0.000051	0.000003	956.923157	2262.16577	1.1684875	0.014075	MRPL54
0.903946	0.10005	107.955185	240.940735	1.1676463	0.04673	CGGBP1
0.55032	0.011024	153.023849	354.718323	1.1674076	0.033649	NR2C1
0.186798	0.001034	220.102798	524.906616	1.1672004	0.031405	FER
0.000001	0.	9148.93945	19760.0098	1.1661102	0.011651	ASNS
0.39185	0.004949	172.937912	404.436249	1.1623734	0.031104	CCT5
0.007258	0.000042	408.76236	985.753601	1.1618683	0.020262	SNRNP25
0.000081	0.000004	861.545227	2041.30347	1.1615398	0.014873	NUFIP1
0.926102	0.12712	103.762749	229.467377	1.1614704	0.049049	SAMD12
0.000001	0.	6095.79932	13309.1074	1.1611442	0.012088	PSMD12
0.000058	0.000003	928.624207	2188.54517	1.1609402	0.014676	MAP2K4
0.	0.	11510.3281	24715.5488	1.1591667	0.011984	BAX
0.559369	0.011973	151.975739	349.937744	1.1590614	0.035008	UNG
0.00038	0.000009	633.057556	1520.22131	1.1588159	0.016299	ADO
0.521613	0.010014	156.168182	360.454987	1.1585796	0.039022	PHLPP1
0.000001	0.	7510.74609	16150.6787	1.1581541	0.012179	SBDS|SBDSP1
0.000077	0.000002	1415.99463	3297.63745	1.1576213	0.016002	ABL2
0.000003	0.000001	2395.97632	5446.98193	1.1571308	0.013037	PARL
0.463292	0.008963	163.504944	378.621155	1.1566707	0.037779	GON4L
0.000033	0.000003	1058.58972	2466.77417	1.1564374	0.014609	PPHLN1
0.000001	0.	7529.61182	16165.9766	1.1560086	0.012303	ARID5B
0.801263	0.046154	123.676811	278.229187	1.1556252	0.042474	KLHL2
0.678895	0.022696	138.350327	314.561523	1.1546585	0.0386	BRWD3
0.008429	0.000064	373.126648	894.922729	1.1545465	0.020254	PPP2R2D
0.000202	0.000006	713.761963	1703.79529	1.1541719	0.016107	CDC5L
0.000002	0.000001	2607.69409	5876.27686	1.1538937	0.013147	previous version conserved probe
0.005451	0.000047	400.377472	958.982422	1.1519281	0.019828	CWC27
0.000001	0.000001	3909.44507	8723.58496	1.1516973	0.012916	GSTP1
0.269603	0.002541	192.851974	451.285828	1.1513126	0.030475	TBCEL
0.004006	0.000038	421.339661	1008.70032	1.1513086	0.019465	PTPN1
0.000002	0.000001	3273.24316	7309.4917	1.1501303	0.013157	RAE1

0.000019	0.000002	1218.95032	2818.62427	1.1487754	0.014768	METAP2
0.000003	0.000001	2167.48853	4937.37305	1.1482498	0.01373	RASSF1
0.00488	0.000034	434.965057	1036.42761	1.1458432	0.021014	UBQLN1
0.	0.	14129.5518	29916.8086	1.1448944	0.012793	FAM21A|FAM21B|FAM21C
0.51472	0.010164	157.216293	359.498871	1.1446467	0.036121	ATG4D
0.660521	0.023496	140.446548	317.429871	1.1442605	0.042911	WDR20
0.064352	0.000398	262.02713	621.474121	1.1440539	0.025375	TMEM184A
0.000002	0.000001	3231.31885	7185.19727	1.1436987	0.013593	KLF3
0.582916	0.015424	147.78331	335.596039	1.1436951	0.047932	C1orf216
0.000024	0.000002	1165.4967	2683.81226	1.1432004	0.015364	HNRPLL
0.000004	0.000001	2012.36841	4595.08398	1.1430758	0.014179	MED13L
0.037347	0.000236	289.277954	686.489868	1.1409538	0.024422	MARS
0.007546	0.000062	379.415314	901.61554	1.1406362	0.021363	LTBR
0.000421	0.00001	621.528381	1471.45947	1.1383029	0.018033	ATM
0.070733	0.000437	258.882812	610.956909	1.1378778	0.026633	GOSR2
0.000457	0.00001	612.095398	1448.51282	1.1376635	0.018169	MZB1
0.014239	0.000102	341.683411	809.828613	1.1376434	0.022745	PCYOX1
0.847277	0.068255	117.388161	259.106903	1.1376016	0.04776	CYHR1
0.002261	0.000026	470.600739	1112.91675	1.1361982	0.020239	CDC42EP1
0.832481	0.06184	119.484375	263.887482	1.135203	0.047447	ZFP64
0.697202	0.027222	136.25412	305.000397	1.1351099	0.042272	GALNT11
0.000081	0.000004	902.421509	2093.88989	1.1347751	0.016992	UBTD1
0.12595	0.000949	229.535782	536.380005	1.1340977	0.029234	LONRF3
0.000991	0.000016	533.487244	1259.20215	1.133653	0.01934	USP20
0.	0.	29937.125	62638.8555	1.13321	0.013417	IER3
0.793085	0.047968	124.724922	276.316956	1.1331173	0.046031	DDX31
0.651287	0.021508	141.494659	317.429871	1.1328789	0.04131	SPTLC1
0.000005	0.000001	1812.17969	4139.01758	1.1324274	0.015088	ZFAND6|ATP8B5P
0.801264	0.050837	123.676811	273.448608	1.1315254	0.046646	PIP5K1C
0.000002	0.000001	2572.05835	5715.6499	1.1312389	0.014674	RAPGEF2
0.000015	0.000002	1317.47253	3009.84692	1.1307064	0.015888	APOPT1
0.000001	0.	5087.51904	11037.3809	1.1297872	0.014165	CDK7
0.678734	0.025301	138.350327	308.824829	1.1290654	0.042812	ANKRD33B
0.125954	0.000931	229.535782	534.467773	1.1289289	0.029389	COLEC12
0.000876	0.000015	545.016479	1282.14893	1.12891	0.019628	ASB1
0.004592	0.000045	411.906677	971.411865	1.1286302	0.02178	DVL1
0.000001	0.000001	4296.19727	9407.20605	1.1274966	0.014435	NCL
0.000238	0.000007	691.751648	1621.56946	1.1259873	0.018527	BCL7B
0.049101	0.000321	275.652557	646.33313	1.1248413	0.026869	WBP1L
0.000015	0.000002	1297.55847	2949.61182	1.1236867	0.016466	MCTS1
0.000279	0.000008	670.78949	1570.89539	1.1233226	0.018915	FAM21C|FAM21A|FAM21B
0.335017	0.004166	182.370895	416.865723	1.1229639	0.037635	GADD45G
0.03891	0.000259	287.181763	673.104309	1.1225586	0.026497	FOXRED1
0.82492	0.061833	120.532486	263.887482	1.1218334	0.049547	C9orf91
0.000064	0.000004	906.613892	2084.32861	1.1214366	0.017771	HDGFRP2
0.000006	0.000001	1785.97705	4048.18701	1.1207718	0.01598	GLS
0.120556	0.000912	231.631989	536.380005	1.1201472	0.03034	NAA40

0.000319	0.000009	654.019775	1528.82642	1.1200959	0.019336	HIST1H2BL|HIST1H2BK|HIST2H2BF|HIST2H2BA|HIST1H2BM|HIST1H2BD|HIST1H2BF|HIST1H2BB|HIST1H2BE|HIST1H2BI|HIST2H2BC
0.000078	0.000004	884.603638	2034.61072	1.1196688	0.018135	PRPF40A
0.000097	0.000005	830.10199	1915.09644	1.1179842	0.018423	SMIM13
0.001374	0.000014	557.59375	1301.27124	1.1174181	0.022086	MRPS31P5
0.302854	0.003643	186.563324	425.470764	1.1169345	0.035607	DUSP3
0.000041	0.000003	1004.08801	2285.11255	1.1162878	0.017836	KANSL1
0.000003	0.000001	2510.21997	5531.11963	1.1155763	0.015987	COL18A1
0.111737	0.000768	238.96875	552.633911	1.1155008	0.031873	C14orf142
0.005863	0.000055	396.185028	925.518433	1.1148728	0.023596	PAPD7
0.430626	0.007522	167.697372	377.665039	1.114363	0.038865	ATP5G3
0.00024	0.000007	693.8479	1612.96436	1.1140923	0.019613	NUMA1
0.002616	0.000002	1359.39685	3070.08228	1.1138868	0.03189	CNOT7
0.000003	0.000001	2423.22705	5347.5459	1.1136803	0.016042	SC5DL
0.17989	0.001542	213.814148	490.486511	1.1135246	0.033846	KLHDC7A
0.117957	0.000924	232.680099	535.423889	1.110637	0.031551	TJP2
0.000028	0.000001	3437.79614	7466.29443	1.1101287	0.019329	TGIF2-C20orf24|C20orf24
0.000015	0.000002	1319.56873	2971.60254	1.1100421	0.017587	PPAP2A
0.006298	0.000059	390.944489	910.220581	1.1100061	0.024217	KIF2A
0.000618	0.000013	579.604065	1344.29639	1.1085834	0.02114	GORASP1
0.724274	0.035325	133.109787	291.614777	1.1084447	0.047899	C1orf52
0.002069	0.000003	957.971252	2172.29102	1.1062458	0.02926	ZNF639
0.002331	0.00003	461.167786	1067.02332	1.1046776	0.023259	ASMTL
0.669727	0.026733	139.398438	305.956482	1.104537	0.046822	C3orf33
0.03513	0.000251	292.422302	676.928772	1.1039468	0.02849	RASAL2
0.000328	0.000009	650.875427	1503.01135	1.1025041	0.021012	PLGLB2|PLGLB1
0.000001	0.	5098.	10856.6748	1.1021928	0.016179	USP38
0.004457	0.000047	414.002899	958.026306	1.1004192	0.024701	previous version conserved probe
0.025008	0.000192	310.240143	716.129456	1.0991988	0.028478	GTF3C4
0.003946	0.000044	422.387756	975.236328	1.0975909	0.02482	MED29
0.000004	0.000001	2143.38208	4724.15967	1.0972717	0.017543	FAM133B|FAM133CP|FAM133DP
0.000002	0.000001	3143.27759	6772.15576	1.0970437	0.017032	PTX3
0.088882	0.00069	246.305511	563.151184	1.0963806	0.032381	HIST2H4B|HIST2H4A
0.000032	0.000003	1065.92639	2385.50464	1.0962858	0.019391	PPP5C
0.290577	0.003733	188.659546	423.558533	1.0932691	0.038892	OARD1
0.086969	0.000615	251.546051	574.624573	1.0932628	0.033911	RCL1

0.	0.	16241.4902	33053.8203	1.0927733	0.016429	FHL2
0.384377	0.006457	173.986023	387.226196	1.0926346	0.041334	SLC25A42
0.000374	0.00001	635.153809	1456.16174	1.092068	0.022207	RABGAP1
0.000336	0.000009	647.731079	1484.84509	1.0919577	0.022096	GNL3
0.000543	0.000012	593.229431	1359.59424	1.091462	0.022718	ATP11B
0.037523	0.000244	296.614716	680.753235	1.0910406	0.031398	ACOX3
0.001098	0.000026	524.05426	1200.87927	1.0910187	0.025632	TLR4
0.623563	0.02231	144.638977	315.517639	1.0908312	0.047993	MARK3
0.	0.	24225.9824	49486.5508	1.0904602	0.016535	RPL4
0.000188	0.000007	724.243042	1653.12122	1.0895181	0.021701	RABEPK
0.384375	0.006933	173.986023	386.270081	1.089197	0.043299	ATG16L1
0.341712	0.005168	180.274673	401.567902	1.0887171	0.040932	BTRC
0.000001	0.	5348.49805	11243.9014	1.0886063	0.01726	NDUFS8
0.004007	0.000046	421.339661	966.631287	1.0879914	0.025947	PAPD4
0.178513	0.001713	213.814148	481.88147	1.0879082	0.03742	CHAC2
0.020261	0.000084	367.886108	843.292603	1.0873706	0.033359	TTLL1
0.000011	0.000002	1455.82275	3226.88501	1.0863762	0.019411	FKBP3
0.000275	0.00001	672.885681	1535.51917	1.0861584	0.023199	ADAMTS15
0.430625	0.008525	167.697372	370.016144	1.0859118	0.043661	CT45A5|LOC101060211|LOC101060208
0.110495	0.000953	235.824432	533.511658	1.0848605	0.035098	TXNL4B
0.000049	0.000003	968.452332	2162.72998	1.0844159	0.020897	CD99
0.070115	0.000551	257.834717	586.0979	1.0839833	0.033277	PCNX
0.002178	0.000031	466.408325	1063.19885	1.0832573	0.025536	DHX38
0.000068	0.000004	899.277161	2016.44458	1.0832204	0.021331	PTPRM
0.006	0.000062	394.088837	900.659424	1.0823362	0.027336	RBM7
0.000638	0.000014	576.459717	1312.74463	1.0822061	0.023914	ATF1
0.000861	0.000017	547.112671	1245.81665	1.0819952	0.024344	AKAP13
0.	0.	11499.8477	23375.0762	1.0816717	0.017436	PEBP1
0.000007	0.000001	1698.98401	3753.70386	1.0816015	0.019387	MPV17
0.004466	0.00005	414.002899	945.596802	1.0810097	0.026971	MCAT
0.000097	0.000005	829.053894	1864.42236	1.0795932	0.022029	previous version conserved probe
0.001265	0.000022	511.47699	1162.63464	1.0793484	0.025174	CYTH1
0.000009	0.000001	1502.98767	3313.89136	1.0785036	0.01997	WBP5
0.488673	0.011971	160.360611	349.937744	1.0768502	0.046821	ANKRD28
0.000003	0.000001	2290.11719	4953.62695	1.0768211	0.019229	KIFC3
0.000032	0.000003	1070.1189	2363.51392	1.0767435	0.021239	CLEC2B
0.000345	0.00001	644.586792	1461.89844	1.0765285	0.023764	DMRTA1
0.000018	0.000002	1245.15295	2737.35449	1.0760137	0.020778	CRHBP
0.000024	0.000002	1155.01562	2541.35107	1.0750345	0.02113	NFATC3
0.000024	0.000002	1210.56543	2658.95312	1.0748814	0.021239	ELAVL1
0.000001	0.000001	4234.35889	8946.35938	1.0739424	0.018694	RPS6
0.000001	0.	5914.47656	12213.4014	1.0739384	0.018443	CDKN1A
0.000003	0.000001	2520.70117	5395.35156	1.0723545	0.019487	PRKRIP1|LOC100630923
0.00011	0.000005	807.043579	1808.96777	1.072022	0.022956	KCTD5
0.000013	0.000002	1352.06006	2964.90967	1.0716447	0.020943	KLC1
0.001124	0.000021	521.958069	1179.84473	1.0713365	0.025939	SIL1
0.000001	0.000001	3715.54492	7849.69629	1.0708596	0.019122	CCT2

0.505955	0.013459	158.264389	343.244934	1.0701409	0.04876	ACN9
0.296612	0.004291	187.611435	413.997375	1.0698881	0.043017	BLMH
0.000001	0.000001	4614.82227	9673.96191	1.0697848	0.018998	SERPINH1
0.000002	0.000001	3303.63818	6975.80811	1.0683196	0.019482	NTN4
0.020205	0.000173	321.769348	727.602783	1.0682771	0.031802	C6orf99
0.430618	0.009227	167.697372	365.235565	1.0678294	0.046973	METTL2A|METTL2B
0.115405	0.001068	233.72821	522.038269	1.0670977	0.037856	previous version conserved probe
0.240856	0.003049	198.092514	437.900238	1.0665916	0.042064	H2AFJ
0.212484	0.002414	205.429276	455.110291	1.0665744	0.042278	AHCYL2
0.00001	0.000002	1490.4104	3256.52441	1.0655693	0.021258	POLR2C
0.000048	0.000003	971.59668	2141.69556	1.0652447	0.022827	HMGN3
0.016725	0.000149	332.250427	749.593445	1.0646154	0.031847	FOXO4
0.000001	0.	5653.49756	11642.6006	1.0643885	0.019331	GBA
0.006096	0.000067	393.04071	887.273865	1.0640623	0.0297	VASP
0.001206	0.000022	515.669434	1158.81018	1.0628642	0.027069	GUSB
0.066681	0.000343	284.037415	638.684204	1.0628181	0.042181	SYNCRIP
0.086975	0.000848	247.353622	552.633911	1.0625797	0.038767	previous version conserved probe
0.001249	0.000022	513.573181	1153.07361	1.0615759	0.027304	CSRP2
0.000165	0.000006	755.686279	1688.49744	1.0608092	0.024729	WDR12
0.000001	0.000001	3511.16382	7370.68311	1.0606557	0.020138	PCNA
0.256727	0.003486	194.948196	428.339111	1.0591947	0.043869	LIN52
0.000803	0.000017	553.401306	1239.12378	1.0578029	0.027085	SLC27A1
0.000002	0.000001	3219.78955	6751.12109	1.0576368	0.02054	KRT10
0.000644	0.000015	575.411621	1287.88562	1.0572932	0.026828	AQP12B|AQP12A
0.001037	0.00002	529.294861	1183.66919	1.0559233	0.027705	ADSSL1
0.01182	0.000116	352.16449	788.794067	1.0533622	0.032615	LIF
0.048883	0.000423	275.652557	614.781311	1.0524073	0.036812	S100A16
0.000001	0.	5240.54297	10766.8008	1.0516994	0.020606	MARS
0.290578	0.004478	188.659546	411.129028	1.0516716	0.046197	NHLH1
0.036636	0.000316	290.32608	648.2453	1.0515875	0.035961	PRMT10
0.000004	0.000001	2109.84253	4515.72656	1.0511845	0.021889	NOLC1
0.407078	0.008797	170.841705	368.103912	1.0502006	0.049782	ZBTB49
0.000048	0.000004	968.452332	2113.01196	1.0495881	0.024563	SSR2
0.	0.	20235.832	40222.7617	1.0482439	0.020315	UBE2J2
0.004191	0.000053	418.195312	934.123413	1.0479199	0.03116	ATP5SL
0.000001	0.000001	3890.5791	8087.76855	1.0478996	0.021277	TLE4
0.000004	0.000001	2301.64648	4876.18164	1.046114	0.022493	TOB2
0.000004	0.000001	2169.58472	4617.07471	1.0458678	0.022406	ADI1
0.001235	0.000022	521.958069	1158.81018	1.0454326	0.029521	FAM179B
0.007674	0.000084	378.367188	843.292603	1.0453769	0.032784	TCEAL8
0.000009	0.000001	1556.44128	3354.0481	1.0452902	0.023383	RILPL2
0.000298	0.000007	727.387329	1609.13989	1.0446276	0.027931	TOM1L1
0.000002	0.000001	2637.04126	5514.86572	1.0426506	0.022378	SOBP
0.000001	0.000001	3505.9231	7266.4668	1.0419697	0.022021	ITGA3
0.000009	0.000002	1532.33472	3293.81299	1.0418242	0.023719	XPA
0.000118	0.000006	795.514404	1748.73254	1.0414395	0.026551	KDM5A

0.000021	0.000002	1186.45886	2548.04395	1.0401506	0.02479	ADAMTS5
0.000069	0.000004	891.94043	1939.95544	1.0367715	0.02649	STX5
0.000005	0.000001	1891.83594	4033.84521	1.0357583	0.023804	FBXO30
0.033066	0.000304	295.56662	653.025879	1.0354789	0.038213	MCPH1
0.000088	0.000005	845.823608	1844.34399	1.0353606	0.026952	SMAD5
0.000342	0.000011	645.634888	1421.74158	1.0340843	0.028885	UBALD1
0.000001	0.	8030.60791	15833.249	1.0332723	0.022202	ATP6V0B
0.000506	0.000005	835.342529	1820.44116	1.0332713	0.032111	PDHB
0.000001	0.	6544.39014	13044.2637	1.0330835	0.022376	ARL6IP1
0.226025	0.003172	201.236847	435.031891	1.0329134	0.047859	MVB12B
0.003405	0.000048	432.868835	956.114075	1.0329093	0.032908	RNF25
0.008783	0.000097	369.98233	817.477539	1.0326671	0.035001	HRAS
0.000011	0.000002	1424.37952	3040.44263	1.0324598	0.025015	CLCN6
0.000001	0.	5155.646	10466.5801	1.032277	0.023278	SUV420H1
0.000044	0.000004	986.270142	2124.48535	1.0321904	0.026552	ASPH
0.001885	0.000032	477.9375	1051.72546	1.0321307	0.031916	previous version conserved probe
0.000004	0.000001	2141.28589	4519.55127	1.031929	0.023954	DCTN3
0.261942	0.004114	193.900085	416.865723	1.0288945	0.049801	TMEM25
0.000001	0.000001	4647.31348	9469.35352	1.0288094	0.023114	PTOV1
0.000004	0.000001	2117.17944	4462.18408	1.0283986	0.024387	BAG1
0.00001	0.000002	1463.15955	3107.37061	1.0249058	0.025828	EIF6
0.000001	0.000001	3719.7373	7612.58008	1.024314	0.023878	MPDU1
0.000005	0.000001	1870.87378	3957.35596	1.0237268	0.025234	RNF19A
0.015174	0.000109	363.693665	798.355225	1.0231141	0.040477	CAB39L
0.035854	0.000346	291.374176	637.728088	1.022527	0.040694	ALS2CR12
0.090831	0.000951	245.257401	532.555542	1.0220777	0.04457	previous version conserved probe
0.011016	0.000123	356.356903	781.145203	1.0211881	0.037502	KIAA1143
0.000001	0.000001	3492.29785	7129.74268	1.0197998	0.024486	WAC
0.024048	0.00024	312.336365	682.665405	1.0180044	0.040159	CLCN5
0.078063	0.000807	252.594162	547.853333	1.0178329	0.044651	previous version conserved probe
0.000009	0.000001	1596.26941	3375.08252	1.0177477	0.026585	HNMT
0.000009	0.000002	1530.23853	3234.53394	1.0177195	0.026565	SLC19A2
0.000025	0.000003	1138.24597	2410.36353	1.016928	0.027831	ZNF721
0.000027	0.000003	1112.04321	2355.86499	1.016343	0.028004	SPCS1
0.000017	0.000002	1258.77844	2654.17261	1.015737	0.027555	FOXJ3
0.000003	0.000001	2459.91089	5079.83398	1.0154579	0.025604	HYI
0.000003	0.000001	2513.36426	5181.18213	1.0153823	0.025571	TMEM132A
0.000007	0.000001	1676.97375	3538.57812	1.0149341	0.026614	OTUD3
0.000692	0.000017	568.074829	1234.34326	1.0146021	0.032722	CTIF
0.000002	0.000001	4600.14844	9282.91113	1.0137588	0.025808	RSL1D1
0.000084	0.000003	1148.72705	2426.61743	1.0137439	0.030972	CCDC53
0.019655	0.00019	328.057983	715.17334	1.0137336	0.040915	ING4
0.000005	0.000001	1929.56787	4048.18701	1.0134476	0.026404	C2orf27B|C2orf27A
0.001049	0.000023	528.246704	1146.38074	1.0126808	0.033737	NOP16

0.000001	0.000001	4468.08691	9024.76074	1.0126296	0.025278	PRDX2
0.000457	0.000013	612.095398	1328.04248	1.0126282	0.032328	DCAF10
0.117952	0.001375	232.680099	500.047638	1.011707	0.047936	CCL20
0.000003	0.000001	2225.13452	4617.07471	1.0107804	0.026393	PDPK1
0.018725	0.000198	325.961761	708.48053	1.0093415	0.040908	RGS7
0.001539	0.00003	494.707245	1070.84778	1.0088687	0.035046	MRPL44
0.000002	0.000001	2565.76978	5257.67139	1.0088184	0.026339	FRMD6
0.000019	0.000002	1216.854	2556.64893	1.0086367	0.028621	SUGT1
0.038127	0.000388	288.229858	624.342468	1.0080453	0.043534	REM2
0.000001	0.	8067.2915	15633.4209	1.0077475	0.025091	PPA1
0.000001	0.000001	3422.07446	6908.88037	1.0031611	0.026538	DPM1
0.000008	0.000001	1599.4137	3345.44312	1.0022347	0.028419	CAPRIN1
0.000003	0.000001	2500.78711	5112.3418	1.0021951	0.027291	TXNDC5|BLOC1S5-TXNDC5
0.005691	0.00006	420.291534	909.264465	1.0006403	0.04055	CDC6
0.000003	0.000001	2547.9519	5193.61133	1.0000229	0.027717	SMCR8

0.010085	0.983731	361.597473	86.050263	-2.08492	0.0017773	AMT
0.000001	0.000005	4462.84619	1784.10876	-1.37924	0.0047276	FUCA1
0.000021	0.005479	1187.50696	397.743439	-1.681243	0.001974	TRIM21
0.001836	0.233571	480.033722	201.740067	-1.328611	0.015427	GDF5
0.000001	0.000001	9336.55078	3902.85767	-1.262823	0.0075839	ST6GAL1
0.000002	0.000012	2805.78662	1380.62866	-1.084008	0.0199636	HLTF
0.000042	0.000961	1000.94366	531.599426	-1.019899	0.0339907	SLC39A11
0.	0.000001	15612.625	6998.75488	-1.106995	0.0156208	NCSTN
0.000002	0.000008	3128.604	1562.29041	-1.063823	0.0216306	UBE2T
0.000309	0.010671	658.212158	356.630554	-1.001406	0.0432144	ALDH5A1
0.	0.000002	12235.6191	3121.7124	-1.962398	0.0003467	BLCAP
0.000013	0.000193	1359.39685	712.304993	-1.034258	0.0290927	IDH2
0.000001	0.000004	4188.2417	2049.90845	-1.083699	0.0190163	NCAPD2
0.000001	0.000004	5260.45703	2067.11865	-1.385492	0.0044939	NAV1
0.000001	0.000005	5901.89941	1818.52893	-1.736023	0.0009367	SEMA4C
0.000003	0.000023	2379.20654	1150.2052	-1.114174	0.017943	PHF13
0.000131	0.005244	778.74469	400.611786	-1.075158	0.029781	C19orf25
0.00002	0.00076	1214.75781	553.590027	-1.238927	0.0123784	HDHD3
0.000054	0.040348	941.201477	284.921997	-1.814106	0.0013971	KALRN
0.000001	0.000003	4883.1377	2251.64868	-1.154808	0.0133514	ANGPTL2
0.00002	0.000378	1208.46924	627.210815	-1.050719	0.028021	FAM160A2
0.000007	0.000174	1665.44458	727.602783	-1.29603	0.0085887	EXTL3
0.001753	0.121704	484.226166	233.291824	-1.144827	0.0301127	PLEKHA4
0.000002	0.000016	2572.05835	1263.98279	-1.086515	0.0200199	SLC25A13
0.000001	0.000004	3825.59619	1942.82373	-1.03305	0.0242614	ZDHHC7
0.000004	0.000136	2180.06592	792.61853	-1.542525	0.0027409	FST
0.000022	0.014898	1168.64111	337.50827	-1.886441	0.0009182	TP53INP2
0.000005	0.000237	1902.31702	684.577637	-1.571184	0.0024786	LAMA5
0.001866	0.255224	494.707245	196.003387	-1.41088	0.012154	MMACHC
0.000003	0.000028	2266.01074	1090.92615	-1.122715	0.0173616	BOLA1
0.00038	0.020388	633.057556	320.298218	-1.094777	0.0303635	SMOC1
0.00275	0.165163	448.590485	217.037888	-1.132451	0.0323625	TYSND1
0.001825	0.048023	625.720825	276.316956	-1.283397	0.0306504	C2CD2
0.000072	0.006869	882.507446	383.401733	-1.311219	0.0105195	CUTC
0.000004	0.00005	2139.1897	945.596802	-1.253963	0.0096694	CCDC14
0.000002	0.000044	3014.36035	974.280212	-1.693816	0.0012808	NOD1

0.008834	0.990062	372.078552	81.269692	-2.210096	0.0013019	LRIG3
0.00125	0.405574	512.525085	172.100525	-1.637445	0.0045594	IFIT1
0.001049	0.10183	528.246704	239.984634	-1.23175	0.0198584	ZNF691
0.000046	0.001165	975.789062	514.389343	-1.03192	0.03259	STAT1
0.000303	0.078456	660.308411	252.414108	-1.484442	0.0063594	WDR89
0.000025	0.004477	1131.95728	411.129028	-1.56597	0.0032145	SLC7A8
0.000291	0.034666	665.54895	292.570892	-1.292418	0.0134103	RGS7BP
0.000001	0.00001	3383.29443	1457.1178	-1.277594	0.0078955	CHFR
0.00004	0.000959	1011.4248	538.292236	-1.016366	0.0346483	CCNB1
0.000036	0.00156	1036.57935	489.530396	-1.188164	0.016369	MIR17HG
0.000014	0.000284	1343.67517	661.63092	-1.126689	0.0194545	EML2
0.000008	0.006663	1610.94287	386.270081	-2.162741	0.0002991	ARRDC3
0.000001	0.000003	5313.91064	2374.03125	-1.192126	0.0111013	RGS5
0.000004	0.000799	2160.15186	548.809448	-2.068403	0.0003405	MTFP1
0.000001	0.000004	8656.3291	2032.69849	-2.108815	0.0002079	SLC29A1
0.000031	0.008938	1080.59998	367.147797	-1.656183	0.002307	SEMA4F
0.011608	0.63731	353.212585	144.373215	-1.330477	0.0194061	C5orf30
0.000003	0.000026	2239.80811	1108.13623	-1.084034	0.0207619	FAM174B
0.000132	0.021902	776.648438	316.473755	-1.399203	0.0079219	TMEM155
0.000002	0.000012	2616.0791	1363.4187	-1.001021	0.0295406	ITPRIP
0.000008	0.000242	1598.3656	681.709351	-1.333581	0.0073724	ZNF449
0.000022	0.001244	1181.21838	508.652679	-1.320984	0.0088295	ENGASE
0.00001	0.000178	1467.35205	784.01355	-1.002149	0.033947	PDIK1L
0.000182	0.073903	728.435486	255.282455	-1.606625	0.0036955	RTN4R
0.000019	0.003632	1225.23889	425.470764	-1.632312	0.0023539	TMEM51
0.000144	0.004605	764.071167	409.216827	-1.018992	0.0377437	SESTD1
0.000645	0.05497	685.463013	269.624176	-1.447241	0.0121085	XYLT2
0.000001	0.000002	6589.4585	2662.77759	-1.31691	0.006038	ZNF395
0.000008	0.006654	1589.98071	385.313965	-2.147256	0.0003046	FIBIN
0.000001	0.000007	3785.76807	1643.56006	-1.267096	0.0081411	AP1S2
0.000006	0.000061	1709.46509	905.440002	-1.007237	0.0310054	PRRG1
0.008347	0.967731	373.126648	93.699181	-2.008982	0.0020929	FZD3
0.000004	0.000027	2150.71875	1121.52173	-1.010413	0.0294136	MSC
0.000001	0.000001	7462.5332	3603.59375	-1.058999	0.0203147	LDOC1
0.004067	0.209815	420.291534	205.564514	-1.112107	0.0364729	TMPRSS5
0.	0.005985	30523.0176	13487.9014	-1.119825	0.0455705	C1orf122
0.000257	0.052705	681.270569	272.492493	-1.423943	0.0079018	SEL1L3
0.000376	0.013234	637.25	344.20105	-1.004189	0.0437112	LINC00174
0.000033	0.000768	1060.68591	552.633911	-1.04539	0.0299042	VPS13A
0.000017	0.000824	1259.82654	545.941101	-1.311719	0.0088723	RIPK4
0.000052	0.068257	959.019348	259.106903	-1.971307	0.000806	ABCC10
0.000769	0.03402	557.59375	293.527008	-1.033605	0.0412807	DHX58
0.	0.000001	27673.2109	9992.34766	-1.42327	0.0033913	LBH
0.000003	0.00018	2384.44702	721.866089	-1.805867	0.0008728	SLC11A2
0.000059	0.001979	950.63446	470.408112	-1.124738	0.0231534	RTKN
0.000009	0.000045	1919.08679	970.45575	-1.064703	0.0275045	GPC4
0.000012	0.000469	1409.70605	603.307983	-1.329586	0.0078541	SPTLC2
0.003167	0.894692	438.109375	91.786949	-2.275976	0.0017892	ZC3H10
0.001657	0.07539	488.41861	254.32634	-1.04061	0.0432312	previous version conserved probe

0.000114	0.382172	817.524719	181.661667	-2.227642	0.0006692	previous version conserved probe
0.000016	0.000571	1290.22168	582.273438	-1.252931	0.0113444	RBBP9
0.000947	0.057166	537.679688	267.711945	-1.109313	0.0314357	EFCAB4A
0.000002	0.000008	3476.57617	1605.31555	-1.177531	0.0132083	TBC1D14
0.000048	0.002476	970.548523	453.198059	-1.207686	0.0154933	KIF20A
0.000001	0.000001	9902.53027	4563.53223	-1.116393	0.0152162	ST3GAL4
0.000116	0.004877	798.658752	405.392365	-1.093991	0.0272104	SLC16A2
0.000002	0.000011	2805.78662	1425.56604	-1.037886	0.0247969	NAGA
0.000008	0.00009	1598.3656	830.863098	-1.039501	0.0272724	QDPR
0.000001	0.000002	5823.29102	2925.70898	-1.011191	0.0266856	IL4R
0.002039	0.143471	471.648865	223.730682	-1.163508	0.0278407	GPM6A
0.008356	0.338935	373.126648	181.661667	-1.101553	0.0412774	SNN
0.000001	0.000002	7342.00049	3202.98218	-1.205118	0.0101611	PDIA5
0.000001	0.000001	8643.75098	4039.58179	-1.103797	0.0162703	BTD
0.000003	0.000019	2484.01733	1213.30872	-1.097095	0.019209	RASSF2
0.000002	0.000012	2698.87964	1373.93591	-1.03471	0.0252005	GPD1L
0.040545	0.799975	285.085541	125.250938	-1.202615	0.0365313	KDELC2
0.000251	0.245499	684.414917	197.915604	-1.863239	0.0015525	MOGS
0.002237	0.214013	464.312103	204.608414	-1.261652	0.0195272	NA
0.014967	0.520148	338.539062	157.75882	-1.146944	0.0379508	CLGN
0.000001	0.000006	4397.86377	1831.91455	-1.319416	0.0062855	PML
0.000017	0.00313	1271.35571	435.988007	-1.650376	0.0021473	NA
0.000069	0.004413	891.94043	412.085144	-1.225774	0.014944	GRAMD4
0.000008	0.000337	1581.59583	640.596436	-1.409088	0.0053381	PKLR
0.000387	0.030418	642.49054	299.263702	-1.210501	0.0198023	DUS4L
0.007816	0.358075	377.319092	178.793335	-1.139754	0.0357811	ECHDC3
0.000025	0.000715	1131.95728	580.361206	-1.068536	0.0270696	TRIM66
0.000003	0.000037	2289.06909	1013.4809	-1.244903	0.0099158	TNFAIP2
0.000071	0.001315	957.971252	503.872101	-1.036034	0.0353802	GEMIN2
0.000199	0.209828	715.858154	205.564514	-1.874861	0.0014281	JRKL
0.0001	0.00474	823.813354	407.304596	-1.130834	0.0230465	previous version conserved probe
0.000319	0.053906	654.019775	270.580292	-1.376181	0.0097477	TRMT1L
0.000003	0.000016	4434.54736	1267.80725	-1.86584	0.0009476	DDIT4L
0.000005	0.000086	1985.11768	840.424255	-1.325469	0.0073156	B4GALT5
0.000009	0.000153	1556.44128	745.768982	-1.161675	0.0160918	GLG1
0.002255	0.182571	476.889404	212.257324	-1.250512	0.0219605	ACBD4
0.000002	0.000014	2751.28491	1322.30579	-1.117734	0.017141	MEGF8
0.00001	0.002295	1487.26611	458.934753	-1.802861	0.0010845	PANK1
0.000003	0.000033	2425.32324	1044.07654	-1.280944	0.0083051	previous version conserved probe
0.000006	0.000025	2668.48438	1119.6095	-1.313061	0.0097511	SLC17A5
0.000001	0.000001	7445.76318	3670.52197	-1.029372	0.0233794	MXI1
0.000001	0.000003	4938.6875	2292.76147	-1.14331	0.0140803	FLJ37453
0.000001	0.000006	4017.40015	1743.95203	-1.265659	0.0081341	HPS6
0.000013	0.000481	1376.1665	600.439636	-1.301678	0.0089568	ACCS
0.001025	0.345237	530.342957	180.705551	-1.620684	0.0046732	BIRC3

0.00013	0.182575	789.225769	212.257324	-1.968092	0.0009936	SPRYD4
0.00742	0.990634	380.463409	79.357468	-2.277761	0.0010229	DUSP18
0.000678	0.029853	570.171082	300.219818	-1.034334	0.0406897	SCN2B
0.000011	0.000157	1414.94653	744.812866	-1.025819	0.0299284	C1QL1
0.00098	0.064322	534.5354	261.97525	-1.130335	0.0290921	VANGL2
0.002182	0.671232	471.648865	140.548767	-1.792164	0.0031675	FRAT2
0.000016	0.011208	1318.52063	353.762207	-1.995455	0.0006026	LRFN3
0.067161	0.879594	259.930939	113.777573	-1.194153	0.0410766	SH3RF2
0.000582	0.186253	585.8927	211.301208	-1.552647	0.0054396	GTF2E1
0.00141	0.085455	502.044006	248.589661	-1.110998	0.0326591	RSPH6A
0.000003	0.000141	2385.49512	757.24231	-1.735367	0.0011579	ITGA5
0.000013	0.00069	1384.55139	563.151184	-1.40325	0.0059489	FOXS1
0.00016	0.017407	749.397644	328.903229	-1.295663	0.0123734	TARBP1
0.000139	0.058298	769.311707	266.755829	-1.623571	0.0033197	TRAM1L1
0.000003	0.000017	2407.50537	1237.21155	-1.025582	0.0267562	TRAK2
0.000001	0.000002	6234.1499	2764.12573	-1.1868	0.011217	FAM57A
0.000001	0.000003	5170.31982	2209.57959	-1.261854	0.0080143	ZNF217
0.000001	0.000046	5825.3877	962.806824	-2.642763	4.218E-05	TRIB2
0.000056	0.00667	933.864746	385.313965	-1.38344	0.007567	ZBTB14
0.000248	0.021132	686.511108	318.385986	-1.218226	0.0177299	C5orf42
0.047653	0.784952	284.037415	127.16317	-1.176585	0.0471237	FRMPD3
0.377044	0.998659	175.034134	65.97187	-1.367433	0.0378045	STOX2
0.000745	0.250395	560.738098	196.959488	-1.584484	0.0050437	CTNND1|TMX 2-CTNND1
0.000003	0.000031	2239.80811	1061.28662	-1.146238	0.0157325	TEAD2
0.003044	0.594907	441.253723	149.153793	-1.615872	0.0057812	TCTN2
0.000041	0.000964	1008.28046	532.555542	-1.027516	0.0328833	COL4A5
0.006477	0.000072	4345.45801	1034.51538	-2.131834	0.0483425	COL1A1
0.00002	0.000941	1202.18054	533.511658	-1.277352	0.0104921	GNB4
0.000012	0.001216	1397.12878	510.564911	-1.558199	0.0029776	RPUSD2
0.167175	0.987848	215.91037	83.181923	-1.355091	0.0304168	ZNF572
0.00001	0.000297	1461.06335	655.894226	-1.260355	0.0104983	PARP1
0.001596	0.153075	491.562927	222.774582	-1.228766	0.0219255	MCM3
0.000018	0.000485	1245.15295	599.483521	-1.159381	0.0172849	RAB20
0.000002	0.000025	2542.71143	1119.6095	-1.24524	0.0096753	LPCAT1
0.000296	0.161878	663.452759	217.994003	-1.689192	0.0029499	LONRF1
0.000005	0.000049	1880.30676	949.421265	-1.06894	0.0231871	KNSTRN
0.001111	0.059057	523.006165	268.66806	-1.064564	0.0387035	IFIT3
0.000107	0.507677	811.236023	156.802704	-2.415883	0.0064583	previous version conserved probe
0.000142	0.008837	766.167358	368.103912	-1.170052	0.0203519	HOXB8
0.000011	0.001103	1455.82275	672.148193	-1.219558	0.0148775	FRS3
0.000005	0.000069	1951.57812	879.624939	-1.234062	0.0108274	MAP1A
0.000002	0.000051	3261.71387	940.816223	-1.86005	0.0006391	PFKFB4
0.000052	0.042724	952.730713	282.05365	-1.845209	0.0012372	SOX13
0.035852	0.821778	291.374176	122.382599	-1.266614	0.0290224	IGSF9B
0.000001	0.000005	3715.54492	1839.56348	-1.072537	0.0202505	GAL3ST3
0.	0.000001	10592.1855	4307.29395	-1.298761	0.0063449	MARVELD1
0.000007	0.000086	1672.78125	839.46814	-1.089572	0.0215728	RAB31

0.267487	0.999285	192.851974	62.147415	-1.602353	0.0176471	previous version conserved probe
0.000215	0.017104	833.246338	329.859344	-1.440015	0.009663	SIX3
0.	0.000001	19184.5801	8020.84082	-1.205455	0.0096642	RAB1A
0.000001	0.000005	5305.52539	1949.5166	-1.484972	0.0028604	CAPRIN2
0.000034	0.004949	1054.39722	404.436249	-1.486199	0.0046311	SLCO4A1
0.000139	0.005997	769.311707	392.006775	-1.0883	0.0283597	PDCD11
0.009407	0.79252	365.789886	126.207054	-1.564942	0.0084681	CALHM2
0.006031	0.260189	397.233154	195.047272	-1.099102	0.041023	LRFN2
0.000001	0.000001	8902.63379	3624.62842	-1.30141	0.0063378	SMARCD2
0.000033	0.000657	1056.49341	567.931763	-1.000146	0.0363179	AURKA
0.000003	0.000025	2214.65332	1124.39014	-1.047391	0.0245833	SLC2A4RG
0.000022	0.000481	1182.26648	600.439636	-1.082166	0.0246173	BIRC7
0.000001	0.000017	3837.12549	1250.59717	-1.680088	0.0012802	PCYOX1L
0.000013	0.001694	1363.58923	482.837585	-1.60389	0.0024962	PCED1B
0.000926	0.215419	539.77594	204.608414	-1.478226	0.0077651	ABHD10
0.000038	0.005813	1026.09827	393.918976	-1.484506	0.0047353	LMBR1L
0.000003	0.000016	2852.95166	1269.71948	-1.228754	0.0116692	ADCK3
0.000001	0.000001	7676.34717	3372.21436	-1.194458	0.0106423	PYCR2
0.000001	0.000023	3374.90967	1144.46851	-1.621781	0.0016888	CPT2
0.000007	0.000159	1639.24182	740.032288	-1.248071	0.0106827	NRBP2
0.000333	0.031619	677.078125	297.351471	-1.29391	0.0146386	TUBG2
0.004921	0.603352	407.714233	148.197678	-1.509571	0.009105	MED7
0.000428	0.015417	699.08844	335.596039	-1.170425	0.027348	SEC62
0.000959	0.011584	748.349548	351.849976	-1.199927	0.0375558	SLC25A37
0.245949	0.997951	197.044418	68.84021	-1.487798	0.0233542	PRDM12
0.000074	0.024399	879.363098	310.737061	-1.598423	0.0033125	FAM76B
0.001182	0.297573	517.765625	188.354462	-1.530167	0.0066384	TMEM254
0.000509	0.047043	600.566223	277.273071	-1.219586	0.0191246	BIVM
0.000001	0.000014	3526.88525	1313.70068	-1.487027	0.0030183	TM2D3
0.414823	0.99892	169.793594	63.103527	-1.384916	0.0392283	MESDC1
0.000033	0.001034	1059.63782	524.906616	-1.11846	0.0218748	LYSMD2
0.000041	0.002104	1005.13611	465.627533	-1.217519	0.0146383	IGIP
0.000115	0.00548	799.706848	397.743439	-1.122312	0.0241905	SLC25A43
0.001515	0.201678	518.813721	207.476746	-1.402459	0.0125069	B3GNT7
0.000002	0.000007	3341.37012	1664.5946	-1.067832	0.0209764	ZSWIM4
0.00001	0.000184	1479.92932	718.997742	-1.143151	0.0175693	SEH1L
0.001884	0.076916	477.9375	253.370224	-1.014501	0.0482818	RAC2
0.00001	0.000175	1456.87097	725.690552	-1.106682	0.02074	ZNF688
0.00011	0.002675	858.40094	447.461365	-1.054328	0.0338791	SZT2
0.000014	0.000329	1465.25574	643.464783	-1.292186	0.0102652	GEMIN4
0.000045	0.013459	985.222046	343.244934	-1.619105	0.0028311	FZD8
0.000003	0.000085	2180.06592	877.712708	-1.390957	0.0052778	PPP1R3C
0.	0.	68665.7891	29045.7891	-1.167891	0.0113402	CCL2
0.000103	0.00323	828.005798	434.075775	-1.047844	0.0330202	previous version conserved probe
0.001767	0.201661	483.17804	207.476746	-1.300124	0.0164002	ZNF343

0.000211	0.021131	710.617615	318.385986	-1.266512	0.014399	previous version conserved probe
0.000024	0.001068	1152.91943	522.038269	-1.24834	0.0120974	IRF5
0.000003	0.000027	2325.75293	1137.77576	-1.098283	0.0194909	RAX2
0.000006	0.001511	1731.47534	497.179321	-1.904393	0.0006875	CBX7
0.006241	0.729527	401.425598	133.855972	-1.624207	0.0073053	BPIFA3
0.00002	0.000449	1208.46924	609.044678	-1.093257	0.023316	FRMD8
0.001131	0.038103	536.631592	287.790344	-1.005944	0.0497751	previous version conserved probe
0.000024	0.000627	1148.72705	572.712341	-1.108994	0.022136	DUT
0.000002	0.000019	3262.76196	1273.54395	-1.418928	0.0045324	DLG1-AS1
0.006505	0.488121	388.848297	161.583282	-1.321612	0.0182777	CEP57L1
0.067194	0.926379	259.930939	105.172546	-1.301416	0.0292009	CCDC89
0.000004	0.000107	2097.26538	802.179688	-1.470964	0.0037299	PARS2
0.000257	0.01033	681.270569	358.542755	-1.042335	0.0361165	OAF
0.000097	0.334203	830.10199	200.783951	-2.113807	0.0013563	previous version conserved probe
0.000014	0.000072	2009.22412	871.976013	-1.287444	0.0125697	PPP1R35
0.004464	0.934859	510.428864	103.260315	-2.325768	0.0026482	PKD1L2
0.000001	0.000001	9553.50977	4586.479	-1.057843	0.0201527	KCNS3
0.000006	0.000061	1759.77429	906.396118	-1.045923	0.0259217	RIMKLA
0.012517	0.769518	349.020142	129.075394	-1.464347	0.0125308	IRAK2
0.000003	0.00002	2301.64648	1183.66919	-1.02688	0.0268015	MTMR11
0.000001	0.000007	3539.46265	1612.0083	-1.197519	0.011371	EIF1AD
0.000053	0.004351	948.538269	413.04126	-1.308628	0.0102364	HDHD1
0.000023	0.000696	1162.35242	562.195068	-1.152863	0.0182238	HSPA2
0.000099	0.001501	923.383667	494.310974	-1.012312	0.0414346	NUDT8
0.000021	0.000615	1193.79565	584.185669	-1.135911	0.019617	MDC1
0.00001	0.001969	1472.59253	471.364227	-1.749854	0.0013416	SLC26A6
0.000002	0.000013	2671.62866	1343.34021	-1.052433	0.0232682	TMEM8B
0.000056	0.250317	942.249634	196.959488	-2.317081	0.0002768	previous version conserved probe
0.000001	0.000006	3578.24268	1693.27795	-1.142059	0.0147098	LRFN4
0.000019	0.001587	1216.854	627.210815	-1.060713	0.0315926	CNDP2
0.001004	0.175398	564.930542	214.169556	-1.482037	0.009102	MAPK7
0.000444	0.093974	615.239746	243.809082	-1.429629	0.0082954	PIGN
0.000009	0.000157	1505.08386	741.944519	-1.120819	0.0192996	AARS2
0.	0.000001	20953.7871	9193.99316	-1.135518	0.0135019	LRP1
0.000002	0.00004	2801.59424	997.226929	-1.55314	0.0023776	METTL3
0.000002	0.000593	3436.74805	580.361206	-2.648272	6.782E-05	previous version conserved probe
0.085112	0.942711	248.401733	101.348091	-1.285235	0.0321777	DCAKD
0.000001	0.000005	4503.72266	1906.49146	-1.292459	0.0071906	ZMYND8
0.000016	0.000408	1284.98108	618.605774	-1.159446	0.0171137	SMC1A

0.000113	0.0038	802.851196	422.602417	-1.04345	0.0334682	RRM2
0.000001	0.000002	5809.66602	2825.31714	-1.058109	0.0209091	MEGF9
0.000003	0.000026	2190.54688	1108.13623	-1.05317	0.0240077	MNT
0.01101	0.737648	356.356903	132.899857	-1.455893	0.0125525	LAMA1
0.182341	0.974627	211.717926	90.830833	-1.198088	0.0493934	ZNF320
0.000027	0.000609	1112.04321	575.580688	-1.054852	0.0282647	CA12
0.000018	0.000753	1233.62378	554.546143	-1.258705	0.0112593	RAB22A
0.005041	0.255203	405.618011	196.003387	-1.123722	0.0358577	TMEM101
0.000004	0.000154	2076.30298	744.812866	-1.567305	0.0024467	ADAM19
0.000001	0.000022	4636.83252	1157.85413	-2.057985	0.0002771	NUP210
0.000004	0.000007	3511.16382	1646.42834	-1.155463	0.0181046	TNFRSF25
0.001744	0.475378	484.226166	163.495499	-1.625157	0.0059648	ASB13
0.001884	0.080038	477.9375	251.457993	-1.024735	0.0464646	TUBB2B
0.000062	0.012379	914.998779	348.025513	-1.496416	0.0048309	LOC283050
0.000123	0.075524	788.177673	254.32634	-1.721977	0.0022578	C5orf45
0.000436	0.0489	668.693298	275.36084	-1.383312	0.0116635	ACPL2
0.000001	0.000004	5055.02783	2017.40063	-1.367021	0.0049193	WSB1
0.000001	0.000002	6532.86084	3281.38354	-1.004964	0.0264476	PRKCB
0.000006	0.000078	1725.18677	857.634277	-1.100563	0.0203822	TMEM150A
0.000039	0.010149	1016.66534	360.454987	-1.595031	0.0030591	FBP1
0.000218	0.012176	703.280884	348.981628	-1.124406	0.025532	CNPY4
0.000852	0.066924	558.641846	260.063019	-1.203667	0.0224641	ARHGEF6
0.000082	0.004948	859.449036	404.436249	-1.199909	0.0169537	HMGA2
0.000594	0.080038	583.796448	251.457993	-1.31246	0.0135552	CCBL1
0.003282	0.193821	440.205597	209.388977	-1.153781	0.031395	FBXO9
0.000215	0.068267	705.377075	259.106903	-1.541534	0.0048543	CHD7
0.000061	0.037392	922.335571	288.746429	-1.767236	0.0017077	PHF15
0.00026	0.1173	680.222473	233.291824	-1.632499	0.0035527	ALK
0.001535	0.071119	593.229431	257.194672	-1.305039	0.0238098	STRIP2
0.000001	0.000006	3539.46265	1745.86426	-1.080469	0.0196359	PNISR
0.000001	0.000003	4446.07666	2226.78955	-1.042135	0.0228966	DGCR2
0.003405	0.143468	432.868835	223.730682	-1.040119	0.0465571	NA
0.000002	0.000015	2646.47412	1284.06116	-1.103712	0.0184075	C1RL
0.00246	0.140643	456.975342	224.686798	-1.112296	0.0344437	COL6A1
0.008266	0.96509	422.387756	94.655289	-2.179026	0.0031016	SEMA4B
0.000003	0.000028	2226.18262	1088.05774	-1.101896	0.0191601	GLT25D1
0.002494	0.101831	455.927246	239.984634	-1.020018	0.0485956	CTF1
0.00005	0.00248	957.971252	453.198059	-1.189437	0.0168386	PTPRB
0.000001	0.000007	5767.7417	1612.0083	-1.882432	0.0005174	RARB
0.000001	0.000008	6501.41748	1559.422	-2.095304	0.0002274	ANKZF1
0.000001	0.000011	3909.44507	1726.74194	-1.241036	0.0094983	RCN3
0.000006	0.000633	1797.50623	571.756226	-1.75406	0.0011975	OBSCN
0.000001	0.000005	3885.33838	1906.49146	-1.083776	0.0191134	HPS4
0.062992	0.921897	263.075256	106.128662	-1.306875	0.0283172	CEP152
0.000394	0.020391	628.865173	320.298218	-1.085221	0.0316528	PI4K2B
0.014423	0.496039	340.635284	160.627167	-1.131764	0.039787	SEMA4G
0.000612	0.143488	580.652161	223.730682	-1.462515	0.0076709	KIAA0895L
0.000006	0.002541	1773.39966	451.285828	-2.077767	0.0003624	FAM178A
0.000152	0.143487	755.686279	223.730682	-1.836222	0.0015515	MESP1
0.000047	0.001959	974.740967	471.364227	-1.156836	0.0192011	KIFC2
0.00004	0.00123	1174.92969	509.608795	-1.31055	0.0114157	ASB16-AS1
0.000192	0.024852	721.098694	309.780945	-1.325096	0.0111818	TIGD7
0.000003	0.000026	2275.4436	1105.26782	-1.109674	0.0184123	WSB2

0.000032	0.011774	1071.16699	350.89386	-1.706474	0.0019085	previous version conserved probe
0.000005	0.000078	1989.31006	858.590393	-1.296613	0.008353	FBXO46
0.000001	0.000011	3457.71021	1421.74158	-1.344514	0.0057785	CELSR2
0.	0.000001	13350.8066	5744.33301	-1.185784	0.0107589	TCEA1
0.000003	0.000046	2350.90747	964.719055	-1.355186	0.0059808	SLC2A1
0.000002	0.000029	2660.09961	1074.67224	-1.367488	0.0054906	PFKFB3
0.000016	0.000738	1283.93298	556.458374	-1.311499	0.0088177	previous version conserved probe
0.00008	0.002573	863.641479	450.329712	-1.053595	0.0311387	previous version conserved probe
0.001959	0.090271	474.793182	245.721313	-1.046417	0.0430071	NA
0.00004	0.000754	1047.06042	554.546143	-1.021758	0.034452	DHRS13
0.011657	0.391615	354.260712	174.012756	-1.0821	0.0465722	ERO1L
0.000001	0.000016	5623.10254	1258.24609	-2.204183	0.0001603	CYP24A1
0.000002	0.000009	3309.927	1519.26526	-1.18568	0.012139	CCNL2
0.000133	0.197698	775.600342	208.432861	-1.968288	0.0009641	TMEM121
0.000082	0.043559	859.449036	281.097534	-1.705767	0.0022354	previous version conserved probe
0.001484	0.186233	497.851562	211.301208	-1.318474	0.0149708	PDGFA
0.00013	0.377905	779.792786	175.924988	-2.204876	0.0004545	INSIG1
0.000392	0.02485	629.913269	309.780945	-1.134041	0.0260641	ZNF766
0.000534	0.081655	603.710571	250.501877	-1.365843	0.011234	previous version conserved probe
0.000001	0.000001	10061.8428	4196.38477	-1.264997	0.0074711	AKR1C3
0.004736	0.628808	409.810455	145.32933	-1.543712	0.0079983	previous version conserved probe
0.000001	0.000002	6932.18994	2991.68091	-1.221511	0.0094299	KANK2
0.000063	0.063605	910.806335	395.831207	-1.311016	0.0240479	AKR1C1|AKR1C3|LOC101060798|AKR1C4
0.000002	0.000008	3166.33594	1535.51917	-1.106085	0.0176946	VPS13C
0.000008	0.000104	1608.84668	806.00415	-1.094096	0.0213555	FAM189B
0.	0.000001	11255.6377	4401.94922	-1.350199	0.004962	TUBB
0.001221	0.265291	514.621338	194.091156	-1.480879	0.0079637	ZNF700
0.000122	0.011583	789.225769	351.849976	-1.274334	0.0130187	JUNB
0.000025	0.001755	1141.39026	479.969269	-1.355413	0.0076817	PNKD
0.	0.000001	22925.2793	9912.99023	-1.157723	0.0121041	NREP
0.001037	0.218279	529.294861	203.652298	-1.456354	0.0084972	ZFP62
0.000009	0.075617	1520.80554	254.32634	-2.658797	7.349E-05	FANCF

0.000077	0.030296	869.930115	301.175934	-1.62679	0.003019	LOC100996872
0.000031	0.000472	1247.24927	602.351868	-1.154904	0.0207812	MMP15
0.186294	0.997645	210.66983	69.796326	-1.570504	0.0169636	SLFN5
0.035136	0.946349	292.422302	100.391975	-1.545116	0.0118492	STAMBPL1
0.000947	0.260206	537.679688	195.047272	-1.537276	0.006224	ERCC6
0.000074	0.300611	879.363098	188.354462	-2.280787	0.0003263	RTP4
0.029899	0.696451	300.807159	137.68042	-1.154696	0.0407838	ZNF280B
0.000642	0.006382	981.029602	389.138428	-1.438642	0.0206798	previous version conserved probe
0.000001	0.000004	4623.20703	1998.27832	-1.25815	0.0082751	GNAI2
0.003664	0.377908	427.628296	175.924988	-1.347322	0.0152938	MMD
0.000664	0.027727	641.442444	304.044281	-1.186116	0.0273051	HMGXB4
0.000052	0.000749	1620.37585	753.417847	-1.204696	0.0266467	FBXO2
0.000045	0.001177	1052.30103	513.433228	-1.140403	0.0217867	EDEM1
0.000005	0.000066	2021.80139	890.142212	-1.265459	0.0101479	TTLL4
0.066573	0.951271	262.02713	98.479744	-1.408601	0.0224883	ZNF680
0.001311	0.17541	508.332672	214.169556	-1.330222	0.0140709	GLCE
0.000004	0.000041	2079.44751	989.578064	-1.147082	0.0159103	GLE1
0.000031	0.000849	1076.40747	543.072754	-1.091915	0.0243874	MAP3K12
0.000019	0.000437	1214.75781	610.956909	-1.096222	0.0229561	NA
0.000192	0.036691	721.098694	289.702545	-1.418275	0.0077149	TMEM168
0.000003	0.000031	2275.4436	1062.24268	-1.166849	0.0141782	POLRMT
0.000006	0.000064	1775.49597	893.966614	-1.07879	0.0223232	MED20
0.000051	0.002324	956.923157	457.978638	-1.172718	0.0181082	PAPLN
0.000015	0.000226	1313.28003	696.051025	-1.018681	0.0315412	INF2
0.000608	0.103953	582.748352	239.028519	-1.378353	0.0106152	ACAD10
0.00157	0.077307	823.813354	344.20105	-1.364906	0.0466924	FAM86DP|FAM86HP|FAM86B1|FAM86FP|FAM86C1|FAM86JP|FAM86A|FAM86EP
0.000005	0.000052	1871.92188	938.903992	-1.079294	0.0220152	previous version conserved probe
0.000001	0.00001	6831.57178	1477.19617	-2.242977	0.000135	FAM102A
0.000001	0.000002	5127.34717	2559.51733	-1.030185	0.0239582	GOLGA8B|GOLGA8A
0.008634	0.405642	371.030426	172.100525	-1.166166	0.0329899	TUBGCP5
0.000056	0.004001	935.960938	418.777954	-1.270772	0.0120822	GDF6
0.000018	0.002892	1232.57568	441.724701	-1.586682	0.0028209	LOC151009
0.000238	0.028768	691.751648	302.13205	-1.301729	0.0126038	previous version conserved probe
0.008934	0.39854	368.934204	173.056641	-1.150213	0.03503	MGC57346
0.003558	0.140622	429.724518	224.686798	-1.023874	0.0496358	PWWP2A
0.	0.000001	13255.4287	6263.50293	-1.045502	0.0210925	AHNAK2
0.000001	0.000007	6239.39014	1621.56946	-1.982304	0.0003469	FAM178A

0.000588	0.022293	584.844604	315.517639	-1.001768	0.0456057	PPP6R2
0.015196	0.419904	362.645569	170.188309	-1.147089	0.0488478	LOC643696
0.000048	0.010328	968.452332	358.542755	-1.534454	0.0040028	ZC3H12D
0.001894	0.520146	482.129944	157.75882	-1.667301	0.0046114	FUT11
0.000007	0.000138	1626.66455	761.066772	-1.195281	0.0135448	LRRC8E
0.000303	0.088466	660.308411	246.677429	-1.515497	0.0056486	ZFP62
0.000005	0.00004	1941.09705	998.183044	-1.03866	0.0262332	MST1|MST1P2
0.000001	0.000017	5153.5498	1249.64111	-2.093824	0.0002381	HILPDA
0.059145	0.907487	267.2677	108.997002	-1.294423	0.0299924	RORC
0.000007	0.000061	1703.17639	906.396118	-1.000553	0.0319665	VHLL
0.004594	0.338935	411.906677	181.661667	-1.249364	0.0224942	GNL3L
0.000001	0.000039	4254.27295	1001.05139	-2.151462	0.0002029	previous version conserved probe
0.000048	0.007036	968.452332	390.094543	-1.417177	0.0066015	FAM86C1|FAM86DP|FAM86B1|FAM86FP|FAM86HP
0.000003	0.002414	2347.76318	455.110291	-2.45494	9.55E-05	SKP2
0.009261	0.528292	366.838013	156.802704	-1.275189	0.0227124	ERGIC2
0.084744	0.912454	253.642273	108.040886	-1.22665	0.0451179	MYL2
0.025647	0.679663	309.192017	139.592651	-1.177111	0.0371487	previous version conserved probe
0.000981	0.044404	534.5354	280.141418	-1.037773	0.0415678	NARG2
0.03812	0.965079	288.229858	94.655289	-1.608418	0.0099233	MEX3A
0.000014	0.000198	1339.48279	708.48053	-1.020929	0.031316	LOC727849|LOC388152
0.000001	0.000011	3388.53491	1418.87329	-1.318136	0.0065465	NA
0.422673	0.998445	168.745483	64.059639	-1.353719	0.0448493	FAM86A|FAM86C2P|FAM86C1|FAM86DP|FAM86HP|FAM86EP|FAM86JP
0.00001	0.00123	1517.66125	509.608795	-1.680521	0.0018275	PP7080
0.000033	0.001345	1057.5415	501.959869	-1.180292	0.0167918	PAG1
0.00031	0.071468	661.356506	258.150787	-1.456366	0.0072844	CEP44
0.003946	0.611851	422.387756	147.241562	-1.570511	0.0070589	CBX2
0.000503	0.205185	601.614319	225.642914	-1.50202	0.0099508	PPM1A
0.000011	0.000498	1435.90869	596.615173	-1.37235	0.0064871	HNRNPA3
0.00003	0.051844	1084.79236	272.492493	-2.076366	0.000502	HNRNPA2B1
0.	0.000003	12703.0762	2154.125	-2.560211	4.695E-05	MALAT1
0.012229	0.891443	351.116364	111.865341	-1.66714	0.0065402	ZEB1-AS1
0.000001	0.000006	3895.81958	1728.65417	-1.234319	0.0094767	C16orf46
0.003664	0.371198	427.628296	176.881104	-1.340001	0.015702	TMOD2
0.000015	0.001385	1314.32812	567.931763	-1.315781	0.0095268	CCSAP
0.001658	0.52829	488.41861	156.802704	-1.694172	0.0039471	previous version conserved probe

0.000002	0.000018	3213.50098	1226.69434	-1.450853	0.0036178	LOC642852
0.006106	0.351576	393.04071	179.749435	-1.193574	0.0285848	SMAD2
0.000003	0.000017	2556.33691	1248.68494	-1.09554	0.0200299	DPP9
0.035852	0.800006	291.374176	125.250938	-1.235232	0.0321614	CCR6
0.000238	0.017988	691.751648	328.903229	-1.183675	0.0204208	previous version conserved probe
0.000001	0.00001	6293.89209	1473.3717	-2.132306	0.0002002	HK2
0.000011	0.007394	1440.1012	391.050659	-1.983598	0.0005826	GPCPD1
0.321772	0.997648	183.419006	69.796326	-1.357938	0.0365712	C20orf118
0.	0.000001	10830.1055	5163.97217	-1.056279	0.0201878	FASN
0.001637	0.07102	489.466705	257.194672	-1.028543	0.0451859	B4GALNT4
0.00073	0.029858	562.83429	300.219818	-1.015709	0.0440355	previous version conserved probe
0.00008	0.02897	862.593384	302.13205	-1.610533	0.00321	ZBTB3
0.000112	0.004172	803.899292	415.909607	-1.067526	0.0302679	NLRP2
0.000001	0.000006	3712.40063	1702.83911	-1.186966	0.0118632	TG
0.156637	0.978657	219.054688	88.91861	-1.281062	0.0372602	previous version conserved probe
0.000001	0.000001	7849.28516	3627.49683	-1.121313	0.0150536	TMED1
0.000001	0.000007	3428.36304	1595.75439	-1.165896	0.0132399	SPINT3
0.000051	0.063071	954.826904	262.931366	-1.945385	0.0008663	ZNF500
0.000004	0.000064	2005.03174	893.966614	-1.247527	0.0101179	PLEKHG3
0.000086	0.03884	850.016052	286.834229	-1.662234	0.0026566	TXLNG2P
0.000812	0.095865	552.35321	242.852966	-1.279875	0.0159937	ZNF341
0.000002	0.000025	3166.33594	1115.78516	-1.565885	0.0021829	IGF2BP3
0.005119	0.688042	404.569916	138.636536	-1.588336	0.0069734	GMPR
0.000074	0.001616	950.63446	486.662048	-1.075574	0.0302358	PRSS53
0.000019	0.000065	1828.94946	892.054382	-1.123139	0.0252695	C16orf74
0.000004	0.000009	3230.27075	1525.00195	-1.144951	0.0187023	ANKRD9
0.000003	0.000025	2536.42285	1115.78516	-1.24673	0.0097016	previous version conserved probe
0.000085	0.003945	853.1604	419.73407	-1.138008	0.022003	OR1N2
0.000063	0.082496	908.710144	250.501877	-1.94154	0.0009188	OR52A5
0.000003	0.000832	2407.50537	544.984985	-2.228986	0.0001887	C19orf70
0.000038	0.00119	1022.95398	512.477112	-1.103352	0.023646	OR4K17
0.000004	0.00008	2015.51282	852.85376	-1.324738	0.0071383	OR4K5
0.000065	0.003173	904.5177	435.031891	-1.168315	0.0188791	RPL13AP3
0.000001	0.000007	3523.74097	1642.604	-1.163993	0.0133042	TNFAIP8L3
0.001244	0.45107	518.813721	166.363846	-1.700817	0.0039874	ZNF285
0.000001	0.000006	5034.06543	1721.96143	-1.598417	0.0017317	IER5L
0.00023	0.008263	695.944092	371.928375	-1.021183	0.0389437	C9orf37
0.003466	0.594906	449.63858	149.153793	-1.642942	0.0064702	C5orf54
0.000264	0.008808	679.174377	368.103912	-1.001429	0.042754	PLEKHO2
0.000012	0.0008	1689.55103	548.809448	-1.727383	0.0018868	C8orf58
0.000018	0.001818	1255.63403	477.100922	-1.501971	0.0039931	FAM69A
0.000176	0.027222	733.676025	305.000397	-1.370914	0.0092041	C20orf96

0.000143	0.885964	766.167358	112.821457	-2.781815	0.0001064	previous version conserved probe
0.000017	0.000494	1258.77844	597.571289	-1.179736	0.0157551	ESRRA
0.000008	0.000404	1643.4342	619.56189	-1.512846	0.0034517	BLOC1S5|EEF1E1-MUTED
0.000002	0.000007	3284.77222	1595.75439	-1.103893	0.0177808	NCAPD2
0.000001	0.000001	10264.127	4856.10352	-1.073753	0.0186154	WDFY1
0.000014	0.000261	1331.0979	672.148193	-1.090097	0.0229086	CXCL2
0.000001	0.000002	8490.72754	3203.93823	-1.411276	0.0037999	STRADB
0.000003	0.000607	2406.45728	576.536804	-2.146928	0.0002481	MMP11
0.34172	0.998667	180.274673	65.97187	-1.412703	0.0321884	SLC25A15
0.004988	0.852967	406.666138	117.602028	-1.818957	0.0033476	CCL7
0.000023	0.00813	1161.30432	372.884491	-1.738734	0.0016007	IFI44
0.000001	0.000008	4027.88135	1546.99255	-1.444125	0.0035718	DYNLT3
0.000004	0.00003	2117.17944	1100.4873	-1.015864	0.0296559	MST1
0.000002	0.000017	2924.2229	1234.34326	-1.305201	0.0071719	PWP2
0.000001	0.000005	4236.45508	1806.09949	-1.289107	0.0072366	previous version conserved probe
0.001044	0.004057	898.229065	417.821838	-1.21638	0.0446042	previous version conserved probe
0.000001	0.000006	3479.72046	1764.0304	-1.040298	0.0238321	previous version conserved probe
0.00008	0.052862	863.641479	271.536377	-1.760694	0.0018161	C1QTNF6
0.	0.000001	10908.7139	4647.67041	-1.224989	0.0090009	ANP32A
0.000007	0.000922	1703.17639	535.423889	-1.774267	0.0011328	HIC1
0.000007	0.011211	1647.62671	353.762207	-2.317529	0.0001737	IFIT1
0.000254	0.110396	683.366821	236.160172	-1.622444	0.0036787	CCNL2
0.000098	0.00278	828.005798	444.593048	-1.013196	0.0374816	UBA5
0.000011	0.004877	1420.18713	405.392365	-1.913387	0.0007355	ARHGAP5
0.000025	0.000627	1133.00537	572.712341	-1.089075	0.0242352	DTWD1
0.000003	0.000184	2357.19629	718.997742	-1.795831	0.0009119	previous version conserved probe
0.000002	0.000013	2673.7251	1329.95459	-1.06799	0.0216612	TUT1
0.000214	0.015158	707.473267	336.552155	-1.18315	0.0201449	SAA1
0.039718	0.807376	286.133636	124.29483	-1.218481	0.0345627	C21orf49
0.000001	0.000007	3567.76172	1670.3313	-1.157881	0.0136651	C1orf233
0.000009	0.005901	1544.91199	392.962891	-2.07836	0.0003907	previous version conserved probe
0.000005	0.000054	1863.53711	931.255066	-1.085221	0.0214497	TNFSF12
0.000001	0.000001	9854.31641	4842.71777	-1.020005	0.0241168	ADSL
0.000007	0.000132	1697.93591	767.759583	-1.243	0.0108088	TMEM234
0.000153	0.023965	754.638184	311.693176	-1.380145	0.0087108	CSTF3

0.000003	0.000028	2294.30957	1091.8822	-1.138679	0.0161064	SNAPC3
0.000001	0.000001	8852.3252	3622.71606	-1.294114	0.0065613	EIF4E2
0.001665	0.182572	489.466705	212.257324	-1.287948	0.0171588	LNX1
0.025505	0.603365	309.192017	148.197678	-1.096082	0.0484985	CEP152
0.002673	0.897004	450.686676	110.909233	-2.047606	0.001425	WHSC1
0.000002	0.000007	3352.89941	1673.19958	-1.065399	0.0212053	ASPH
0.000071	0.004814	887.747986	406.34848	-1.238676	0.0141826	PRKCB
0.00361	0.280959	428.676422	191.222809	-1.238208	0.0226693	TGM2
0.019925	0.956443	322.817444	97.523636	-1.734762	0.0058403	RGS4
0.000002	0.000012	2966.14722	1370.11145	-1.175563	0.0129522	DPM3
0.000006	0.000054	1775.49597	928.386719	-1.022638	0.0292698	PDE7A
0.	0.000001	14563.4688	5601.87207	-1.344087	0.0050683	FST
0.003078	0.265268	440.205597	194.091156	-1.256263	0.0207779	YPEL1
0.000331	0.146432	649.827332	222.774582	-1.630164	0.0037295	C2orf68
0.000018	0.000512	1247.24927	593.746826	-1.175729	0.0160849	NPB
0.000001	0.000002	6913.32422	2935.27026	-1.244978	0.0084391	LEPROT
0.000564	0.009527	879.363098	363.323334	-1.381021	0.0203297	PGBD3
0.000028	0.000557	1108.89893	585.141785	-1.026916	0.0319059	CDC20
0.000013	0.000551	1383.5033	603.307983	-1.302466	0.00912	previous version conserved probe
0.000001	0.000003	5252.07227	2427.57349	-1.142585	0.0140436	TFPI
0.000312	0.669609	657.164062	131.943741	-2.354762	0.0006728	DIDO1
0.000004	0.000032	2023.89771	1049.81323	-1.021679	0.0281043	PHF14
0.086975	0.982179	247.353622	87.006378	-1.498872	0.0168337	KLK3
0.000014	0.000703	1327.95361	561.238953	-1.347845	0.007478	PCDH1
0.000306	0.012358	659.260315	350.89386	-1.026244	0.0394812	BRWD1
0.000301	0.028774	661.356506	302.13205	-1.238857	0.0166633	IL36RN
0.000099	0.003185	825.909546	435.031891	-1.041113	0.0334038	previous version conserved probe
0.000011	0.011876	1422.28333	395.831207	-1.948707	0.0007313	previous version conserved probe
0.000131	0.953223	783.985229	98.479744	-3.004671	6.763E-05	previous version conserved probe
0.000001	0.000007	3605.49341	1672.24353	-1.171478	0.0128055	RPL9
0.062996	0.8357	263.075256	120.470367	-1.13518	0.0488173	ZNF662
0.000043	0.002607	993.606934	449.373596	-1.252802	0.0126626	NOVA1
0.001049	0.087447	528.246704	247.633545	-1.189368	0.0234606	AGER
0.000113	0.060627	801.80304	264.843597	-1.69122	0.0024914	ECE2
0.000023	0.00213	1157.11182	464.671417	-1.422066	0.0057584	SNORD52
0.000003	0.02739	2284.87671	478.057037	-2.346066	0.0002064	HCP5
0.000076	0.137802	933.864746	225.642914	-2.120922	0.0006257	NSUN5
0.000004	0.000098	2190.54688	816.521423	-1.505022	0.0032081	TMEM50B
0.000005	0.003348	1947.38574	431.207428	-2.273525	0.000181	COL11A1
0.000002	0.00001	2973.48413	1447.55664	-1.099966	0.0183762	HMGCL
0.006296	0.603359	390.944489	148.197678	-1.446785	0.011738	CAPRIN1
0.000114	0.006757	800.754944	384.357849	-1.171746	0.0197077	ATXN3

0.000229	0.018023	696.992188	326.990997	-1.202343	0.0187123	TTK
0.000017	0.391579	1254.58594	174.012756	-2.893276	4.874E-05	SNHG3
0.000259	0.004291	786.081421	413.997375	-1.042576	0.0422459	ABCC3
0.000683	0.026732	571.219177	305.956482	-1.010655	0.0448382	ADAMTS2
0.01769	0.536485	329.106079	155.846588	-1.121219	0.0423871	SYNPO
0.028163	0.835703	303.951477	120.470367	-1.351119	0.0210867	LRCH1
0.000007	0.006547	1623.52014	719.953857	-1.275024	0.0164472	RACGAP1
0.000305	0.000369	1475.73682	630.079163	-1.332913	0.0277358	SEPSECS
0.00233	0.238218	461.167786	203.652298	-1.258207	0.0224701	S1PR5
0.000445	0.075584	616.287842	283.009766	-1.228273	0.0236092	PIGX
0.000018	0.000129	1707.3689	771.584045	-1.243304	0.0148187	ZNF692
0.000003	0.000023	2326.80103	1148.29297	-1.085659	0.0204605	TMEM48
0.000618	0.112663	599.518127	235.204056	-1.440941	0.0090598	ALG13
0.000083	0.000437	1445.34167	610.956909	-1.347436	0.0156331	CCM2
0.000018	0.008391	1238.86438	370.97226	-1.839331	0.0010507	KBTBD7
0.000002	0.001632	3356.0437	1416.00488	-1.307088	0.0122046	NAV1
0.003214	0.260201	437.061279	195.047272	-1.239316	0.0222391	KLHL13
0.000001	0.000006	3520.59668	1699.97083	-1.112616	0.0169843	PWWP2A
0.001749	0.240749	485.274261	198.871719	-1.363569	0.0130418	SSX2IP
0.000273	0.016843	673.933838	330.81546	-1.139105	0.0246372	SENP8
0.000137	0.00429	771.407898	413.997375	-1.016212	0.038025	TNFAIP8L1
0.000331	0.011776	649.827332	350.89386	-1.005518	0.042786	previous version conserved probe
0.000906	0.792499	541.872131	126.207054	-2.13756	0.0008538	FAM13C
0.000002	0.000064	2572.05835	895.878845	-1.589995	0.0020771	C17orf58
0.000155	0.000249	1311.18384	677.884888	-1.055678	0.0497988	FAM149B1
0.000004	0.000899	2039.61926	624.342468	-1.802094	0.0010195	KIAA0754
0.005749	0.398532	397.233154	173.056641	-1.260692	0.0223493	LOC643923
0.000002	0.000011	2907.45312	1411.22437	-1.104026	0.0180976	C7orf73
0.000006	0.000131	1791.21753	769.671814	-1.313529	0.007767	TXNRD3
0.000003	0.00003	2507.07568	1071.80383	-1.288488	0.0079722	previous version conserved probe
0.001164	0.205742	518.813721	206.52063	-1.408698	0.0103194	PPP1R3E
0.000044	0.001157	988.366394	515.345459	-1.04715	0.0304298	KLHDC10
0.000018	0.000292	1262.97083	657.806458	-1.045548	0.0285195	previous version conserved probe
0.000001	0.000007	3542.60693	1644.51611	-1.170056	0.0129233	BRD3
0.000087	0.002157	1134.05347	463.715302	-1.395953	0.0109052	MANEA
0.000005	0.004351	1865.6333	413.04126	-2.275051	0.0001842	AGPAT5
0.000001	0.000007	4054.08398	1611.05212	-1.395094	0.0044606	GJB4
0.000021	0.000415	1190.65137	616.693542	-1.053723	0.0277916	C3orf17
0.000128	0.004605	781.888977	409.216827	-1.051244	0.032754	PDGFD
0.074785	0.9184	254.690384	101.348091	-1.323601	0.034383	RINL
0.000002	0.000005	3573.0022	1856.77344	-1.001903	0.0318043	ACTR2
0.000011	0.000139	1421.23523	760.110657	-1.002012	0.0331227	WIPF1
0.027498	0.928987	328.057983	103.260315	-1.676372	0.013231	MARCKS

0.103616	0.937835	238.96875	102.304207	-1.212279	0.0424159	previous version conserved probe
0.000063	0.027227	911.854492	305.000397	-1.675096	0.0024052	KIAA1841
0.009409	0.371189	365.789886	176.881104	-1.10789	0.0409347	EPHA5
0.000023	0.000433	1156.06372	611.912964	-1.022376	0.0321055	PLSCR1
0.002394	0.457087	459.071564	165.40773	-1.532689	0.0073774	PIGK
0.000002	0.000008	3270.09888	1534.56311	-1.153709	0.0141118	SOS1
0.001359	0.086786	505.188324	247.633545	-1.125182	0.0307538	IFI44L
0.000013	0.000381	1348.91577	626.2547	-1.211845	0.0133599	SMAD2
0.000002	0.000012	2899.06836	1394.97046	-1.116522	0.0173436	CSTF2T
0.00001	0.000418	1478.88123	758.198425	-1.063224	0.027815	RECQL
0.000731	0.028774	562.83429	302.13205	-1.00688	0.0455982	VSIG10
0.000002	0.000007	3262.76196	1662.68237	-1.034988	0.0245032	ECT2
0.000722	0.041921	563.882385	283.009766	-1.100471	0.0316414	FAM76B
0.000071	0.015157	885.651733	336.552155	-1.497421	0.0049114	PAFAH2
0.000002	0.000023	2769.10278	1151.16138	-1.326678	0.0065488	TM7SF3
0.000282	0.069632	669.741394	258.150787	-1.473997	0.0065487	PARD6G
0.000218	0.013462	705.377075	343.244934	-1.151617	0.0229442	FEM1C
0.0017	0.308861	486.322388	186.442245	-1.453853	0.0092527	WNT5A
0.000005	0.000046	1937.95276	964.719055	-1.087092	0.0210872	LYST
0.000142	0.241465	766.167358	234.24794	-1.79344	0.0045891	PRTG
0.000812	0.047058	552.35321	277.273071	-1.099238	0.0321771	PHF6
0.037738	0.860386	409.810455	106.128662	-1.969524	0.0368557	ZNF404
0.000003	0.000032	2192.64307	1049.81323	-1.132823	0.0167289	GJC1
0.00091	0.866785	543.968323	114.733688	-2.272218	0.0006235	ITGB8
0.000005	0.001057	1932.71216	522.994385	-1.983728	0.000487	PDP1
0.000001	0.000012	5771.93408	1376.8042	-2.110541	0.0002194	INSIG1
0.000295	0.018994	665.54895	324.12265	-1.150009	0.0238403	RNF24
0.003248	0.297467	443.349915	188.354462	-1.307026	0.0183024	SERBP1
0.000016	0.000824	1289.17358	545.941101	-1.345003	0.0076127	UBL4A
0.000001	0.000003	6027.67236	2397.93408	-1.350916	0.0051949	PLEKHA2
0.000175	0.137655	734.724121	226.59903	-1.779655	0.0019726	ZNF74
0.021001	0.897011	319.673126	110.909233	-1.53852	0.0108446	NAPEPLD
0.000007	0.000941	1689.55103	533.511658	-1.768298	0.001164	PFKFB2
0.000245	0.178327	687.559204	219.906235	-1.727328	0.0028207	ZC3H6
0.000001	0.000039	3448.2771	1001.05139	-1.848418	0.0006595	RNF141
0.000029	0.058288	1092.12915	266.755829	-2.115695	0.0004388	ADPRH
0.000011	0.001423	1522.90173	497.179321	-1.721241	0.001594	ATP8B2
0.001164	0.275641	518.813721	192.178925	-1.505922	0.0072113	RHNO1
0.008634	0.47248	371.030426	163.495499	-1.235558	0.025819	GABRB3
0.006285	0.291875	394.088837	189.310577	-1.127548	0.0371595	SLC35D1
0.000048	0.000971	1451.63037	530.643311	-1.557687	0.0058848	SFT2D3
0.000031	0.000633	1073.26318	571.756226	-1.013196	0.0341805	OSTM1
0.000015	0.012215	1301.75085	504.828217	-1.472349	0.0067429	previous version conserved probe
0.000906	0.137802	541.872131	225.642914	-1.351614	0.0123418	PARP11
0.00004	0.001271	1010.37665	506.740448	-1.102336	0.0238591	SMC2
0.000821	0.103916	551.305115	239.028519	-1.298586	0.014909	GNA13
0.000318	0.001635	1052.30103	485.705933	-1.220752	0.0315554	PPIP5K2
0.000004	0.000044	2111.93872	975.236328	-1.190298	0.0129506	ZYG11B

0.00271	0.197718	449.63858	208.432861	-1.190454	0.0260555	ARRDC4
0.000016	0.000354	1273.4519	634.859741	-1.108882	0.0213916	IGSF3
0.000001	0.000005	3630.64819	1856.77344	-1.025096	0.0253235	FAM46A
0.000169	0.115561	739.964661	308.824829	-1.365528	0.0213286	SIKE1
0.000009	0.000311	1501.93958	650.157532	-1.312952	0.0082584	ITGA4
0.000001	0.000001	7618.70117	3761.35278	-1.026894	0.0236874	BCAT1
0.000014	0.000444	1326.90552	609.044678	-1.228395	0.0124976	RDH10
0.000015	0.000071	1782.83264	875.800476	-1.115027	0.0243251	previous version conserved probe
0.00056	0.093958	590.085144	243.809082	-1.369596	0.0107856	ANO7
0.001744	0.611851	484.226166	147.241562	-1.766996	0.0031181	CRYBG3
0.094862	0.942711	243.161194	101.348091	-1.252514	0.036405	KATNAL2
0.000998	0.535771	538.727783	155.846588	-1.843445	0.0023289	TWSG1
0.000018	0.000391	1250.39355	623.386353	-1.108865	0.0215331	SCAF11
0.00006	0.001734	921.287415	480.925354	-1.048864	0.0309818	LETM1
0.002065	0.083314	470.600739	249.545761	-1.012799	0.0490174	TMEM56
0.000043	0.002028	993.606934	468.49588	-1.19249	0.016358	PHKA1
0.000567	0.041119	607.902954	283.965881	-1.203883	0.0218128	CSNK1E
0.115566	0.954098	233.72821	92.743065	-1.319806	0.0376376	SLC35B4
0.000001	0.000005	3668.38013	1862.51013	-1.035459	0.0241046	PPP1R3B
0.000002	0.000011	2691.54272	1393.05823	-1.010862	0.0281823	IL17RD
0.000198	0.027731	716.90625	304.044281	-1.342929	0.0104491	ERCC6
0.000005	0.000284	2118.22754	661.63092	-1.770622	0.0011795	NUFIP2
0.011319	0.702762	361.597473	136.724304	-1.439286	0.0149224	SLC16A12
0.000138	0.303495	770.359802	265.799713	-1.63038	0.0397334	PLSCR4
0.001248	0.993584	520.909973	77.445236	-2.770036	0.000216	ZSWIM1
0.000354	0.0227	641.442444	314.561523	-1.13883	0.0252962	ZNF345
0.00001	0.897041	1465.25574	110.909233	-3.727633	1.058E-05	CIRBP
0.000027	0.003216	1109.94702	434.075775	-1.460492	0.0050083	RNF187
0.085112	0.930734	248.401733	104.216431	-1.244971	0.0364731	FXN
0.000213	0.00764	706.425171	376.708923	-1.024338	0.0382105	RNASEH2C
0.000002	0.000006	3338.22583	1722.91748	-1.015456	0.0267445	TNK2
0.000046	0.002296	979.981506	458.934753	-1.203013	0.0157365	previous version conserved probe
0.000001	0.000002	5588.51514	2611.14746	-1.11904	0.0156102	PTRF
0.000348	0.017404	648.779236	328.903229	-1.093191	0.0306299	NASP
0.000126	0.013692	789.225769	342.288818	-1.312643	0.0112891	RNF213
0.002027	0.083304	472.69696	249.545761	-1.019191	0.0478891	MAF
0.000156	0.19382	751.493835	209.388977	-1.91793	0.001177	ZSCAN2
0.000071	0.008776	885.651733	369.060028	-1.369207	0.0082841	TMEM144
0.000651	0.028771	574.363525	302.13205	-1.036041	0.0402683	MEGF6
0.000001	0.000006	4274.18701	1751.60095	-1.347217	0.0055182	POLR1A
0.000009	0.000121	1588.93262	782.101318	-1.120838	0.019582	WDR52
0.012927	0.907485	346.92395	108.997002	-1.684369	0.006151	AKT2
0.000008	0.001034	1587.88452	524.906616	-1.703094	0.0015523	MTR
0.000008	0.000134	1594.1731	765.847351	-1.156811	0.0161762	HGSNAT
0.00591	0.639948	395.136932	142.460999	-1.516207	0.0107331	previous version conserved probe

0.000117	0.005814	796.5625	393.918976	-1.130235	0.023456	STX17
0.000001	0.000005	4027.88135	1803.23108	-1.219378	0.0100828	NFATC2IP
0.036592	0.921893	290.32608	106.128662	-1.454254	0.0159127	C2orf72
0.000009	0.000198	1550.15259	708.48053	-1.23206	0.0116682	C5orf24
0.000004	0.000235	2097.26538	686.489868	-1.70244	0.0013865	CALM3
0.000084	0.002324	855.256592	457.978638	-1.01557	0.0366194	TPD52
0.000001	0.000002	5714.28809	2835.83423	-1.029947	0.0237391	TK2
0.322882	0.998435	183.419006	66.927986	-1.418479	0.0322154	METTL8
0.000002	0.000018	2579.39526	1226.69434	-1.133588	0.0161147	BTRC
0.00004	0.002476	1012.4729	453.198059	-1.266853	0.011829	CPEB1
0.000413	0.08671	623.624634	247.633545	-1.428071	0.0082563	ACVR2B
0.006435	0.152405	509.380768	220.86235	-1.291616	0.0423709	CEP85
0.000003	0.000054	2441.04492	930.29895	-1.461402	0.0038004	IMPACT
0.000041	0.052572	1003.03992	441.724701	-1.290869	0.0261099	ETV1
0.000029	0.001868	1090.03296	475.18869	-1.303355	0.0097959	CDK12
0.00002	0.001391	1203.22864	499.091522	-1.375133	0.0068946	CRB2
0.002952	0.222635	468.504547	202.696182	-1.287263	0.0216555	TMF1
0.01293	0.637324	346.92395	144.373215	-1.303622	0.0216524	STAM2
0.011612	0.679633	355.308807	139.592651	-1.384936	0.0165877	CTBS
0.000011	0.000237	1414.94653	684.577637	-1.15116	0.017186	BBS10
0.000013	0.000448	1371.97412	608.088562	-1.278948	0.0099386	ERO1L
0.001815	0.081666	487.370483	250.501877	-1.057988	0.0419289	DMXL2
0.000444	0.028774	615.239746	302.13205	-1.13491	0.0263012	SLC9B2
0.000017	0.000267	1254.58594	669.279846	-1.010887	0.0329358	SPAST
0.00001	0.00055	1457.91907	586.0979	-1.420079	0.0052374	SOCS6
0.000005	0.000052	2596.16504	939.860107	-1.53171	0.003721	EEF2K
0.000003	0.000021	2242.95239	1168.37134	-1.009761	0.029567	DCK
0.001637	0.093957	489.466705	243.809082	-1.100742	0.0344097	RBM12B
0.000348	0.011209	747.301392	353.762207	-1.190429	0.0255459	NRP1
0.000002	0.000014	2747.09253	1321.34961	-1.116566	0.0176339	MRI1
0.022252	0.926059	316.528778	105.172546	-1.596419	0.0091582	UBN2
0.00002	0.000327	1203.22864	644.420898	-1.005261	0.0341593	PCDH7
0.	0.000001	11625.6201	4989.00293	-1.206426	0.0098054	ADAM9
0.000001	0.000003	5012.05518	2372.1189	-1.112689	0.0162768	COL4A4
0.000002	0.000009	3127.55591	1483.88904	-1.137477	0.0153221	C6orf89
0.000045	0.000197	1566.92236	709.436646	-1.245608	0.0189898	SRI
0.000247	0.009681	688.607361	362.367218	-1.042568	0.0360549	LMBR1
0.000004	0.000067	2106.69824	888.22998	-1.325742	0.0070429	SORL1
0.000001	0.000003	4573.9458	2226.78955	-1.081234	0.0190065	previous version conserved probe
0.000001	0.000002	6967.82568	3300.50562	-1.087701	0.0178076	MSL1
0.000009	0.00024	1518.70935	682.665405	-1.257608	0.0105824	CNOT6L
0.000027	0.004056	1112.04321	417.821838	-1.517862	0.0039494	SSPN
0.000011	0.002445	1420.18713	454.154175	-1.751309	0.0013569	ANGEL1
0.000003	0.000018	2357.19629	1223.82593	-1.011937	0.0285719	MCOLN3
0.000005	0.000198	1874.01819	708.48053	-1.499373	0.0033862	ERAP2
0.002362	0.119652	460.119659	232.335709	-1.076937	0.0391115	PGM2
0.	0.000001	16386.1289	7133.56689	-1.146065	0.0129249	GNPTAB
0.000393	0.032166	632.00946	296.395355	-1.20024	0.0201894	previous version conserved probe

0.000026	0.001315	1124.62048	503.872101	-1.26368	0.011443	PTAR1
0.00077	0.161901	557.59375	217.994003	-1.439329	0.0086642	FAM102B
0.001025	0.214	530.342957	204.608414	-1.452872	0.008593	CCNA2
0.	0.000001	34734.3164	11811.833	-1.509113	0.0023977	COL5A1
0.000009	0.000168	1510.32446	731.427246	-1.147062	0.0171577	UBIAD1
0.000003	0.000019	2371.86963	1205.65979	-1.042084	0.0249756	SEMA5A
0.000002	0.00001	2879.1543	1431.30273	-1.069509	0.0218767	CAV2
0.000003	0.000086	2334.1377	838.512024	-1.55364	0.0024963	CPSF6
0.000001	0.000002	5914.47656	2983.07593	-1.004727	0.0266546	NDFIP1
0.000005	0.000045	1922.23108	970.45575	-1.066976	0.0231339	ZNF346
0.129947	0.985221	228.487671	85.094154	-1.409186	0.0249578	MYEF2
0.000005	0.000152	1864.58521	746.725098	-1.414228	0.0049215	C9orf64
0.000008	0.000261	1586.83643	672.148193	-1.344111	0.0071971	SPCS3
0.000002	0.00002	2956.71436	1188.44983	-1.375773	0.0051658	FAM126A
0.000014	0.003682	1334.24219	424.514648	-1.758757	0.001364	TMEM19
0.00001	0.000216	1486.21802	697.007141	-1.195448	0.013909	UBTD2
0.008475	0.821776	373.126648	122.382599	-1.636311	0.0066614	CFHR1|CFH
0.000015	0.000267	1297.55847	669.279846	-1.059566	0.0263892	DHFR|DHFRL1
0.169326	0.99487	215.91037	75.533012	-1.494254	0.0215078	TRIM6-TRIM34|TRIM34
0.001296	0.201662	509.380768	207.476746	-1.376071	0.0118335	ZNF761
0.000002	0.000009	3045.80347	1514.48462	-1.069705	0.0210841	LOC653562|SLC6A10P|SLC6A8
0.000002	0.000013	3105.54565	1341.42798	-1.272582	0.0082322	EIF5A
0.000007	0.0013	1628.76074	504.828217	-1.796279	0.001062	FAM156A|FAM156B
0.033457	0.880173	296.614716	113.777573	-1.392019	0.0198153	FAM120C
0.000004	0.000095	2108.79443	822.258057	-1.441829	0.004188	FLAD1
0.000032	0.007882	1090.03296	374.796722	-1.640075	0.0025365	ATAT1
0.00004	0.073903	1011.4248	255.282455	-2.065832	0.0005546	ZNF783
0.000129	0.01416	1015.61719	340.376617	-1.673285	0.0044025	HMGN3
0.000168	0.019677	739.964661	322.210449	-1.306527	0.0118581	NR3C1
0.002317	0.412705	462.215881	171.144409	-1.49639	0.0083864	FAM178A
0.000612	0.02396	580.652161	311.693176	-1.00838	0.0446033	SEL1L
0.000144	0.009529	764.071167	363.323334	-1.184396	0.0191574	WBSCR16|LOC653375
0.00001	0.000214	1472.59253	697.963257	-1.180098	0.0150309	TFB1M
0.000028	0.00278	1105.75452	444.593048	-1.420374	0.0059309	FAM19A5
0.000036	0.004152	1040.77185	501.003754	-1.160282	0.0217663	CDH19
0.000402	0.039586	626.768921	285.878113	-1.238503	0.0172552	SPATA6
0.000012	0.000185	1379.31091	718.041626	-1.043357	0.0278563	SYNJ2
0.005903	0.332759	395.136932	182.617783	-1.18014	0.029842	FAM115C
0.000011	0.001763	1450.58228	550.72168	-1.502891	0.0040766	ZNF839
0.000009	0.0001	1551.20068	813.653076	-1.027317	0.0289958	QKI
0.000002	0.000014	3087.72778	1309.87622	-1.298518	0.0072878	TMEM51
0.000272	0.064323	673.933838	261.97525	-1.462863	0.0068046	TMEM139
0.000003	0.000023	2307.93506	1148.29297	-1.07436	0.0215768	HEATR1
0.000018	0.004949	1244.10486	404.436249	-1.725338	0.0016341	XKR8|RNF216P1
0.000078	0.022297	953.778809	315.517639	-1.690847	0.0027904	MR1
0.039343	0.742224	318.625	131.943741	-1.29837	0.0474836	PTAFR

0.008488	0.441994	372.078552	167.319962	-1.208454	0.0283259	ADAP2
0.	0.000001	16628.2422	6571.37207	-1.287421	0.0065565	AK4
0.000678	0.166391	570.171082	267.711945	-1.193691	0.0476576	CSF1
0.000001	0.000013	3764.80591	1340.47192	-1.552579	0.0022282	ZDHHC3
0.000001	0.000005	3580.33887	1859.64185	-1.002564	0.0281566	PARD3
0.000441	0.041211	616.287842	284.921997	-1.218909	0.0189822	MLKL
0.061885	0.976724	264.123352	89.874718	-1.552621	0.0132716	OR4M2|OR4M1
0.003506	0.236117	430.772614	199.827835	-1.185764	0.0273697	POLR2J3|UPK3BL|POLR2J2
0.000001	0.000004	4266.8501	2007.83948	-1.140393	0.0144992	FXYD2|FXYD6-FXYD2
0.000508	0.129711	600.566223	228.511261	-1.482449	0.0069188	EGFL8|PPT2-EGFL8
0.004522	0.237484	412.954773	199.827835	-1.124415	0.0355866	LOC100507172
0.296611	0.998667	187.611435	65.97187	-1.473919	0.0258941	LOC100652736
